# The flying spider-monkey tree fern genome provides insights into fern evolution and arborescence

**DOI:** 10.1038/s41477-022-01146-6

**Published:** 2022-05-09

**Authors:** Xiong Huang, Wenling Wang, Ting Gong, David Wickell, Li-Yaung Kuo, Xingtan Zhang, Jialong Wen, Hoon Kim, Fachuang Lu, Hansheng Zhao, Song Chen, Hui Li, Wenqi Wu, Changjiang Yu, Su Chen, Wei Fan, Shuai Chen, Xiuqi Bao, Li Li, Dan Zhang, Longyu Jiang, Dipak Khadka, Xiaojing Yan, Zhenyang Liao, Gongke Zhou, Yalong Guo, John Ralph, Ronald R. Sederoff, Hairong Wei, Ping Zhu, Fay-Wei Li, Ray Ming, Quanzi Li

**Affiliations:** 1 State Key Laboratory of Tree Genetics and Breeding, Chinese Academy of Forestry, Beijing, China; 2 Shenzhen Branch, Guangdong Laboratory for Lingnan Modern Agriculture, Genome Analysis Laboratory of the Ministry of Agriculture and Rural Affairs, Agricultural Genomics Institute at Shenzhen, Chinese Academy of Agricultural Sciences, Shenzhen, China; 3 State Key Laboratory of Bioactive Substance and Function of Natural Medicines; NHC Key Laboratory of Biosynthesis of Natural Products; CAMS Key Laboratory of Enzyme and Biocatalysis of Natural Drugs, Institute of Materia Medica, Chinese Academy of Medical Sciences & Peking Union Medical College, Beijing, China; 4 Thompson Institute, Ithaca, NY USA; 5 Plant Biology Section, Cornell University, Ithaca, NY USA; 6 Institute of Molecular & Cellular Biology, National Tsing Hua University, Hsinchu, Taiwan; 7 Beijing Key Laboratory of Lignocellulosic Chemistry, Beijing Forestry University, Beijing, China; 8 Department of Biochemistry and DOE Great Lakes Bioenergy Research Center, Wisconsin Energy Institute, University of Wisconsin, Madison, WI USA; 9 State Forestry Administration Key Open Laboratory on the Science and Technology of Bamboo and Rattan, Institute of Gene for Bamboo and Rattan Resources, International Center for Bamboo and Rattan, Beijing, China; 10 State Key Laboratory of Tree Genetics and Breeding, Northeast Forestry University, Harbin, China; 11 Beijing Advanced Innovation Center for Tree Breeding by Molecular Design, Beijing Forestry University, Beijing, China; 12 College of Landscape Architecture and Forestry, Qingdao Agricultural University, Qingdao, China; 13 GoldenGate International College, Tribhuvan University, Battisputali, Kathmandu, Nepal; 14 State Key Laboratory of Systematic and Evolutionary Botany, Institute of Botany, Chinese Academy of Science, Beijing, China; 15 Forest Biotechnology Group, Department of Forestry and Environmental Resources, North Carolina State University, Raleigh, NC USA; 16 College of Forest Resources and Environmental Science, Michigan Technological University, Houghton, MI USA; 17 Department of Plant Biology, University of Illinois at Urbana-Champaign, Urbana, IL USA

**Keywords:** Plant morphogenesis, Secondary metabolism, Phylogenetics, Genome evolution, Genetic variation

## Abstract

To date, little is known about the evolution of fern genomes, with only two small genomes published from the heterosporous Salviniales. Here we assembled the genome of *Alsophila spinulosa*, known as the flying spider-monkey tree fern, onto 69 pseudochromosomes. The remarkable preservation of synteny, despite resulting from an ancient whole-genome duplication over 100 million years ago, is unprecedented in plants and probably speaks to the uniqueness of tree ferns. Our detailed investigations into stem anatomy and lignin biosynthesis shed new light on the evolution of stem formation in tree ferns. We identified a phenolic compound, alsophilin, that is abundant in xylem, and we provided the molecular basis for its biosynthesis. Finally, analysis of demographic history revealed two genetic bottlenecks, resulting in rapid demographic declines of *A. spinulosa*. The *A. spinulosa* genome fills a crucial gap in the plant genomic landscape and helps elucidate many unique aspects of tree fern biology.

## Abstract (web summary)

The genome of the flying spider-monkey tree fern and investigations on stem formation and secondary metabolite biosynthesis promote our understanding of fern evolution and arborescence, facilitating the protection and utilization of this endangered species.

## Main

Land plants evolved 470 million years ago (Ma) from aquatic charophycean algae^1^ and have since transformed the terrestrial ecosystem. The body plan of land plants has undergone a series of developmental, biochemical and physiological adaptations, one of which is the appearance of vascular tissues. In seed plants, xylem, with thickened cell walls, provides the trunk with high water-conducting efficiency and strong structural support. Lignin is an essential component of xylem secondary cell walls—it not only gives mechanical support in fibre cells but also forms a hydrophobic surface in vessels to aid water transport^2^.

Outside of seed plants, the fern order Cyatheales is one of the few lineages having arborescent trunks. The fossil record of Cyatheaceae in Cyatheales is the richest in the Jurassic period, and the more recent diversification has given rise to an estimated 643 species in four genera^3^. Like most homosporous ferns, members of Cyatheaceae have large genomes (1C = 6.48–9.63 picogram) and a high chromosome base number (*X* = 69)^4^. However, in contrast to many other groups of ferns, recent polyploidy is rare in Cyatheaceae^5,6^.

Tree ferns also have high ornamental values and are regarded as a resource for natural products with pharmaceutical applications. Some metabolites have been identified as having anti-tumour and antibacterial activities in the tree fern *Alsophila spinulosa* (Cyatheaceae)^7–9^, but they probably represent only a small fraction of the total natural product diversity. Many tree fern species are also being overexploited, which, in combination with climate change, poses serious threats to their survival. A better understanding of their recent demographic history will help guide future conservation efforts.

In this study, we generated a chromosomal-scale genome assembly for the tree fern *A. spinulosa*. We characterized its genome in detail, including DNA methylation, repeat landscape and the history of whole-genome duplications (WGDs). We then carried out genome-powered investigations into vascular tissues and metabolic diversity in *A. spinulosa*. Finally, from genome resequencing data, we reconstructed the demographic history of *A. spinulosa*.

## Results and discussion

### Genome assembly and annotation

**Fig. 1:**
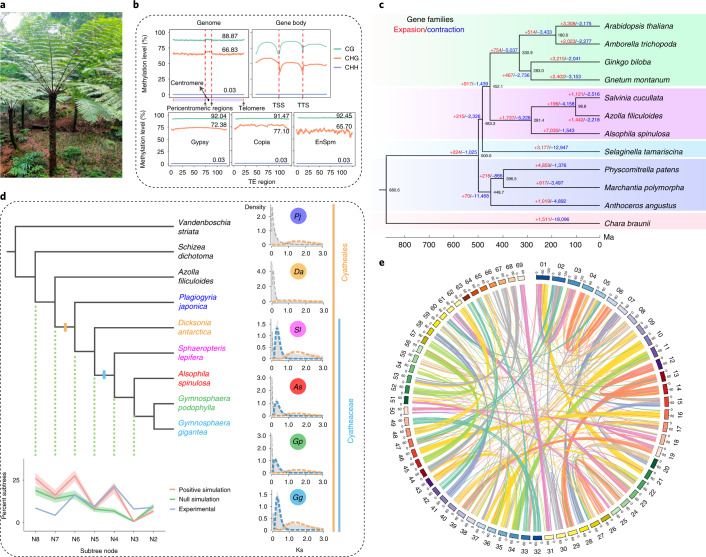
The *A. spinulosa* genome. **a**, *A. spinulosa*’s arborescent habit. **b**, DNA methylation levels of three contexts (CG, CHG and CHH) in the genome, gene body and TE (Gypsy, Copia and EnSpm) space. TSS, transcription start site; TTS, transcription termination site. **c**, Gene family expansion and contraction among 12 plant species, including 3 bryophytes, 3 ferns, 1 lycophyte and 4 seed plants, and 1 outgroup species, *Chara braunii*. The tree was constructed using 134 single-copy orthologous genes. The red and blue numbers above the branches represent expansion and contraction events, respectively. The number at each node represents divergence time. **d**, WGD analysis. The cladogram shows the relative phylogenetic positions of two ancient WGDs in *A. spinulosa* with Ks plots for each species in Cyatheales displayed along the right edge and a summary of experimental and simulated MAPS analyses below. The shaded area in the MAPS summary shows the standard deviation for the gene tree simulations. *Pj*, *Plagiogyria japonica*; *Da*, *Dicksonia antarctica*; *Sl*, *Sphaeropteris lepifera*; *As*, *A. spinulosa*; *Gp*, *Gymnosphaera podophylla*; *Gg*, *Gymnosphaera gigantea*. **e**, Intragenomic synteny among 69 chromosomes in the *A. spinulosa* genome. Source data

**Extended Data Fig. 1:**
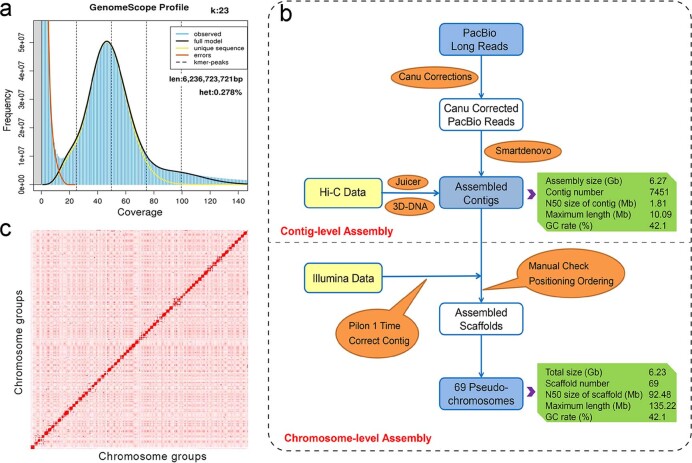
Genome assembly and annotation of *A. spinulosa*. **(a)** Estimation of genome size and heterozygosity based on 23-mer frequency distribution analysis using the GenomeScope software. The genome of *A. spinulosa* was estimated as 6.23 Gb with the heterozygosity of 0.28%. (**b)** Assembly strategy of *A. spinulosa* genome. PacBio long reads were assembled into contigs by the Smartdenovo software. Illumina short reads were used to correct contigs. Hi-C-based scaffolding was generated using 3D-DNA pipeline, and 69 pseudo-chromosomes were obtained. The final chromosome-level assembled genome size was 6.23 Gb with scaffold N50 size of 92.48 Mb. **(c)** Genome-wide interaction heat map of Hi-C links among chromosome groups (69 chromosomes). Each chromosome has higher intensity of interactions with itself than any other chromosomes (Darker red color means stronger interactions).

The genome of *A. spinulosa* (Fig. 1a) was estimated to be 6.23 Gb in size and had a heterozygosity of 0.28% (Extended Data Fig. 1). We conducted de novo genome assembly of *A. spinulosa* at a chromosome level based on 902 Gb (145× coverage) of corrected single-molecular real-time (SMRT) long reads, 386 Gb (62× coverage) of clean Illumina short reads and 399 Gb (63× coverage) high-throughput chromatin conformation capture (Hi-C) data (Supplementary Table 1). The assembled genome size was 6.27 Gb, with 6.23 Gb anchored to 69 pseudochromosomes, and N50 sizes were 1.80 Mb and 92.48 Mb, respectively, for contigs and scaffolds (Extended Data Fig. 1, Supplementary Table 2 and Supplementary Fig. 1). The mapping rates of Illumina and RNA-seq reads to the genome were 97.9% and 95.8%, respectively. Evaluation of the assembly based on the interspersed long terminal repeat (LTR) retrotransposons^10^ showed that the LTR assembly index score was 17.32, comparable to that of *Arabidopsis* (TAIR10). BUSCO (Benchmarking Universal Single-Copy Orthologs) assessment^11^ using the Eukaryota_odb10 database (10 September 2020) showed that 249 (97.6% of 255) complete BUSCO genes were covered in the assembly (Supplementary Table 3).

A total of 4.68 Gb was identified as repetitive sequences, with retrotransposons (2.52 Gb) as the main transposable elements (TEs). Within the LTR family, the Gypsy and Copia families were predominant, accounting for 24.91% and 12.47% of the genome (Supplementary Table 4). Gene prediction using the Geta pipeline on the repeat-masked genome resulted in 67,831 high-confidence protein-coding genes, of which 95.36% can be functionally annotated (Supplementary Tables 5 and 6). The average intron length was 11.46 times that in *Arabidopsis thaliana* (Supplementary Table 7). The predicted proteome included 72.1% complete and 21.6% fragmented BUSCO genes against the Eukaryota_odb10 database (Supplementary Table 3). We also performed small RNA sequencing in leaves and identified 182 known and 181 potentially new microRNAs (Supplementary Text).

### Genome evolution and genomic features

#### DNA methylation

Knowledge of DNA methylation in ferns is very limited^12^. Although angiosperm genomes generally exhibit high levels of gene body methylation (gbM), gbM in *Selaginella* and bryophytes is apparently rare^12,13^. Here our whole-genome bisulfite sequencing (WGBS) in *A. spinulosa* leaves revealed an extremely high level (88.87%) of mCG and a high level (66.83%) of mCHG (H = A, T, C). However, the mCHH level was 0.03%, much lower than that in other plant species studied previously^14^ (Fig. 1b). In addition, we found clear evidence of gbM in *A. spinulosa*, most of which is under the CG context (Fig. 1b). Although gbM in ferns has been documented previously^15^, our genome-wide data provide a much better picture of its prevalence. Centromeric and pericentromeric regions had higher CHG methylation levels than other regions, and mCG and mCHG levels in Copia, Gypsy and EnSpm were also high (Fig. 1b), suggesting that repeats and TEs were highly methylated in *A. spinulosa*. The *A. spinulosa* genome had six DNA methyltransferase genes, including one *METHYLTRANSFERASE* (*MET*), two *CHROMOMETHYLASE* (*CMT*) and three *DOMAINS REARRANGED METHYLASE* (*DRM*) genes (Supplementary Fig. 2). Phylogenetic analysis showed that the two *A. spinulosa* CMTs were in the hCMTα clade (Supplementary Fig. 3) and were not orthologous to CMT3, which is linked to gbM in angiosperms^16^. How gbM takes place in *A. spinulosa* without CMT3 requires further functional studies.

#### Gene family evolution

We constructed a phylogenetic tree of 12 species, including 4 seed plants, 3 ferns, 1 lycophyte, 3 bryophytes and 1 outgroup (Fig. 1c). A total of 23,833 orthologous groups, covering 301,746 genes, were circumscribed. Gene-family evolution analysis identified 1,737 families expanded and 5,228 families contracted along the branch leading to ferns. Gene Ontology (GO) and Kyoto Encyclopedia of Genes and Genomes (KEGG) analyses of the 7,035 expanded gene families in *A. spinulosa* compared with the water ferns (*Azolla filiculoides* and *Salvinia cucullata*) resulted in 334 significantly enriched GO terms and 63 KEGG pathways (Supplementary Data 1). These include those involved in the biosynthesis of secondary metabolites, such as flavonoids, phenylpropanoids and terpenoids, which might be related to the natural product diversity in tree ferns. Consistently, *A. spinulosa* had higher gene numbers in the 11 monolignol pathway enzyme families than *A. filiculoides* and *S. cucullata* (Supplementary Table 8), in which eight families, *PAL*, *4CL*, *HCT*, *CSE*, *CCR*, *CCoAOMT*, *CAD* and *C3H*, had duplicated gene copies (Supplementary Table 9), implying that lignin biosynthesis is enhanced in *A. spinulosa*.

We observed a significant expansion in some transcription factor (TF) families, such as MYB, NAC, bHLH and MADS-box, compared with the two water ferns (Supplementary Table 10). However, compared with *A. thaliana*, the MADS-box genes involved in flowering (including *FLC*, *SOC1*, *SEP*, *AP3*/*PI*, *AG* and *AP1*/*FUL*) were absent in *A. spinulosa* (Supplementary Fig. 4). *YABBY*, which encodes a key TF regulating leaf morphogenesis in angiosperms, is absent in the water ferns^17^ and *Selaginella moellendorffii*^18^, but present in the lycophyte species *Huperzia selago*^19^ and hornworts^20^. We could not identify a *YABBY* orthologue in *A. spinulosa*, supporting the idea that *YABBY* has been lost at least three times in land plant evolution (in setaphytes, *Selaginella* and ferns). *NOP10* is a crucial gene for female gametophyte formation in flowers^21^. This gene can be found in bryophytes and *S. cucullata*, but not in *A. filiculoides*, *A. spinulosa* or *Ginkgo biloba* (Supplementary Fig. 5), suggesting a dynamic evolutionary history. The genome assembly of *A. spinulosa* will aid future studies on gene family evolution across land plants.

#### History of WGD

**Extended Data Fig. 2:**
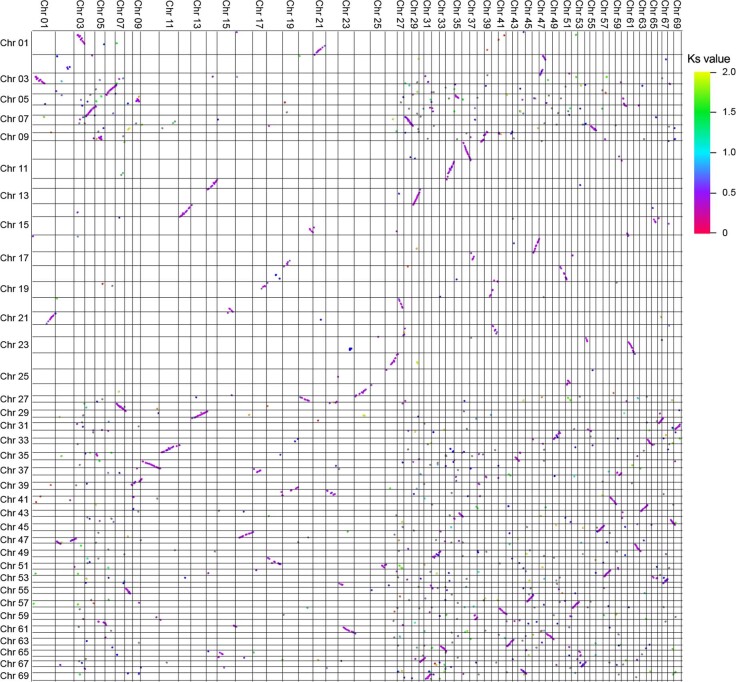
Self-self synteny in *A. spinulosa*. Synteny was assessed using the MCSCANX program to identify collinear blocks of syntenic gene pairs. The resulting syntenic blocks were filtered by median Ks using a custom Python script to select collinear gene pairs that were the result of a specific duplication event. Blocks of syntenic genes show conservation of genes order following WGD. Synteny regions are color coded by Ks value.

Two putative WGD events were identified in *A. spinulosa* using a combination of methods, including synonymous substitutions per site (Ks), synteny analysis and phylogenetic reconciliation. Mixture modelling of the Ks data provided evidence for two separate WGD events with peaks centred on Ks = 0.3 and Ks = 1.5. Likewise, evidence from synteny provided a high degree of support for the more recent WGD with 7,766 genes in 264 collinear blocks with a median Ks between 0.2 and 0.5 (Fig. 1d,e and Extended Data Fig. 2).

**Extended Data Fig. 3:**
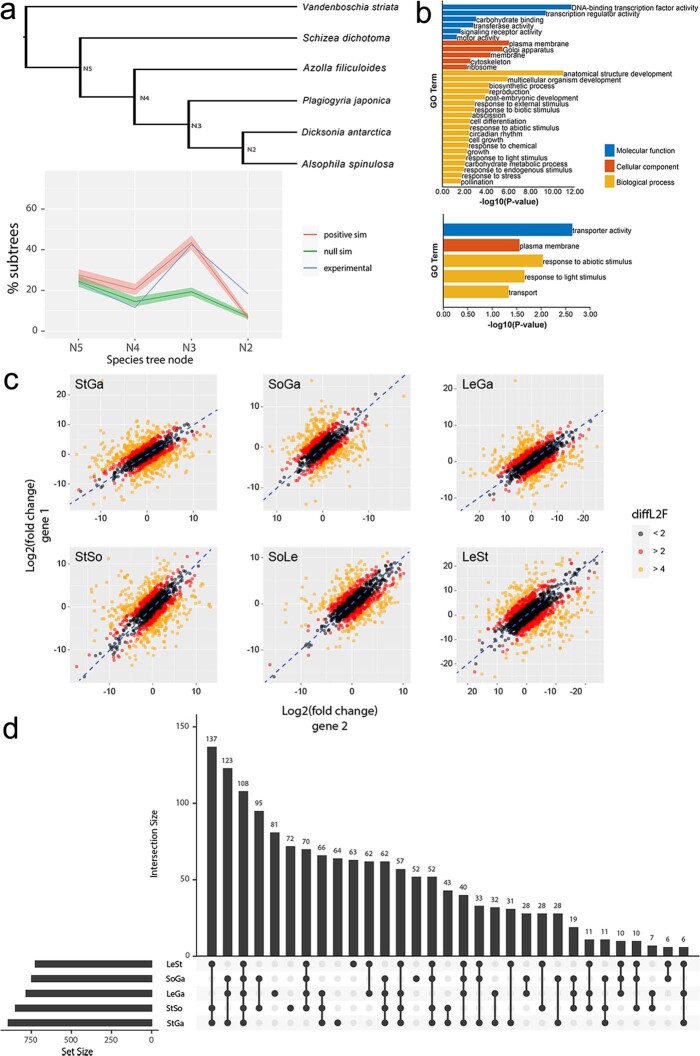
WGD analysis and gene functional prediction of *A. spinulosa*. **(a)** Summary of second MAPS analysis focusing on the WGD event common to all Cyatheales. Shaded area shows the standard deviation for gene tree simulations. The dark lines in the center of the shaded regions represent the average values for null and positive gene tree simulations. (**b)** GO enrichment of syntenic homoeologs. The first histogram shows GO enrichment of all syntenic pairs from the most recent WGD event (0.2<Ks<0.5) compared to genomic background. The second histogram shows GO enrichment of differentially expressed genes relative to a background of all syntenic gene pairs from the most recent WGD event. P-values were obtained using an one-sided hypergeometric test. **(c)** Gene pairs plotted according to log2 fold change (L2F) as calculated for gene 1 (x-axis) and gene 2 (y-axis) in DESeq2. Each point represents one gene pair with pairs colored according to the difference in L2F values (diffL2F = |L2F_1 - L2F_2|) to visualize the arbitrary cutoffs of diffL2F = 2 and diffL2F = 4. Blue dashed lines represent zero difference expression between homoeologs. St: stem; So: sorus; Le: leaf; Ga: gametophyte. **(d)** Upset plot showing overlap in the number of homoeologous gene pairs that are differentially expressed between various comparisons. Source data

Additional Ks plots constructed using transcriptome data from other tree fern species in *Gymnosphaera* and *Sphaeropteris* exhibited similar distributions to those made using the *A. spinulosa* genome. Thus, the most recent WGD event is probably shared between all members of Cyatheaceae. This ‘Cyatheaceae WGD’ event (N4) was corroborated by gene-tree species-tree reconciliation, verified by comparison with null and positive simulations of gene-tree evolution (Fig. 1d). Further analysis found similar support for a more ancient ‘Cyatheales WGD’ event in addition to the more recent WGD shared among Cyatheaceae (Extended Data Fig. 3).

**Extended Data Fig. 4:**
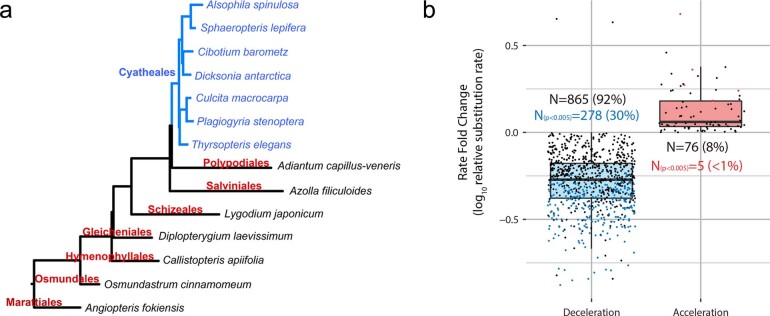
Genome-wide deceleration of nucleotide substitution rate in Cyatheales ferns. **(a)** Phylogenetic tree generated by OrthoFinder depicts the relationships of 14 species from eight orders, including Cyatheales, Polypodiales, Salviniales, Schizeales, Gleicheniales, Hymenophyllales, Osmundales and Marattiales. The branch lengths within Cyatheales are shorter than those in other orders, suggesting deceleration of substitution rate in Cyatheales. (b) Genome-wide substitution rate variation in Cyatheales (N is the number of nuclear protein-coding genes). Among the 941 single-copy nuclear genes from Cyatheales, a majority (92%) showed reduced substitution rates, and the reduction in 30% genes was statistically significant (p <0.005). By contrast, <1% of genes had significant elevated rates. Upper bound of each box (Q3) represents the 75th percentile, lower bound of each box (Q1) represents the 25th percentile, the midline of each box is the median, and each whisker represents the highest or lowest point within Q3 + 1.5*IQR or Q1 - 1.5*IQR, respectively (IQR = Q3 - Q1). P-values were calculated using an one-sided likelihood-ratio test. Source data

The preservation of synteny following the most recent ‘Cyatheaceae WGD’ is remarkable given Cyatheaceae’s crown age of 108.63–170.86 Myr^22^—roughly the same period at which monocots and dicots diverged. Such preservation might be associated with the slow rate of evolution in tree ferns. Previous research has found a sudden decrease in chloroplast nucleotide substitution rate that is tied to the origin of arborescence in ferns^23^. Here we were able to further show that the deceleration is genome-wide in *A. spinulosa* and not restricted to the chloroplast genome (Extended Data Fig. 4). It is possible that arborescence might also be correlated with the extremely slow process of diploidization in *A. spinulosa*. Further investigation is required to determine whether gene order has been so strictly maintained in other members of the Cyatheaceae and other non-arborescent ferns. In any case, it is clear that genome evolution after WGD has followed quite different trajectories in *A. spinulosa* and angiosperms.

#### Divergent expression of homoeologues

To understand how duplicated gene pairs (that is, homoeologues) diverge in expression following WGD, we conducted differential expression analysis using RNA-seq data from stem, leaf, sorus and gametophyte tissues. We found that homoeologous gene pairs in *A. spinulosa* have undergone substantial differentiation in gene expression following duplication. Of the syntenic gene pairs resulting from the most recent WGD (Ks between 0.2 and 0.5), over half exhibit at least a fourfold difference in expression with regard to tissue type and/or mean expression level (Extended Data Fig. 3). This result is consistent with previous work demonstrating that WGD often precipitates large-scale shifts in gene expression^24–26^. Although we did not find evidence of expression bias between collinear blocks of genes on different chromosomes, our lack of information regarding the polyploid ancestor may obscure evidence of expression level dominance or homoeologue expression bias in *A. spinulosa*.

### Development of vascular tissue in woody trunk of *A. spinulosa*

#### Anatomy of vascular tissue in stem

**Fig. 2:**
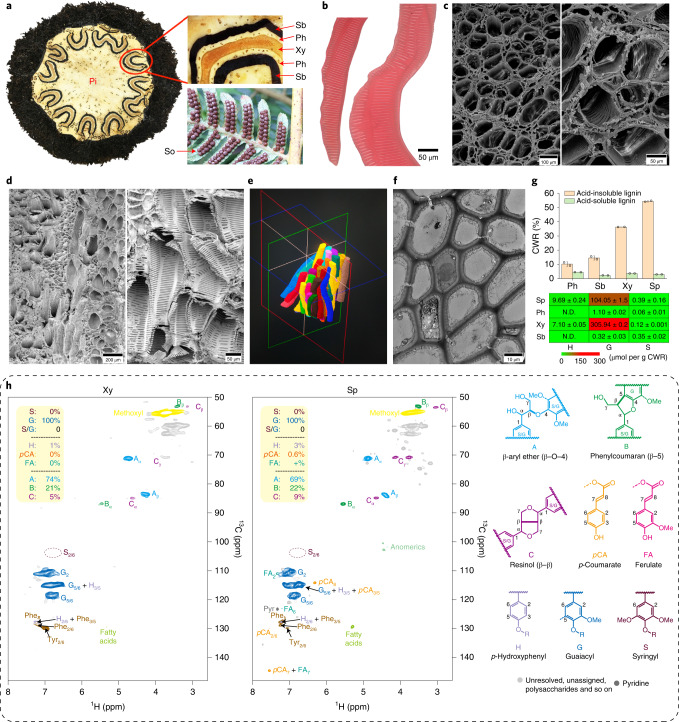
Vascular bundle structure and lignin biosynthesis in *A. spinulosa*. **a**, A stem cross-section and mature sori (So) underneath the leaf. A wavy structure is enlarged to show the xylem (Xy), phloem (Ph) and sclerenchymatic belt (Sb) in the vascular bundle. Pi, pith. **b**, Segregated xylem cells showing scalariform thickening. **c,d**, Scanning electron micrographs of xylem for cross-section (**c**) and longitudinal section (**d**). **e**, microCT shows the three-dimensional arrangement of tracheids. **f**, Transmission electron microscopy (TEM) image of Sb. The microscopic observations in **b**–**d**,**f** are more than ×6. **g**, The histogram (top) shows the content of acid-soluble lignin and acid-insoluble lignin in Ph, Sb, Xy and spores (Sp), calculated as percentage of cell wall residue (CWR). The heat map (bottom) shows the content of lignin aromatic units (G, S and H) from the three canonical monolignols in Ph, Sb, Xy and Sp. The values are the mean ± s.d. of two independent experiments. **h**, Heteronuclear single-quantum coherence NMR spectra, showing that guaiacyl units are major components of lignins in Xy and Sp. Relative quantification was performed using the correlation peak volume integration (uncorrected). Side chain units are on the basis A + B + C = 100%; aromatics are on the basis S + G = 100% as H peaks overlap. *p*CA, *p*-coumarate; FA, ferulate; Phe and Tyr are phenylalanine and tyrosine units (in protein). Source data

To investigate the development of woody trunk in tree ferns, we performed anatomical observations on the xylem, phloem and sclerenchymatic belt that comprise the vascular bundle in stems (Fig. 2a). The cells were segregated for these tissues, and under a microscope we could observe lignin only in xylem cells (based on the lignin stain safranine; Fig. 2b). We did not observe the perforation on the end walls, indicating that these cells are tracheids (Fig. 2b). The average length of the tracheids was 1.48 ± 0.18 cm, as measured by microscopy. Under scanning electron microscopy (SEM), tracheids exhibited scalariform thickening in their whole walls, and they were arranged next to each other (Fig. 2c,d). Using X-ray computed microtomography (microCT), we made three-dimensional reconstructions and observed that tracheids had irregular (crooked) shapes (Fig. 2e), consistent with the observations under SEM. These tracheids were bundled together closely, augmenting mechanical strength for support. The wall thickness of cells in the sclerenchymatic belt was measured as 1.86 ± 0.21 µm, about two times that of pith parenchyma cells (0.95 ± 0.12 µm) (Fig. 2f), indicating that the sclerenchymatic belt may also confer stem support.

#### Lignin accumulation in stems

Lignified cell walls provide superior structural support and are considered a key innovation during the evolution of vascular plants. Characterization of lignin structure in seed-free plants is limited^27^. We determined the lignin content and composition in stems using the classical Klason method, which detected a high lignin content in xylem, at a level of 39.92% of cell wall residue, the fraction of the biomass remaining after the removal of extractives by simple but extensive solvent extraction (Fig. 2g and Supplementary Table 11). Analytical thioacidolysis showed that xylem contained mainly guaiacyl (G) lignin units and only trace levels of *p*-hydroxyphenyl (H) and syringyl (S) lignins, as was confirmed by nuclear magnetic resonance (NMR) spectroscopy (Fig. 2h). Both thioacidolysis and NMR analysis detected very trace amounts of G and S lignin in phloem and the sclerenchymatic belt (Supplementary Table 11 and Supplementary Fig. 6), suggesting that the lignin contents in these tissues measured by the Klason method may be an artefact due to attributing to other similar compounds such as aromatics and protein residues^28^. Consistent with the segregation analysis (Fig. 2b), chemical composition analyses showed that G lignins were mainly accumulated in xylem.

The low level of S lignin in *A. spinulosa* is in stark contrast to what was found in the lycophyte *Selaginella*. *Selaginella* had independently evolved S lignin by recruiting enzymes that are not part of the canonical biosynthetic pathway^29,30^. Although S lignin is rich in *Selaginella*, it is in the cortex, and G lignin is predominantly deposited in the transporting tissues^27,29,31^. A broader survey of lignin composition in ferns and lycophytes, especially those with arborescent habits, is needed to better understand the adaptive roles of lignin outside of seed plants.

#### Genes associated with xylem development

**Fig. 3:**
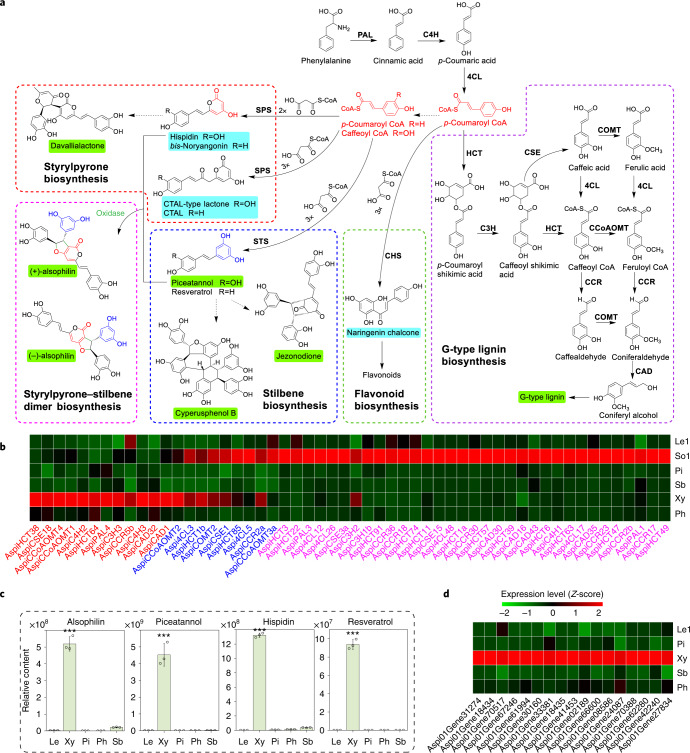
Biosynthesis of phenylpropanoid-based metabolites in *A. spinulosa*. **a**, Biosynthetic pathways of lignin, flavonoids, stilbene, styrylpyrone and alsophilin. The metabolites shaded in green are identified in our metabolomic characterizations. The metabolites shaded in blue are products in the enzyme assays. PAL, phenylalanine ammonia-lyase; C4H, cinnamate-4-hydroxylase; 4CL, 4-coumarate:coenzyme A ligase; HCT, *p*-hydroxycinnamoyl-CoA:quinate shikimate *p*-hydroxycinnamoyltransferase; CCR, cinnamoyl CoA reductase; C3H, 4-coumarate 3-hydroxylase, CAD, cinnamyl alcohol dehydrogenase; CSE, caffeoyl shikimate esterase; COMT, caffeic acid/5-hydroxyconiferaldehyde *O*-methyltransferase; CCoAOMT, caffeoyl-CoA *O*-methyltransferase. **b**, Heat map showing gene expression profiles of monolignol biosynthetic pathway genes in xylem, phloem, sclerenchymatic belt, pith, sorus stage 1 and leaf stage 1. Genes highlighted in red and pink are significantly upregulated in xylem and sorus, respectively, and genes highlighted in blue are significantly upregulated in both xylem and sorus. **c**, Relative content of alsophilin, piceatannol, hispidin and resveratrol in leaf, xylem, phloem, pith and sclerenchymatic belt of *A. spinulosa*, determined by ultra performance liquid chromatography-mass spectrometry (UPLC–MS). The asterisks indicate the significance (***P < 0.001, two-sided Student’s *t*-test) of alsophilin content in Xy compared with the other four tissues. The values are the means ± s.d. of three independent experiments. **d**, Heat map showing the gene profiles of 17 oxidase genes upregulated in xylem. The FPKM values were normalized using the *Z*-score method. So1, sorus stage 1; Le1, leaf stage 1. Source data

**Extended Data Fig. 5:**
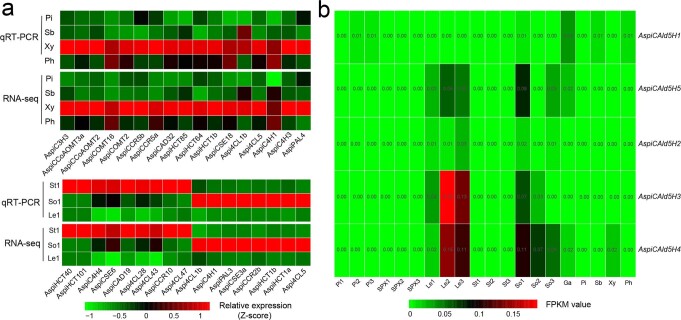
Expression patterns of monolignol biosynthetic genes in different tissues of *A. spinulosa*. **(a)** The first heat map shows FPKM values of 17 monolignol biosynthetic genes in pith (Pi), sclerenchymatic belt (Sb), xylem (Xy), and phloem (Ph) by RNA-seq analysis and their transcript abundance by qRT-PCR analysis. The second heat map shows FPKM values of 17 monolignol biosynthetic genes in St1 (stem stage 1), So1 (sorus stage 1), Le1 (leaf stage 1) by RNA-seq analysis and their transcript abundance by qRT-PCR analysis. Transcript abundance and FPKM values were normalized using the Z-score method. **(b)** Heat map shows FPKM values of five *CAld5H* genes in different tissues of *A. spinulosa*. Pi1/2/3 (pith stage 1/2/3), SPX1/2/3 (wavy structure stage 1/2/3), So1/2/3 (sorus stage 1/2/3), St1/2/3 (stem stage 1/2/3), Le1/2/3 (leaf stage 1/2/3), Ga (gametophyte). All gene accession numbers are shown in Supplementary Data 3. Source data

We performed RNA-seq analysis and obtained 988 differentially expressed genes (DEGs) in xylem compared with pith, sclerenchymatic belt, phloem and leaf (greater than twofold change and *q* < 0.01), among which 64 were TFs (Supplementary Data 2). We first examined the lignin pathway genes in xylem. Among the 395 gene models in 11 enzyme families of monolignol biosynthesis (Fig. 3a and Supplementary Data 3), 79 genes were highly expressed in xylem, and 21 genes were significantly upregulated in xylem (Fig. 3b and Supplementary Data 4). Among the 21 genes, *AspiPAL4*, *4CL3*, *4CL5*, *CAD1*, *CCR2a*, *COMT2*, *CSE1*, *HCT1b*, *C3H3*, *C4H2*, *C4H3*, *CCoAOMT1*, *CCoAOMT2* and *CCoAOMT3a* encode the orthologues of the essential enzymes of lignin biosynthesis in poplar (Supplementary Data 3), suggesting the roles of these 14 genes in lignin biosynthesis in xylem. Whether other xylem-differentially-expressed putative phenylpropanoid genes (Supplementary Data 4) are involved in lignin biosynthesis needs further investigations, such as enzyme assays. Quantitative PCR with reverse transcription on selected genes confirmed the RNA-seq results (Extended Data Fig. 5). All members in the *CAld5H* family, encoding key enzymes for S monolignol biosynthesis, were expressed at an extremely low level in xylem (Extended Data Fig. 5). RNA-seq analysis indicated that *A. spinulosa* shares with gymnosperms and angiosperms a conserved set of enzymes responsible for the formation of G lignin, and the trace of S lignins in xylem is due to the low expression of *CAld5H* genes. In *G. biloba*, S lignin is also absent in wood but can be detected in cell cultures^32^. Traces of S lignin in several tissues and lower expression levels of *CAld5Hs* in *A. spinulosa* indicate that *CAld5H* genes may be repressed in this species.

NAC-domain TFs have been identified as key regulators in the formation of vascular tissues^33,34^. In the moss *Physcomitrella patens*, which lacks vasculature, the differentiation of both hydroid cells and stereid cells is regulated by NAC proteins, demonstrating that NAC proteins contribute to the evolution of both water-conducting and supporting cells in moss gametophytes^35^. In *A. thaliana*, VASCULAR-RELATED MAC-DOMAIN (VND) proteins regulate vessel differentiation^36–38^, and NAC SECONDARY WALL THICKENING PROMOTING FACTOR (NST)/SECONDARY WALL-ASSOCIATED NAC DOMAIN PROTEIN (SND) proteins regulate fibre differentiation^39^. In *Pinus taeda*, four *VNS*s (VND, NST/SND and SMB-related) were identified to regulate the formation of tracheids^40^, the only type of cells with secondary cell wall thickening in xylem for both support and transport in gymnosperms. Here we found seven SMB orthologues and two VND orthologues in the *A. spinulosa* genome (Supplementary Fig. 7). Among the nine NACs, the two *VND*s (Aspi01Gene53944 and Aspi01Gene03119), which had the highest similarity with *AtVND6*, were the only NACs that were significantly upregulated in xylem compared with phloem, pith and sclerenchymatic belt (Supplementary Fig. 7 and Supplementary Data 5). These two VNDs are therefore probably key regulators for the formation of tracheids that serve both support and transport functions in *A. spinulosa*’s arborescent trunks.

### Lignin biosynthetic and pathway genes in spores

We also detected a higher content of lignin, exclusively composed of the guaiacyl units, in mature spores (Fig. 2g). As in xylem, all *CAld5H* members had an extremely low transcript abundance in spores (Extended Data Fig. 5), which was in agreement with the scarcity of S lignin. RNA-seq showed more monolignol pathway gene members expressed in spores than in xylem (Fig. 3b and Supplementary Data 6), indicating that additional genes participate in lignin biosynthesis. Some catalytic steps in the monolignol pathway apparently recruited the same enzyme between xylem and spores, such as AspiCOMT2 in the 5-*O*-methylation of phenolic hydroxyl groups on the aromatic ring. However, some steps involved different enzyme family members between xylem and spores, such as AspiC4H2 in xylem and AspiC4H1 in spores.

### (±)-Alsophilin, a pair of hispidin–piceatannol heterodimers

**Extended Data Fig. 6:**
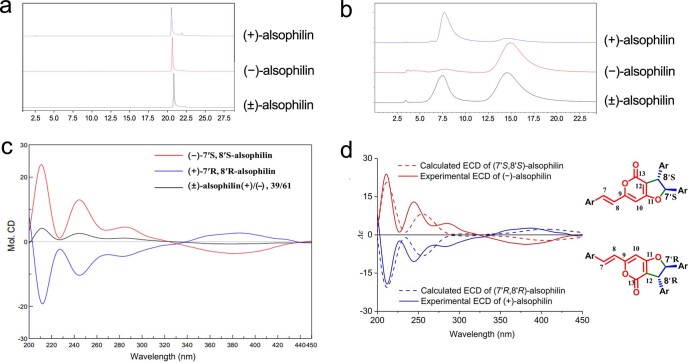
HPLC analysis and ECD calculation for the enantiomers (±)-alsophilin. (a) HPLC analysis of (±)-alsophilin, (−)-alsophilin, and (+)-alsophilin on a C18 OSAKA SODA CAPCELL PAK column (150 × 4.6 mm I.D., 5 µm) using water (solvent A) and acetonitrile (solvent B) as gradient eluent (0-30 min, 10%-50% B; 30-35 min, 50%-100% B), flow rate 1 ml/min, at 382 nm. (b) HPLC analysis of (±)-alsophilin, (−)-alsophilin, and (+)-alsophilin on a chiral column Daicel Chiralpak IC column (250 × 4.6 mm I.D., 5 µm) using isopropyl and hexane as eluent (60:40) at a flow rate of 1 ml/min. (c) Circular dichroism (CD) spectra of (±)-alsophilin, (−)-alsophilin, and (+)-alsophilin in MeOH, measured using JASCO J-815 CD spectro polarimeters. (d) Comparison of the calculated ECD spectra for (7′S,8′S)-alsophilin and (7′R,8′R)-alsophilin with the experimental spectra of (−)-alsophilin and (+)-alsophilin in MeOH. Energies of the conformers of (+)-alsophilin at B3LYP/6-311g (d,p) in MeOH are shown in Supplementary Table 20.

We used the widely targeted metabolome method^41^ to better capture the diversity of secondary metabolites in *A. spinulosa* (Supplementary Text). A total of 187 secondary metabolites were identified, including flavonoids, phenylpropanoids and alkaloids from stems and leaves (Supplementary Text and Supplementary Fig. 8). We then carried out extraction and isolation of metabolites from stems and obtained 11 purified compounds. Ten compounds were identified as known phenolics by comparing their spectroscopic data with those in previous reports (Supplementary Text). One new compound was named alsophilin, and its structure was characterized by mass spectrometry (MS) and NMR (Supplementary Text and Supplementary Fig. 9). On the basis of their electronic circular dichroism spectra, the compound was identified as a racemic pair of heterodimer enantiomers, (−)-alsophilin and (+)-alsophilin (Extended Data Fig. 6). Quantification of alsophilin in leaves and different parts of stems suggested that it was primarily synthesized in xylem (Fig. 3c).

**Extended Data Fig. 7:**
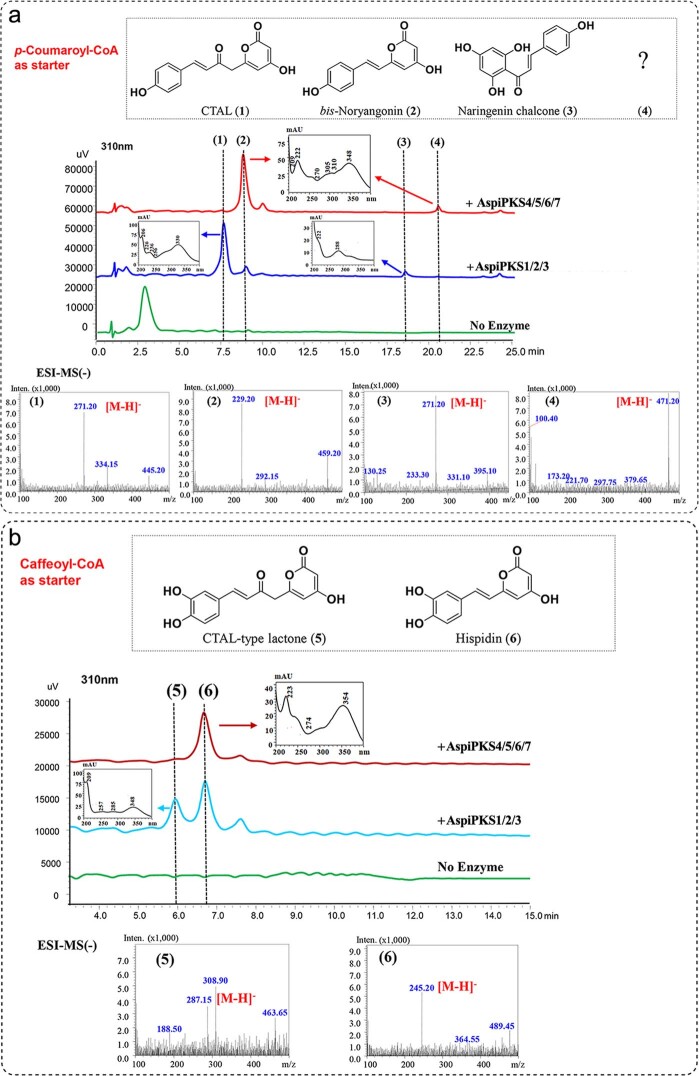
*In vitro* enzyme activity assays of seven PKS III proteins, including AspiPKS1/2/3/4/5/6/7. **(a)** Assays were conducted using *p*-coumaroyl-CoA and malony-CoA as substrates, and products were analyzed using LC-MS extracted ion chromatograms (XICs). Naringenin chalcone and coumaroyltriacetic acid lactone (CTAL) (271.20 m/z) and bis-noryangonin (229.20 m/z) are products for AspiPKS1, 2 and 3. Bis-noryangonin (229.20 m/z) and peak 4 (471.20 m/z) are products for AspiPKS4, 5, 6 and 7. **(b)** Assays were conducted using caffeoyl-CoA and malony-CoA as substrates. CTAL-type lactone (287.15 m/z) and hispidin (245.20 m/z) are products for AspiPKS1, 2, and 3. Hispidin is the product for AspiPKS4, 5, 6, and 7.

The structure of alsophilin represents an unprecedented phenolic compound derived from hispidin and piceatannol, which belong to the styrylpyrone and stilbene families, respectively. Two kinds of plant type III polyketide synthases (PKS III), styrylpyrone synthase (SPS) and stilbene synthase (STS), were reported to catalyse hydroxycinnamoyl-CoA reactions to synthesize styrylpyrone and stilbene, respectively^42,43^. We performed blastp searches using *Piper methysticum* SPS^42^ and *Vitis vinifera* STS^43^ as the queries, and both identified the same 103 genes, encoding PKS III in the *A. spinulosa* genome (Supplementary Fig. 10). From these 103 genes, we selected 8 that were highly expressed in xylem to produce recombinant proteins for in vitro enzyme assays using both *p*-coumaroyl-CoA and caffeoyl-CoA as substrates. Seven enzymes had detectable activities. The recombinant proteins AspiPKS4, 5, 6 and 7 could catalyse *p*-coumaroyl-CoA to bis-noryangonin and catalyse the conversion of caffeoyl-CoA to hispidin (Extended Data Fig. 7), demonstrating that these four proteins perform SPS functions of adding two molecules of malonyl-CoA to the two substrates. Three proteins, AspiPKS1, 2 and 3, not only displayed SPS activities (converting *p*-coumaroyl-CoA to coumaroyltriacetic acid lactone and bis-noryangonin) but also had chalcone synthase (CHS) activities owing to their synthesis of naringenin chalcone (Fig. 3a and Extended Data Fig. 7). We could not detect any STS activities for piceatannol synthesis. It is possible that *Escherichia coli* recombinant proteins lack post-translational modification as reported to be required for PKS activities^44^. *A. spinulosa* has more PKS III members than *A. thaliana*, implicating the abundance of related metabolites in *A. spinulosa*. We detected three AspiPKSs that had both SPS and CHS activities, supporting the notion that many enzymes are promiscuous^45^. It is accepted that gene duplication followed by sequence divergence is a key evolutionary mechanism to generate a new or specific enzyme functions^45^. Among the PKS III enzymes, STSs seem to have evolved from CHSs several times independently^43^. The evolution of PKS proteins in ferns needs further investigation.

The cross-coupling of hispidin and piceatannol probably requires an oxidase, many of which are available for oxidizing phenolics in lignification. Among the 988 DEGs in xylem (Supplementary Data 2), 17 genes encoded oxidases, including peroxidases and polyphenol oxidases, and are the prime candidates for future characterizations (Fig. 3d).

### Resequencing of *A. spinulosa* populations

#### Genetic variation and population structure

**Fig. 4:**
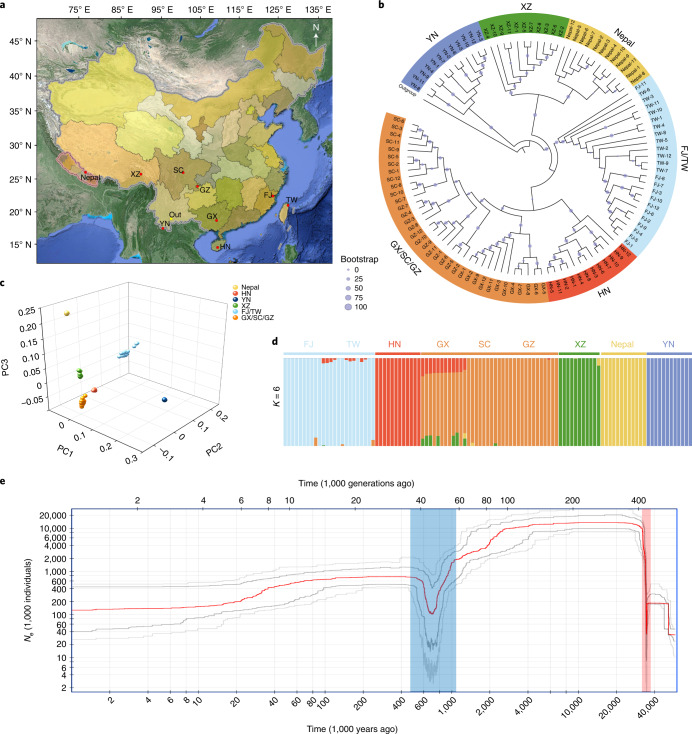
Phylogenetic relationships and structure of *A. spinulosa* populations. **a**, Geographic distribution of 107 *A. spinulosa* individuals in nine locations, including Yunnan (YN), Nepal, Xizang (XZ), Fujian (FJ), Taiwan (TW), Hainan (HN), Guangxi (GX), Sichuan (SC) and Guizhou (GZ), with *A. costularis* in YN as an outgroup (Out). **b**, A phylogenetic tree of 107 accessions constructed using the whole-genome SNPs. All accessions were clustered into six groups: YN, XZ, Nepal, FJ/TW, HN and GX/SC/GZ. The sizes of the dots on the nodes are proportional to bootstrap support values. **c**, PCA, with the proportion of the variance explained being 85.8% for PC1, 13.2% for PC2 and 12.4% for PC3. The dots are coloured corresponding to the colours in **b**. **d**, Cross-validation error shows that *K* = 6 is the optimal population clustering group. The structures are coloured corresponding to the colours used in **b**. **e**, Demographic history of *A. spinulosa*. The stairway plot shows the historical effective population size *N*_e_ (*y* axis) with a generation time of 100 years. The blue and red shadows represent two bottlenecks. The red line represents median of effective population size based on a subset of 200 inferences. Dark gray and light gray lines represent 75% and 95% confidence intervals, respectively.

To explore the genetic diversity and population structure, we resequenced 107 diverse *A. spinulosa* accessions from nine locations (Fig. 4a and Supplementary Data 7) and identified 93.86 million high-confidence variable sites, including 86,926,221 single nucleotide polymorphisms (SNPs), 3,657,912 insertions and 3,259,116 deletions, averaging 10.91 variants per kb (Supplementary Table 12). Our phylogenetic analysis clustered 107 accessions into six distinct groups (Fig. 4b). The population structures generated by phylogenetic analysis were supported by principal component analysis (PCA) and admixture analysis (Fig. 4c,d). A couple of intraspecific introgression events were detected; for instance, some individuals from Guangxi showed mixed components from Hainan and Xizang (Fig. 4d).

The ratios of non-synonymous to synonymous SNPs in these populations ranged from 1.43 to 1.75, and the nucleotide diversities (*π*) ranged from 6.46 × 10^−5^ to 6.29 × 10^−4^ (Supplementary Tables 13 and 14). The Yunnan population has the highest genetic diversity (*π* = 6.29 × 10^−4^ in Yunnan versus 1.42 × 10^−4^ on average in the other populations), suggesting that Yunnan province in China is probably a centre of diversity for this species and where future conservation efforts could focus.

#### Evolutionary history and selective sweeps

We investigated the demographic history of *A. spinulosa* by calculating historical effective population size (*N*_e_) and identified two bottlenecks, occurring at about 35.6–34.5 and 2.5–0.7 Ma (Fig. 4e).

**Extended Data Fig. 8:**
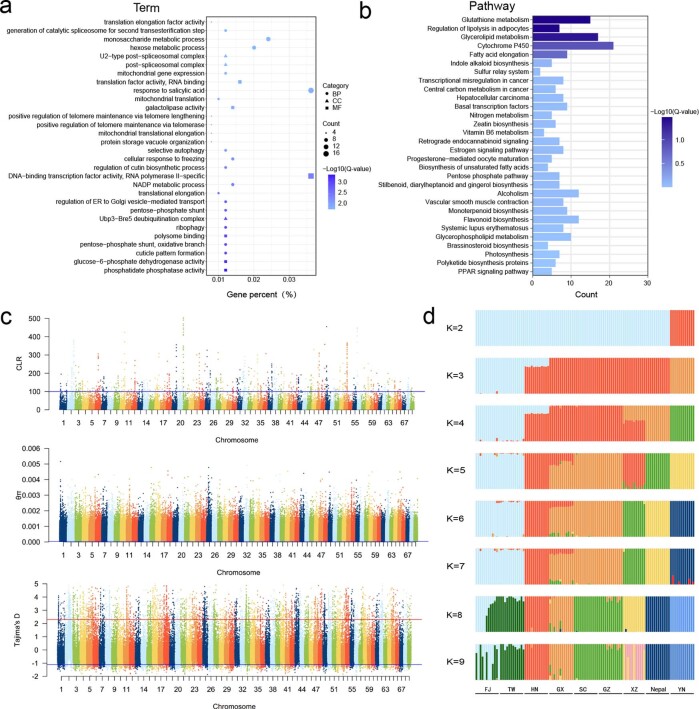
Resequencing of 107 *A. spinulosa* accessions. **(a)** GO enrichment of the protein-coding genes that undergo nature selection. **(b)** KEGG enrichment of the protein-coding genes that undergo nature selection. **(c)** Genome-wide distribution of CLR, θπ, and Tajima’s *D* values of 107 populations along 69 chromosomes in *A. spinulosa* genome. The blue dashed line represents the threshold of the top 5% CLR, the bottom 5% θπ, and the bottom 2.5% Tajima’s *D*, the red dashed line represents the threshold of the top 2.5% Tajima’s *D*. **(d)** The different ancestral population structures are estimated from the same variants set with STRUCTURE software using ancestral population sizes K=2-9. The parameter standard errors are estimated using bootstrapping (100 replicates).

We identified 225.23 Mb of selectively swept genomic regions using θπ, Tajima’s *D* and composite likelihood ratio (CLR) analyses. These regions were randomly distributed across 69 chromosomes in *A. spinulosa* and contained 2,553 protein-coding genes. Functional annotation using GO showed that these genes were significantly enriched in a series of basic biological processes (*q* < 0.05), including phosphatidate phosphatase activity, glucose-6-phosphate dehydrogenase activity, mitochondrial genome maintenance and regulation of DNA recombination (Extended Data Fig. 8 and Supplementary Table 15). Three significantly enriched KEGG pathways were regulation of lipolysis in adipocytes, glutathione metabolism and glycerolipid metabolism (Extended Data Fig. 8 and Supplementary Table 15). To what extent these selectively swept genes contribute to adaptative evolution in *A. spinulosa* awaits future studies.

**Extended Data Fig. 9:**
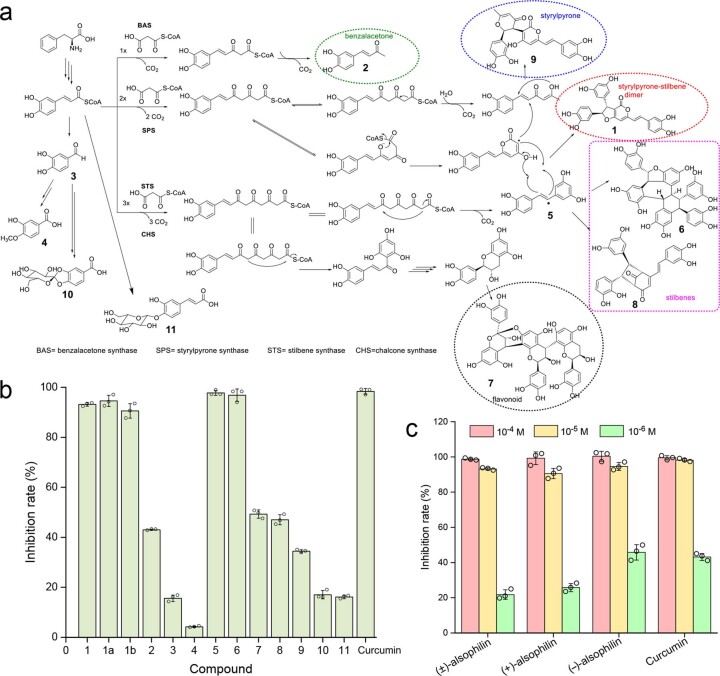
Chemical structures and antioxidant activities of 11 secondary metabolites isolated from *A. spinulosa* stems. **(a)** Chemical structure and hypothetical biosynthetic pathway of one new compound (1) and ten known compounds (2-11). compound 1: (±)-alsophilin; compound 2: 3,4-dihydroxybenzalacetone; compound 3: protocatechnic aldehyde; compound 4: vanillic acid; compound 5: piceatannol; compound 6: cyperusphenol B; compound 7: cinnamtannin B-1; compound 8: jezonodione; compound 9: davallialactone; compound 10: cyathenosin A; compound 11: 4-*O*-*β*-D-glucopyranosyl-*p*-coumaric acid. **(b)** Antioxidant effects on MDA production of pure compounds at 10^−5^ M, using curcumin as the positive control. compound 1a: (−)-alsophilin; compound 1b: (+)-alsophilin. **(c)** Antioxidant effects on MDA production of (±)-alsophilin, (−)-alsophilin, and (+)-alsophilin at 10^−4^, 10^−5^, and 10^−6^ M, respectively, using curcumin as positive control. The data of inhibition rates in **b** and **c** are presented as means ±SD of three independent experiments. Source data

To summarize, in this study we assembled the genome of *A. spinulosa* at the chromosome level. Genome analyses demonstrate that its large genome may be due to two rounds of WGD and an abundance of TEs. Synteny has been remarkably conserved despite the antiquity of WGD. Characterization of secondary metabolites identified abundant phenylpropanoid-based compounds in xylem, including lignin, alsophilin and flavonoids. Lignin is an essential component to increase the stiffness and strength of plant cell walls and provides waterproofing to the cell wall. G lignins are mainly deposited in gymnosperm tracheids for both support and transport and in angiosperm vessels for transport^31,46^. Accumulations of G lignins in tracheids of *A. spinulosa*, plus the detailed cytological observation of tracheid patterning in xylem, suggest that G lignins contribute to the function of tracheids in both support and transport. We identified two VND genes as possible key regulators for secondary wall formation and G lignin biosynthesis in tracheids, providing the molecular basis for tracheid formation in *A. spinulosa*. Alsophilin is a phenolic heterodimer of hispidin and piceatannol, and in vitro assays showed that it had antioxidant activities (Supplementary Text and Extended Data Fig. 9). We found that alsophilin, hispidin and piceatannol were primarily synthesized in xylem, of which piceatannol has been reported incorporated into the lignins in palm^47^. On the basis of our RNA-seq and recombinant protein assays, we were able to characterize some of the pathway genes leading to these metabolites. Abundant enzyme members, including PKS III, cytochrome P450 monooxygenases and oxidases (laccase and peroxidase), were identified in the *A. spinulosa* genome, which might suggest that *A. spinulosa* could be a valuable resource for natural product discovery. Lastly, demographic history inferred from genome resequencing identified two genetic bottlenecks, resulting in a rapid demographic decline of tree ferns. Together, the *A. spinulosa* genome provides a unique reference for inferring the history of genetic diversity, secondary metabolite biosynthesis and evolution of tree ferns, for better protection and application of tree ferns in the future.

## Methods

### Genome sequencing

Young leaves were collected from an *A. spinulosa* tree in the National Germplasm Resources Center (29.90° N, 103.14° E) in Hongya County, Sichuan, China. DNA was extracted using a CTAB procedure^48^. For SMRT long-read sequencing, five 20-kb DNA insert libraries were constructed using a SMRTbell Template Prep Kit (PacBio) and sequenced on a PacBio sequel I/II. For Illumina sequencing, two short-read libraries (inserts of 270 bp and 500 bp) were constructed using TruSeq DNA Sample Prep Kits (Illumina) and 150 bp pair-end sequenced on the Illumina HiSeq X-10. Five Hi-C libraries^49^ were sequenced on an Illumina Novaseq 6000 with 150-bp paired-end reads.

### Genome assembly

PacBio read self-correction was performed using Canu (v.1.9)^50^ with the following parameters: -correct; saveOverlaps, true; minMemory, 50G; batMemory, 200G; genomeSize, 7g. Corrected PacBio data were assembled into contigs using SmartDenovo (https://github.com/ruanjue/smartdenovo). Hi-C reads were aligned to the contig assembly through Juicer^51^. Contigs were mapped to pseudochromosomes using the 3D-DNA pipeline^52^. Chromosome-length scaffolds were adjusted manually with Juicebox^51^. To improve the assembly, we used BWA-MEM^53^ to map Illumina DNA reads to the genome and used Samtools^54^ to sort BAM files. The UnifiedGenotyper module of Genome Analysis Toolkit (GATK)^55^ was used to correct SNPs and indels of the long-read assembly.

Illumina genomic and RNA-seq reads were aligned to the genome using BWA-MEM^53^ and HISAT2 (ref. ^56^), respectively, to calculate mapping rate. The LTR assembly index^10^ was used to assess continuity. We also performed BUSCO^11^ evaluation to examine completeness of the assembly with the Eukaryota_odb10 database.

### RNA-seq, ISO-seq and small RNA-seq

Tissues, including leaf, stem and sorus at three developmental stages, and gametophyte cultured from spores^57^ were collected from three individual trees as biological replicates. Tissues of pith, phloem, xylem and sclerenchymatic belt in the stems were further separated. Total RNAs were extracted using CTAB^58^. RNA-seq libraries were constructed using the NEBNext Ultra RNA Library Prep Kit for Illumina (NEB) and sequenced on an Illumina HiSeq 4000 with a read length of 150 bp at both sides. DEGs were identified using DESeq2 (ref. ^59^). Quantitative PCR with reverse transcription was performed^60^ with specific primers (Supplementary Table 16). Three ISO-seq libraries of 1–2 kb, 2–3 kb and 3–6 kb were constructed using the RNAs of leaves and stems^61^ and sequenced on a PacBio Sequel. Three small RNA libraries were constructed using total RNAs from young leaves to identify microRNAs (Supplementary Text).

### Genome annotation

Tandem Repeats Finder v.4.09 (ref. ^62^) was used to scan the genome for tandem repeats with a period size >50 bp. We applied a combination of de novo and homology-based approaches at DNA and protein levels for TEs. A de novo repeat library was constructed using RepeatModeler v.2.0.1 (ref. ^63^) with a parameter of LTRStruct. RepeatMasker v.4.1.0 (ref. ^64^) was used to map our assembly against the TE sequences in the repeat library and the Repbase v.21.12 (ref. ^65^) database to classify TEs. WU-BLASTX was run against the TE protein database in RepeatProteinMask v.4.0.7 (ref. ^64^) to identify TEs at the protein level.

Annotation was conducted through homology-based, transcriptome-based and ab initio prediction methods. Homologies from six species (*A. filiculoides*^17^, *S. cucullata*^17^, *S. moellendorffii*^18^, *S. tamariscina*^66^, *Ceratopteris richardii*^67^ and *Adiantum capillus-veneris* L.) were used as protein evidence for predicted gene sets using GeneWise v.2.4.1 (ref. ^68^). Transcriptome data including RNA-seq and ISO-seq reads were mapped using HISAT2 (ref. ^56^) and minimap2 (ref. ^69^). Ab initio gene prediction was performed with AUGUSTUS, trained by the transcriptome data. The Geta pipeline (https://github.com/chenlianfu/geta) was used to integrate annotation from all homology-based, transcriptome-based and ab initio predictions to generate a comprehensive protein-coding gene set. Genes without support from hidden Markov models (HMMs), transcriptome prediction and homologous prediction were removed. Finally, a non-redundant, consensus protein-coding gene set was constructed. Additional gene functional annotation was performed by searching the NCBI nr, Swiss-Port, KOG, eggNOG^70^, InterPro, Pfam, GO and KEGG databases.

### DNA methylation

Young leaves (~500 mg) were collected from three trees for WGBS. Genomic DNA was fragmented into 300 bp, end-repaired, A-tailed and ligated to methylated adapters. DNA fragments were size-selected (350–500 bp), treated with bisulfite and amplified by PCR. After purification, three WGBS libraries were sequenced on an Illumina HiSeq X-10 with 150-bp pair-end reads, generating 833.13 Gb of clean data. Clean reads were mapped using Bismark v.16.3 (ref. ^71^) (bismark -N -1 -2 -un --bowtie2 --path_to_bowtie --bam --samtools_path -o). Telomeres and centromeres were identified^72,73^. The number of methylated cytosines (CG-type, CHG-type and CHH-type) in the genome, repeat regions and gene bodies was normalized as methylation level values.

### Phylogenetic analysis

Eleven species were selected to construct a phylogenetic tree with *A. spinulosa*: two angiosperms (*Amborella trichopoda*^74^ and *A. thaliana*), two gymnosperms (*G. biloba*^75^ and *Gnetum montanum*^76^), two ferns (*A. filiculoides* and *S. cucullata*^17^), one lycophyte (*S. tamariscina*^66^), three bryophytes (*P. patens*^77^, *Anthoceros angustus*^78^ and *Marchantia polymorpha*^79^) and one charophyte (*Chara braunii*^80^). Protein sequences were filtered by removing short sequences (less than 50 amino acids) and choosing the longest isoform to represent each protein. OrthoFinder software^81^ (parameters ‘-M msa -S diamond’) was employed to cluster gene families. Single-copy orthologues were identified and used in the phylogenetic analysis. The single-copy genes were aligned by MAFFT^82^ and trimmed by trimAl^83^, and a maximum likelihood phylogenetic tree was constructed using modeltest-ng^84^ and RAxML-ng^85^, with *C. braunii* as the outgroup. The phylogenetic tree was visualized by iTOL^86^.

To model gene family expansion and contraction across the phylogeny, we used maximum likelihood in CAFE^87^ with a cut-off *P* value of 0.05. We used r8s^88^ to obtain the ultrametric tree with the following constraints: (1) 330.9–365 Ma for seed plants^89^, (2) 197.5–246.5 Ma for angiosperms^89^, (3) 91.3–98.8 Ma for Salviniales^90^, (4) 281.4–287.5 Ma for Salviniales + Cyatheales^90^ and (5) a fixed age for land plants at 500 Ma^89^.

### Gene family analysis

We combined Hmmer and Blastp to identify gene family members. The HMM files of gene families from the Pfam protein family database were used to search genes in *A. spinulosa* using HMMER^91^. High-quality protein hits with an *e* value cut-off of 1 × 10^−20^ were aligned through MUSCLE^92^ to construct a specific HMM file for *A. spinulosa* using HMMER. This HMM file was employed to search the genome again to obtain proteins with an *e* value lower than 0.01. BLASTP was applied for the query proteins (Supplementary Table 17) to scan for homologues (*e* = 1 × 10^−10^), and RAxML^93^ was applied to construct phylogenetic trees. The candidate proteins were examined to confirm corresponding domains using Pfam, SMART and NCBI Conserved Domains databases.

### WGD

To assess the history of WGD in *A. spinulosa*, an initial Ks distribution was obtained using a whole-paranome approach where genes were first clustered, followed by pairwise comparison and Ks estimation within clusters. Whole-paranome Ks estimation and subsequent mixture modelling were performed with the WGD package using the commands ksd and mix^94^.

Synteny was assessed using MCSCANX^95^ to identify collinear blocks of gene pairs. The resulting syntenic blocks were filtered by median Ks using a Python script to select collinear gene pairs that result from a specific duplication.

To place the inferred WGD events onto a phylogeny, fern transcriptomes were selected on the basis of their phylogenetic relatedness to *A. spinulosa*. Paired-end Illumina reads were from the Sequence Read Archive for *Vandenboschia striata*, *Schizaea dichotoma*, *Azolla pinnata*, *Plagiogyria japonica*, *Dicksonia antarctica*, *Sphaeropteris lepifera*, *Gymnosphaera podopylla* and *Gymnosphaera gigantea*^96,97^. The reads were assembled using SOAPdenovo-Trans^98^, and open reading frames were identified in TransDecoder (https://github.com/TransDecoder/TransDecoder). Multiple isoforms were collapsed using CD-HIT^99^ (with a similarity threshold of 99%), followed by clustering and gene tree construction using OrthoFinder^81^ (‘-M msa’ option).

Phylogenetic assessment was conducted by gene-tree species-tree reconciliation using MAPS^100^. An initial analysis of gene trees was produced by OrthoFinder. Multiple simulations were run using the simulateGeneTrees.3.0.pl script included with MAPS. For gene tree simulation, an ultra-metric species tree containing taxa from OrthoFinder was generated using the R package ape^101^. Node ages were calibrated using maximum and minimum ages^22,102^. Next, prior estimates of background rates of gene duplication and loss were obtained using R WGDgc^103^. Finally, 1,000 trees were simulated for the following scenarios: (1) no shared WGDs (null simulation) and (2) a single WGD in both Cyatheales and Cyatheaceae (positive simulation). Following the simulation, 100 randomly resampled sets of 200 gene trees were created for each scenario and subjected to MAPS. This method artificially inflates the number of subtrees containing a WGD near the root (Z. Li, personal communication), so a separate analysis was run with a reduced subset of taxa to resolve the WGD at the base of the Cyatheales. Transcriptomic sequences from *V. striata*, *S. dichotoma*, *P. japonica*, *D. antarctica* and *A. spinulosa* were used to build gene trees in OrthoFinder and subjected to MAPS as well as the null and positive simulations. A third analysis was run using *V. striata*, *S. dichotoma*, *A. pinnata*, *P. japonica*, *D. antarctica* and *G. gigantea* in place of *A. spinulosa* to ensure that the older Cyatheales event could still be detected with altered sampling of Cyatheaceae (Supplementary Fig. 11). Final comparisons of the experimental and simulated results were assessed for significance (Fischer’s exact test in R).

Differential expression of homoeologous genes was analysed using the RNA-seq data from four tissues: stem, leaf, sorus and gametophyte (Supplementary Text).

### Substitution rate

Substitution rates in Cyatheales were evaluated using protein-coding genes from *A. spinulosa* and transcriptomes of other Cyatheales genera, six representatives from the remaining leptosporangiate orders, and one from the eusporangiate order Marattiales^104,105^. The transcriptomes were assembled by Trinity^106^, and redundant sequences were removed by CD-HIT^99^. OrthoFinder^81^ was used to identify orthogroups. In each inferred orthogroup, we removed taxa with more than one sequence, probably due to gene duplication. We only analysed orthogroups that contained sequences longer than 300 bp and covered more than 75% of the taxon sampling. Each orthogroup was aligned on the basis of amino acid sequences using MAFFT^82^ (‘--maxiterate 16 --globalpair’). PAML^107^ was then used to detect substitution rate changes in Cyatheales. The input topology for baseml analyses was derived from PPG 1 (ref. ^3^). The significance of a rate change was inferred by a likelihood ratio test between two baseml models. One was set under a global clock with one rate, and another was under a local clock with two rates in which Cyatheales was set to have a different rate.

### Light microscopic imaging

Four tissues (pith, sclerenchymatic belt, phloem and xylem) were separated from fresh stems and cut into pieces 1.5–2 cm long. The materials were boiled in water (20 min) and then soaked in 10% nitric acid and 10% chromic acid (v/v = 1:1) for 16 h to dissociate the cells. The mix was filtered through 200-mesh nylon and washed twice with dH_2_O. The materials were pounded by a glass rod and stored in 50% ethanol. The material was stained with 1% safranine for 2 min, washed with dH_2_O and observed under a light microscope (Olympus BX51).

### SEM, TEM and microCT imaging

Fresh *A. spinulosa* stems were cut to proper size and fixed in 0.1 M phosphate buffer (pH 7.4) containing 4% (v/v) glutaraldehyde for 4 h at room temperature. The samples were washed three times with 0.1 M phosphate buffer and post-fixed with 2% osmium tetroxide (w/v) plus 1.5% potassium ferricyanide (w/v) in phosphate buffer for 2 h at 4 °C. Following three rounds of water washing, in-bloc staining with 2% uranyl acetate (w/v) was performed overnight at 4 °C. The samples were dehydrated through a graded ethanol series.

For SEM observation, the samples were dried in a critical point dryer (CPD300, Leica) and imaged in a ThermoFisher Quanta 450. For TEM observation, the samples were embedded in fresh resin and polymerized at 65 °C for 24 h. Sections (70 nm) were made using a Leica UC7 ultramicrotome and post-stained with uranyl acetate and lead citrate. Grids were imaged at 80 kV in a JEOL Jem-1400 TEM using a CMOS camera (XAROSA, EMSIS). The polymerized resin block was also used for microCT (SkyScan 1272, Bruker) imaging, and the microCT data were processed using Amira (v.2020.3) software.

### Lignin content and composition determination

Samples were ground, lyophilized and extracted successively with chloroform/methanol (2:1, v/v), methanol and water at room temperature to remove extractives. The remaining cell wall residues were again lyophilized. Lignin content was determined by Klason^108^, and monolignol composition was determined by thioacidolysis^109^. Lignin structures were analysed by NMR^110,111^.

### Metabolite characterizations and biological activity assays

Leaves and stems at three developmental stages were collected from three individual trees for a metabolomic screen^41^. Stem powders were extracted with a series of solvents, followed by column chromatography. Eleven purified metabolites were tested for antioxidant activities, in vitro cytotoxicity and anti-inflammation. The details of the metabolite characterization and biological activity assays are described in the Supplementary Text.

### Quantification of alsophilin, hispidin, resveratrol and piceatannol

Air-dried powders (60 mg, <60 mesh) of leaves, xylem, phloem, sclerenchymatic belt and pith were extracted with 400 µl of methanol by ultrasonication for 15 min. After filtering (0.22 µm), 10 µl of filtrate was analysed by UPLC (Waters) and MS (Thermo-Fisher) on an ACQUITY UPLC column (2.1 mm × 50 mm, C18) with a flow rate of 0.4 ml min^−1^ and a gradient of solvent A (acetonitrile) and solvent B (H_2_O). Alsophilin was detected under a t-SIM model (gradient: 0 min, 10% A; 6 min, 90% A; 7 min, 10% A; 9 min, 10% A; selected positive ion at *m*/*z* 163.0386). Hispidin, resveratrol and piceatannol were detected under a PRM model (gradient: 0 min, 10% A; 7 min, 90% A; 9 min, 10% A; selected negative ion at *m*/*z* 159.0440, 185.0598 and 159.0440, respectively).

### Enzyme assays

The full-length coding regions of PKS genes were cloned into pGEXKG-1 for protein expression in *E. coli* BL21 (DE3). The primers are shown in Supplementary Table 16. The enzyme assays were performed in a 100 μl volume containing 1 μl of 10 mM *p*-coumaroyl-CoA or caffeoyl-CoA, 3 μl of 10 mM malonyl-CoA, 90 μl of 50 mM Tris-HCl buffer (pH 7.5) and purified PKS enzymes at a final concentration of 1 mg ml^−1^. The reactions were incubated overnight at 30 °C or 37 °C and stopped by the addition of methanol to 50%. The products were analysed by LC–MS on a LCMS-2020 (Shimadzu) with a Shim-pack GIST column (5 μm, 2.1 mm × 100 mm) monitor at 310 nm and 30 °C, in negative ionization mode with a full scan range of 100–500 *m*/*z*. The mobile phases were solvent A (water) and solvent B (methanol), with a flow rate of 0.3 ml min^−1^ and a gradient of 0 min, 10% B; 10 min, 30% B; 20 min, 60% B; 30 min, 100% B.

### Resequencing and population analysis

#### Read mapping and variant calling

We collected leaves from 107 *A. spinulosa* trees from nine populations in Southeast Asia, with *A. costularis* as the outgroup. DNA libraries with 200–400-bp inserts were constructed and pair-end sequenced on MGISEQ2000. After quality control by FastQC, the raw reads were filtered to remove adaptors, contaminants and low-quality reads using Trimmomatic^112^. We generated 8,755.59 Gb of sequence, with an average depth of 13.2× genome coverage per accession. The clean reads were mapped using Bowtie2 (ref. ^113^) with the default parameters. SAMtools^54^ was used to remove duplicate reads. We evaluated the rate of uniquely aligned reads that were obtained from BWA^114^. We used Realigner Target Creator and Indel Realigner from the GATK package^55^ for global realignment of reads around indels from the sorted BAM files. HaplotypeCaller was used to estimate the SNPs and indels for putative diploids using the default parameters. The distribution of calling depths (DP) of each raw variant was estimated as a criterion for variant filtering to reduce false positives. Low depths and repetitive variants were removed from the raw VCF file if they had DP < 2 or DP > 45, minQ < 30. Variants with more than 15% missing data were removed. These filtering strategies reduced the raw unfiltered set of 160,416,579 variants (SNPs and indels) to a working set of 93.86 million. SnpEff (v.3.6c)^115^ was used to assign variants on the basis of gene models from *A. spinulosa* annotation. The variant sites were annotated as SNPs and indels, as well as intergenic and genic regions (including synonymous, non-synonymous, intronic, upstream and downstream variants).

#### Genome-wide genetic diversity estimation

To identify selective sweeps, we calculated the genome-wide distribution of Tajima’s *D* and nucleotide diversity θπ values using VCFtools^116^ with a 20-kb sliding window. SweeD^117^ analysis was conducted on the basis of the CLR to identify selected loci, and the CLR of each sliding window with a size of 20 kb was calculated. Both CLR and θπ analysis used the top 5% scoring regions. Tajima’s *D* used the top and bottom 2.5% scoring regions as cut-off values to infer candidate selective sweeps. Regions that were supported by both approaches were considered high-confidence.

#### Phylogeny

Bi-allelic and polymorphic SNPs (58,177,625) were used to reconstruct phylogenetic relationships among the 107 accessions. Before tree construction, we filtered and pruned the SNPs with minor allele frequency < 0.2, missing rate > 0.15 and linkage disequilibrium threshold = 0.2. Finally, 263,712 SNPs located in single-copy genes were selected for constructing the tree. The multiple consensus sequences were aligned using MAFFT^82^. Maximum likelihood trees were constructed using RAxML^93^. iTOL^86^ was used to visualize the tree.

#### PCA

The GCTA software^118^ was employed to conduct PCA on 263,712 filtered variants. The input PLINK binary files were transformed from the filtered VCFs file using VCFtools^116^ and PLINK^119^. The top three principal components were used for assigning the 107 accessions and downstream population structure.

#### Population genetic structure

We used Admixture^120^ to infer ancestral population stratification among the 107 accessions. The optimal ancestral population structure was estimated from the same variant set with STRUCTURE^121^ using ancestral population sizes *K* = 2–9 and choosing the population with the lowest cross-validation error. The standard errors were estimated using bootstrapping (100 replicates) during the admixture analyses.

#### Demographic analysis

The demographic history was queried on the basis of the site-frequency spectrum (SFS) inferred from alignment of population resequencing. Low-quality mapping was first removed with the parameters (‘-only_proper_pairs 1 -uniqueOnly 1 -remove_bads 1 -minQ 20 -minMap 30’) implemented in ANGSD^122^. The site allele-frequency likelihood was calculated using -doSaf for each resequenced accession on the basis of individual genotype likelihoods. We used the realSFS with the expectation-maximization algorithm to calculate the folded SFS on the basis of the estimation of maximum likelihood. After that, stairway-plot-2 (ref. ^123^) was used to present the historical effective population size (*N*_e_) with an estimated mutation rate of 1.77 × 10^−9^ per generation and a 100-year generation time, derived from previous studies^124–126^.

### Reporting Summary

Further information on research design is available in the Nature Research Reporting Summary linked to this article.

### Supplementary information

Supplementary Information Supplementary Text, Figs. 1–12 and Tables 1–22.

Reporting Summary

Supplementary Data Supplementary Data 1–10 and statistical source data for Supplementary Figs. 2b,c, 7b, 8e, 11 and 12a,c.

### Source data

Source Data Fig. 1 Statistical source data for Fig. 1b,d.

Source Data Fig. 2 Statistical source data for Fig. 2g.

Source Data Fig. 3 Statistical source data for Fig. 3b–d.

Source Data Extended Data Fig. 3 Statistical source data for Extended Data Fig. 3a–d.

Source Data Extended Data Fig. 4 Statistical source data for Extended Data Fig. 4b.

Source Data Extended Data Fig. 5 Statistical source data for Extended Data Fig. 5a,b.

Source Data Extended Data Fig. 9 Statistical source data for Extended Data Fig. 9b,c.

## Data Availability

The genome assemblies of *A. spinulosa* have been deposited to the Genome Sequence Archive at the National Genomics Center under BioProject no. PRJCA006485. Whole-genome sequencing, RNA-seq, resequencing, WGBS and small RNA sequencing data were deposited to the GSA database (http://gsa.big.ac.cn/) under accessions CRA005445, CRA005406, CRA005447, CRA005463, CRA005407 and CRA005430. The genome assembly and annotation files are available at Figshare (10.6084/m9.figshare.19075346), and all phylogenetic trees in newick formats and with bootstrap values are deposited in Figshare (10.6084/m9.figshare.19125641). Source data are provided with this paper.

## Code availability

All custom codes are available for research purposes from the corresponding authors upon request.
