# Author Correction: The flying spider-monkey tree fern genome provides insights into fern evolution and arborescence

**DOI:** 10.1038/s41477-024-01631-0

**Published:** 2024-02-02

**Authors:** Xiong Huang, Wenling Wang, Ting Gong, David Wickell, Li-Yaung Kuo, Xingtan Zhang, Jialong Wen, Hoon Kim, Fachuang Lu, Hansheng Zhao, Song Chen, Hui Li, Wenqi Wu, Changjiang Yu, Su Chen, Wei Fan, Shuai Chen, Xiuqi Bao, Li Li, Dan Zhang, Longyu Jiang, Dipak Khadka, Xiaojing Yan, Zhenyang Liao, Gongke Zhou, Yalong Guo, John Ralph, Ronald R. Sederoff, Hairong Wei, Ping Zhu, Fay-Wei Li, Ray Ming, Quanzi Li

**Affiliations:** 1 State Key Laboratory of Tree Genetics and Breeding, Chinese Academy of Forestry, Beijing, China; 2 Shenzhen Branch, Guangdong Laboratory for Lingnan Modern Agriculture, Genome Analysis Laboratory of the Ministry of Agriculture and Rural Affairs, Agricultural Genomics Institute at Shenzhen, Chinese Academy of Agricultural Sciences, Shenzhen, China; 3 State Key Laboratory of Bioactive Substance and Function of Natural Medicines; NHC Key Laboratory of Biosynthesis of Natural Products; CAMS Key Laboratory of Enzyme and Biocatalysis of Natural Drugs, Institute of Materia Medica, Chinese Academy of Medical Sciences & Peking Union Medical College, Beijing, China; 4 Thompson Institute, Ithaca, NY USA; 5 Plant Biology Section, Cornell University, Ithaca, NY USA; 6 Institute of Molecular & Cellular Biology, National Tsing Hua University, Hsinchu, Taiwan; 7 Beijing Key Laboratory of Lignocellulosic Chemistry, Beijing Forestry University, Beijing, China; 8 Department of Biochemistry and DOE Great Lakes Bioenergy Research Center, Wisconsin Energy Institute, University of Wisconsin, Madison, WI USA; 9 State Forestry Administration Key Open Laboratory on the Science and Technology of Bamboo and Rattan, Institute of Gene for Bamboo and Rattan Resources, International Center for Bamboo and Rattan, Beijing, China; 10 State Key Laboratory of Tree Genetics and Breeding, Northeast Forestry University, Harbin, China; 11 Beijing Advanced Innovation Center for Tree Breeding by Molecular Design, Beijing Forestry University, Beijing, China; 12 College of Landscape Architecture and Forestry, Qingdao Agricultural University, Qingdao, China; 13 GoldenGate International College, Tribhuvan University, Battisputali, Kathmandu, Nepal; 14 State Key Laboratory of Systematic and Evolutionary Botany, Institute of Botany, Chinese Academy of Science, Beijing, China; 15 Forest Biotechnology Group, Department of Forestry and Environmental Resources, North Carolina State University, Raleigh, NC USA; 16 College of Forest Resources and Environmental Science, Michigan Technological University, Houghton, MI USA; 17 Department of Plant Biology, University of Illinois at Urbana-Champaign, Urbana, IL USA

**Keywords:** Plant morphogenesis, Secondary metabolism, Phylogenetics, Genome evolution, Genetic variation

Correction to: *Nature Plants* 10.1038/s41477-022-01146-6, published online 9 May 2022.

In the version of the article initially published, Dipak Khadka, who collected the samples in Nepal, was thanked in the Acknowledgements instead of being listed as an author. His name and affiliation (GoldenGate International College, Tribhuvan University, Battisputali, Kathmandu, Nepal) have been added to the authorship in the HTML and PDF versions of the article.

