# Supplementary material for: The flying spider-monkey tree fern genome provides insights into fern evolution and arborescence
Source: Nat Plants. 2022 May 9;8(5):500–12. doi: 10.1038/s41477-022-01146-6 (PMC9122828; doi:10.1038/s41477-022-01146-6)
Supplement: Supplementary file 1 — Supplementary Text, Figs. 1–12 and Tables 1–22. [file 41477_2022_1146_MOESM1_ESM.pdf]

nature portfolio

https://doi.org/10.1038/s41477-022-01146-6

Supplementary information

The flying spider-monkey tree fern genome provides insights into fern evolution and arborescence

In the format provided by the authors and unedited

Supplementary Information

The flying spider-monkey tree fern genome provides insights into fern evolution and arborescence

Supplementary text

Small RNA sequencing

Three small RNA libraries were constructed using total RNAs isolated from young leaves as described before[1]. Libraries were sequenced on a Novaseq 6000 platform with a single-end read length of 50 bp. Clean reads were mapped to the *A. spinulosa* genome by Bowtie[2], and known miRNAs were identified using miRBase 22.0 database with miRDeep2[3]. Novel miRNAs were predicted by the combination of miREvo[4] and miRDeep2[3].

We totally obtained 29.64 million clean reads of sRNA with length at 18-30 nt. Of which, 21 nt length of sRNA were the most abundant, occupying 34.72% (Supplementary Fig. 12). 9,561,080 (96.78%) reads could be mapped to the *A. spinulosa* genome. A total of 182 known and 181 novel miRNAs were identified (Supplementary Table 18) and their first nucleotides mainly trended to be uracil (Supplementary Fig. 12). The rRNA, tRNA, snRNA and snoRNA were predicted, and the percentage of rRNA was dominant, with 93.11% (Supplementary Table 19 and Supplementary Fig. 12). Three leaf samples shared a high correlation coefficient of miRNA TPM values ($R^2 > 0.97$), indicating a quite good repeatability (Supplementary Fig. 12).

Differential expression of homeologous genes and GO enrichment analysis

Raw RNA-seq read counts from four tissues (stem, leaf, sorus and gametophyte) were normalized by DESeq2[5]. Log2 fold change (L2F) and base mean expression (BME) level were calculated for each gene across the 4 tissue types using DESeq2[5].

Following normalization and differential expression analysis, differential expression data was extracted for syntenic gene pairs identified as belonging to the most recent WGD (Ks=0.2-0.5). Subsequently, BME and L2F in expression between tissue types were used to identify homoeologs that had diverged in expression following duplication. To identify gene pairs with divergent expression between tissue types we calculated the pairwise difference in L2F (diffL2F=|L2F_1-L2F_2|) between

homoeologs and selected pairs with a difference of at least 2, an arbitrary cutoff representing a 4 fold change in expression between the syntenic genes. To identify homoeologs with different levels of expression regardless of tissue type, pairs were filtered according to the log2 fold change in base mean expression across all tissue types, i.e. "BME ratio" (BME_ratio=log2(BME_1/BME_2)). Finally, both BME_ratio as well as diffL2F were compared between collinear blocks to look for evidence of expression bias between homologous sub genomes.

Out of 3,921 syntenic gene pairs with Ks between 0.2 and 0.5, 3,557 had at least one gene with a BME greater than 10 and were included in subsequent analyses. After filtering, 1,184 gene pairs showed a BME ratio greater than 2, indicative of differential expression regardless of tissue type and 141 pairs had one gene with a BME below 1 indicative of one homolog being silenced in all 4 tissues. A total of 2,105 gene pairs exhibited differential expression in at least one comparison, and many pairs were differentially expressed in multiple comparisons with 136 gene pairs differentially expressed in all comparisons (Extended Data Fig. 3).

Datasets were constructed for GO enrichment using a custom Python script to filter the output of DESeq2 by difference in L2F expression between tissues as well as BME ratio. Pairwise tissue comparisons were collapsed to produce a non-redundant set of differentially expressed genes across all tissue types. Functional gene annotations were obtained using the EggNOG-mapper tool[6]. GO-term enrichment was conducted in TBtools[7] using the plant specific GO-slim ontology. GO-enrichment was first performed using the complete set of annotated genes as a background set. An additional analysis was also run using only syntenic genes in the background set to control for bias toward specific categories caused by differential retention of certain types of genes following WGD.

To examine which genes are preferentially kept following WGD, we carried out Gene Ontology (GO) enrichment analysis. When compared to a genome-wide background annotation, syntenic gene pairs from the recent WGD event ($0.2<Ks<0.5$) exhibited significant enrichment of transcription related genes in the molecular function category as well as various categories associated with development and

response to a range of environmental stimuli (Extended Data Fig. 3). This is in line with previous studies that have shown high retention rates of homoeologous genes in these GO categories following WGD events in *Arabidopsis thaliana*[8].

When GO enrichment analysis was conducted on differentially expressed gene pairs using all syntenic genes as the background, we found that genes exhibiting tissue specific expression were highly enriched for transporter activity (p-value=0.0023) compared to other syntenic gene pairs (Extended Data Fig. 3).

GO enrichment analysis demonstrated that homoeologous genes with divergent expression were enriched in different GO categories than genes that did not exhibit tissue specific expression.

Metabolite identification based on widely targeted metabolomics

We exploited the technology of widely targeted metabolomics[9] from Metware Company (Wuhan, China) to investigate the metabolites in the leaves and stems of *A. spinulosa*. Leaves and stems at three developmental stages, collected from three individual trees, were freeze-dried and crushed using a mixer mill. Powder (100 mg) was extracted overnight at 4 °C with 70% methanol. The extracts were absorbed into a CNWBOND Carbon-GCB SPE cartridge (ANPEL), filtered through a 0.22 μm nylon syringe filter and analyzed using an LC-ESI-MS/MS system (HPLC, Shim-pack UFLC SHIMADZU CBM30A system; MS, Applied Biosystems 6500 Q TRAP). We conducted OPLS-DA (Orthogonality Partial Least Squares-Discriminant Analysis)[10] to obtain VIP (variable importance in project) values. All compounds were annotated in the KEGG[11] database and a self-built MetWare[12] database. The metabolites that have significantly differential contents in different tissues were identified using two cutoffs (VIP ≥1; FC (Fold Change) ≥2 or FC ≤0.5).

A total of 274 metabolites were identified, including 87 primary metabolites and 187 secondary metabolites (Supplementary Fig. 8 and Supplementary Dataset 8). Secondary metabolites include 72 flavonoids, 29 phenylpropanoids and 11 alkaloids, etc, of which flavonoids were the most abundant, occupying 38.5% of secondary metabolites (Supplementary Fig. 8 and Supplementary Dataset 8). Some flavonoids

have application potential in pharmacology: for examples, quercetin could enhance antitumor activities of trichostatin A[13], and eriodictyol had anti-inflammatory effects[14]. Most flavonoid compounds were detected at higher-level contents in leaves than in stems (Supplementary Fig. 8). Further by comparison of flavonoid contents in leaves with different developmental stages, 49 were significantly upregulated in old leaves compared to young leaves, such as naringenin (2.2 folds), eriodictyol (6.0 folds), apigenin 7-*O*-glucoside (49.1 folds) and kaempferol 3-*O*-galactoside (57.8 folds), indicating content accumulation of major flavonoids along with leaf development (Supplementary Fig. 8 and Supplementary Dataset 9). RNA-seq analysis of the 358 gene models (Supplementary Dataset 10) in the 8 family (CHS, CHI, F3H, FLS, F3′H, F3′5′H, FNS and GT) of flavonoid pathway identified the genes that were specifically expressed in different tissues, supporting the flavonoid synthesis in leaves and stems (Supplementary Fig. 8).

Metabolite identification based on isolation and spectra

Six kg of dried stem powder was extracted with ethanol to obtain a crude extract, which was further extracted successively in petroleum ether, ethyl acetate and *n*-butanol, followed by condensation, obtaining a petroleum ether extract (Fraction A, Fr. A), an ethyl acetate extract (Fraction B, Fr. B), and an *n*-butanol extract (Fraction C, Fr. C). We used different columns to separate these three fractions: a silica-gel column (100-200 and 200-300 mesh, Qingdao Marine Chemical) for Fr. A, an ODS-C18 column (50 μm, YMC) for Fr. B, and a MCI HP20 column (Mitsubishi) for Fr. C. After elution with different solvents, i.e. CH_2Cl_2-MeOH (100:0→0:100, v/v) for Fr. A, MeOH (30%-100%) for Fr. B, and EtOH (30%-100%) for Fr.C), we obtained 40 fractions (Fr. A1-30, Fr. B1-6 and Fr. C1-C4). Fr. A8, Fr. B1 and Fr. B2 were further separated by the Sephadex LH-20 column (18-110 μm, GE healthcare) and eluted with MeOH, giving subfractions Fr. A8.1-A8.13, Fr. B1.1-B1.25, and Fr. B2.1-B2.30.

Eleven metabolites were purified from these fractions by semi-preparative HPLC on an Agilent C18 column (250 × 10 mm I.D., 5 μm) equipped with a LC-6AD pump

and a Shimadzu SPD-20A UV-Vis detector (Shimadzu) using MeOH-H_2O as eluent. The structures of one novel metabolite alsophilin and ten metabolites were identified by MS and NMR spectra (Supplementary Fig.9). The optical rotations were recorded using a Rudolph Research Analytical automatic polarimeter (Rudolph). Its molecular formula was identified by high-resolution electrospray ionization mass spectrometry. UV spectrum was measured on a JASCO J-815 CD spectropolarimeter (JASCO). IR spectrum was acquired by KBr disk method on a Shimadzu FTIR-8400S spectrometer (Shimadzu). HPLC, equipped with a chiral column (Daicel Chiralpak IC) and using isopropanol and hexane (60:40) as eluent, was used to separate (−)-alsophilin and (+)-alsophilin from alsophilin. The absolute configurations of (±)-alsophilin were assigned by experimental and theoretical electronic circular dichroism (ECD)[15]. A conformational search was conducted using the MOE software with the MMFF94s force-field. All 34 conformers whose Boltzmann distribution was more than 1% were optimized at the B3LYP/6-311G (d,p) level in MeOH using a PCM solvent model with the Gaussian 16 program package. ECD calculations of all the conformers were run by the time-dependent density functional theory (TDDFT) at B3LYP/6-311g (d,p) level in MeOH with the PCM solvent model (Supplementary Table 20). The overall calculated ECD spectra were generated by Boltzmann weighting using SpecDis. Through isolation and identification of metabolites in the *A. spinulosa* stem, we obtained eleven compounds, including ten known and one novel. Of which, ten known compounds were identified by comparing their spectroscopic data with the reported data in the corresponding literatures as 3,4-dihydroxybenzalacetone (**2**)[16,17], protocatechnic aldehyde (**3**)[17], vanillic acid (**4**)[16], piceatannol (**5**)[18,19], cyperusphenol B (**6**)[19], cinnamtannin B-1 (**7**)[20], jezonodione (**8**)[18,20], davallialactone (**9**)[21], cyathenosin A (**10**)[22], 4-*O*-*β*-D-glucopyranosyl-*p*-coumaric acid (**11**)[23] (Extended Data Fig. 9).

Characterization of alsophilin structure

The novel compound, mentioned above, was named alsophilin. It was obtained as yellow amorphous powder with the molecular formula $C_{27}H_{20}O_9$ identified by high-resolution electrospray ionization mass spectrometry (HRESIMS,

Supplementary Fig. 9) at *m/z* 489.1179 ($[M+H]^+$, calcd for $C_{27}H_{21}O_9$, 489.1180), requiring 18 degrees of unsaturation. UV and IR spectrum of (±)-alsophilin are shown in Supplementary Fig. 9. The ^1H NMR spectrum (Supplementary Table 21 and Supplementary Fig. 9) exhibited the signals assignable to two 1,2,4-trisubstituted benzenes at δ 7.06 (d, *J* = 2.0 Hz), 6.97 (dd, *J* = 8.5, 2.0 Hz), 6.78 (d, *J* = 8.5 Hz), and 6.76(d, *J* = 2.0 Hz), 6.66 (dd, *J* = 8.5, 2.0 Hz), 6.79 (d, *J* = 8.5 Hz), a 1,3,5-trisubstituted benzene at δ 6.12(2H, d, *J* = 2.5 Hz), 6.18 (t, *J* = 2.5 Hz), a *trans*-1,2-disubstituted double bonds at δ 7.38 (d, *J* = 16.0 Hz) and 6.70 (d, *J* = 16.0 Hz), an olefinic methine singlet proton attributable to δ 6.43, two aliphatic methine doublet protons at δ 5.43(d, *J* = 6.5 Hz) and 4.26 (d, *J* = 6.5 Hz). In the ^{13}C NMR spectrum, 27 carbons were observed (Supplementary Table 21 and Supplementary Fig. 9), including one α, β-unsaturated ester carbon (δ163.1), eight oxygen-bearing aromatic or olefinic quaternary carbons (δ173.8, 164.9, 160.1 (2C), 148.9, 147.3, 146.9, 146.8), four carbon-substituted aromatic or olefinic quaternary carbons (δ 144.7, 132.5, 128.7, 103.8), 12 aromatic or olefinic methines (δ138.1, 122.2, 119.0, 117.0, 116.6, 116.4, 114.9, 113.8, 106.7(2C), 102.6, 96.3), and two aliphatic methines (δ 97.8, 55.0) according to its ^{13}C NMR and HSQC spectra (Supplementary Fig. 9). Above spectral data implied that (±)-alsophilin was a dimeric structure of hispidin[24] and piceatannol[25]. To confirm the structure and determine the connection mode of two monomer units, HMBC spectrum was examined (Supplementary Fig. 9). The long-range correlations from H-2 to C-4, C-6 and C-7, from H-5 to C-1 and C-3, from H-6 to C-2, C-4 and C-7, from H-7 to C-2, C-6 and C-9, from H-8 to C-1, C-9 and C-10, and from H-10 to C-8, C-11, C-12 and C-13 assigned the hispidin moiety. The key HMBC correlations from H-2′ to C-4′, C-6′ and C-7′, from H-5′ to C-1′ and C-3′, from H-6′ to C-2′ and C-4′, and from H-10′(14′) to C-8′ and C-12′ confirmed the rings A and B of piceatannol. Other long-range correlations from H-7′ to C-11, C-1′, C-2′, C-6′, and C-9′, from H-8′ to C-11, C-12, C-13, C-1′, C-9′, and C-10′(14′), indicated the central position of C-7′ and C-8′, that were oxidized from the olefinic carbons in piceatannol moiety. Both of them connected the monomer units of rings A and B of piceatannol and ring C of hispidin. Thus, all protons and carbon resonances were

assigned and the planar structure of (±)-alsophilin was established.

The relative configuration of (±)-alsophilin is determined to be *trans* orientation between protons P7′ and P8′ from the key ROESY correlations between protons P7′/P10′(P14′) and P8′/P2′ observed in the ROESY spectrum (Supplementary Fig. 9). Next, the specific optical rotation and CD spectrum of alsophilin were examined. Though the CD spectrum showed cotton curves (Extended Data Fig. 6) but the rotatory value was nearly zero, suggesting (±)-alsophilin may be a pair of enantiomers with different amounts. The enantiomers, (+)-alsophilin and (−)-alsophilin were separated by Agilent 1260 (Agilent Technologies, USA) on a chiral column Daicel Chiralpak IC column (250 × 4.6 mm I.D., 5 μm, Daicel Chemical Industries, Ltd., Japan). When we analyzed (±)-alsophilin, (+)-alsophilin and (−)-alsophilin on HPLC (CAPCELL PAK ADME column (150 × 4.6 mm, 5 μm); Mobile phase: 0min, 10% - 30min, 50% - 35min, 100% ACN-H_2O; Wavelength: 382 nm; Flow rate: 1.0 mL/min), they had the same retention time at 20.835 min. When we analyzed (±)-alsophilin, (+)-alsophilin and (−)-alsophilin on HPLC (Column: Daicel Chiralpak IC column (250 × 4.6 mm, 5 μm); Mobile phase: hexane-isopropanol (6:4); Wavelength: 382 nm; Flow rate: 1.0 mL/min), they had different retention time, displaying (+)-alsophilin (7.895 min), (−)-alsophilin (15.037 min) (Extended Data Fig. 6). The rotatory values of these two monomers were the inverse of each other, (−)-alsophilin: $[\alpha]_D^{20}$ −13.5 (c 0.2, MeOH); CD (c 0.1(w/v)%, MeOH) 211 (23.85), 245 (12.94), 285 (4.50), 380 (−3.72) nm. (+)-alsophilin: $[\alpha]_D^{20}$ 8.5 (c 0.13, MeOH); CD (c 0.1(w/v)%, MeOH) 212 (−19.30), 245 (−10.44), 283 (−4.58), 386 (2.67) nm, and the CD spectra showed the mirror-image cotton effects, further confirming that (±)-alsophilin were a pair of enantiomers (Extended Data Fig. 6). The above results indicated that the product with CD effects is not the optical monomer and is the enantiomer in the different ratio. Quantum chemical calculations of the electronic circular dichroism (ECD) spectra confirmed the absolute configurations of (−)-alsophilin and (+)-alsophilin as 7'S,8'S and 7'R,8'R, respectively by comparing experimental and predicted ECD curves (Extended Data Fig. 6).

Biological activity assays of 11 metabolites

These 11 metabolites were tested for their antioxidant efficacies, anti-inflammatory effects, and cytotoxicities. Antioxidant activities were evaluated by measuring lipid

peroxidation product malondialdehyde (MDA) as described[26]. The effect of the compounds on the production of MDA was calculated according to the OD at 532 nm. *In vitro* cytotoxicities (represented by IC_{50} values) were evaluated against five tumor cell lines, including human cerebroma (U251), human hepatoma cells (HepG2), human gastric cancer cells (HGC27), human breast carcinoma cells (MCF7), and human colorectal carcinoma cells (HCT-116), using the MTT method based on the reported procedure[27]. Anti-inflammation activities were evaluated based on nitric oxide (NO) product inhibition in lipopolysaccharide (LPS)-stimulated RAW264.7 cells as described[28]. NO production was determined by the Griess reaction and measured at 540 nm.

Significant antioxidant effects were detected for compounds (±)-alsophilin, (−)-alsophilin, (+)-alsophilin, piceatannol and cyperusphenol B, and their inhibitory rates on the lipid peroxidation product malondialdehyde (MDA) were all above 90% at 10^{-5} M (Extended Data Fig. 9 and Supplementary Table 22). None of them showed any anti-inflammatory or cytotoxic activity.

References

1 Li, H. et al. MicroRNA comparison between poplar and larch provides insight into the different mechanism of wood formation. *Plant Cell Rep.* **39**, 1199-1217 (2020).

2 Langmead, B. & Salzberg, S. L. Fast gapped-read alignment with Bowtie 2. *Nat. Methods* **9**, 357-359 (2012).

3 Friedlnder, M. R., Mackowiak, S. D., Li, N., Chen, W. & Rajewsky, N. miRDeep2 accurately identifies known and hundreds of novel microRNA genes in seven animal clades. *Nucleic Acids Res.* **40**, 37-52 (2012).

4 Wen, M., Shen, Y., Shi, S. & Tang, T. miREvo: an integrative microRNA evolutionary analysis platform for next-generation sequencing experiments. *BMC Bioinformatics* **13** (2012).

5 Love, M. I., Huber, W. & Anders, S. Moderated estimation of fold change and dispersion for RNA-seq data with DESeq2. *Genome Biol.* **15**, 550 (2014).

6 Huerta-Cepas, J. et al. Fast genome-wide functional annotation through orthology assignment by eggNOG-mapper. *Mol. Biol. Evol.* **34**, 2115-2122 (2017).

7 Chen, C., Chen, H., Zhang, Y., Thomas, H. R. & Xia, R. TBtools: an integrative toolkit developed for interactive analyses of big biological data.

Mol. Plant **13**, 1194-1202 (2020).
8 Maere, S. et al. Modeling gene and genome duplications in eukaryotes. *Proc. Natl. Acad. Sci. USA* **102**, 5454-5459 (2005).
9 Chen, W. et al. Genome-wide association analyses provide genetic and biochemical insights into natural variation in rice metabolism. *Nat. Genet.* **46**, 714-721 (2014).
10 Thévenot, E., Roux, A., Xu, Y., Ezan, E. & Junot, C. Analysis of the human adult urinary metabolome variations with age, body mass index, and gender by implementing a comprehensive workflow for univariate and OPLS statistical analyses. *J. Proteome Res.* **14**, 3322-3335 (2015).
11 Gerlich, M. & Neumann, S. KEGG: Kyoto encyclopedia of genes and genomes. *Nucleic Acids Res.* **28**, 27-30 (2000).
12 Long, L., Liu, J., Gao, Y., Xu, F. C. & Gao, W. Flavonoid accumulation in spontaneous cotton mutant results in red coloration and enhanced disease resistance. *Plant Physiol. Biochem.* **143**, 40-49 (2019).
13 Chuang, C-H., Chan, S-T., Chen, C-H. & Yeh, S-L. Quercetin enhances the antitumor activity of trichostatin A through up-regulation of p300 protein expression in p53 null cancer cells. *Chem. Biol. Interact.* **306**, 54-61 (2019).
14 Mokdad-Bzeouich, I. et al. Investigation of immunomodulatory and anti-inflammatory effects of eriodictyol through its cellular anti-oxidant activity. *Cell Stress Chaperones* **21**, 773-81 (2016).
15 Warnke, I. & Furche, F. Circular dichroism: electronic. *Wiley Interdisciplinary Reviews Computational Molecular Science* **2**, 150-166 (2012).
16 Liu, C. et al. Chemical Constituents from *Inonotus obliquus* and their biological activities. *J. Nat. Prod.* **77**, 35-41 (2014).
17 Seulah, L. et al. Anti-Inflammatory phenolic metabolites from the edible fungus *Phellinus baumii* in LPS-Stimulated RAW264.7 cells. *Molecules* **22**, 1583 (2017).
18 Kiselev, K. V., Grigorchuk, V. P., Ogneva, Z. V., Suprun, A. R. & Dubrovina, A. S. Stilbene biosynthesis in the needles of spruce *Picea jezoensis*. *Phytochemistry* **131**, 57-67 (2016).
19 Ito, T. et al. Occurrence of stilbene oligomers in *Cyperus* rhizomes. *Fitoterapia* **83**, 1420-9 (2012).
20 Idowu, T. O. et al. Doubly linked, A-type proanthocyanidin trimer and other constituents of *Ixora coccinea* leaves and their antioxidant and antibacterial properties. *Phytochemistry* **71**, 2092-8 (2010).
21 Lee, I-K. & Yun, B-S. Hispidin analogs from the mushroom *Inonotus xeranticus* and their free radical scavenging activity. *Bioorg. Med. Chem. Lett.* **16**, 2376-9 (2006).
22 Pizzolatti, M. G., Brighente, I. M. C., Bortoluzzi, A. J. O., Schripsema, J. & Verdi, L. G. Cyathenosin A, a spiropyranosyl derivative of protocatechuic acid from *Cyathea phalerata*. *Phytochemistry* **68**, 1327-30 (2007).
23 Lu, R-M., Min, C., Liao, P-Y & Wei, J-H. Chemical constituents in stem of Zhuang Medicine *Alsophila spinulosa*. *Chin. Tradit. Herbal Drugs* **44**,

2195-99 (2013).

24 Jung., J-Y. et al. Antioxidant polyphenols from the mycelial culture of the medicinal fungi *Inonotus xeranticus* and *Phellinus linteus*. *J. Appl. Microbiol.* **104**, 1824-32 (2008).

25 Ito, T. et al. Occurrence of stilbene oligomers in Cyperus rhizomes. *Fitoterapia* **83**, 1420-142 (2012).

26 Hu, Y. et al. Targeted isolation and tructure elucidation of stilbene glycosides from the bark of *Lysidice brevicalyx* Wei guided by biological and chemical screening. *J. Nat. Prod.* **71**, 1800-1805 (2008).

27 Xin et al. Chrysoxanthones A-C, three new xanthone-chromanone heterdimers from sponge-associated penicillium chrysogenum HLS111 treated with histone deacetylase inhibitor. *Mar. Drugs* **16**, 357 (2018).

28 Zhu et al. Hypocreaterpenes A and B, cadinane-type sesquiterpenes from a marine-derived fungus, Hypocreales sp. *Phytochem. Lett.* **6**, 392-396 (2013).

Supplementary Figures

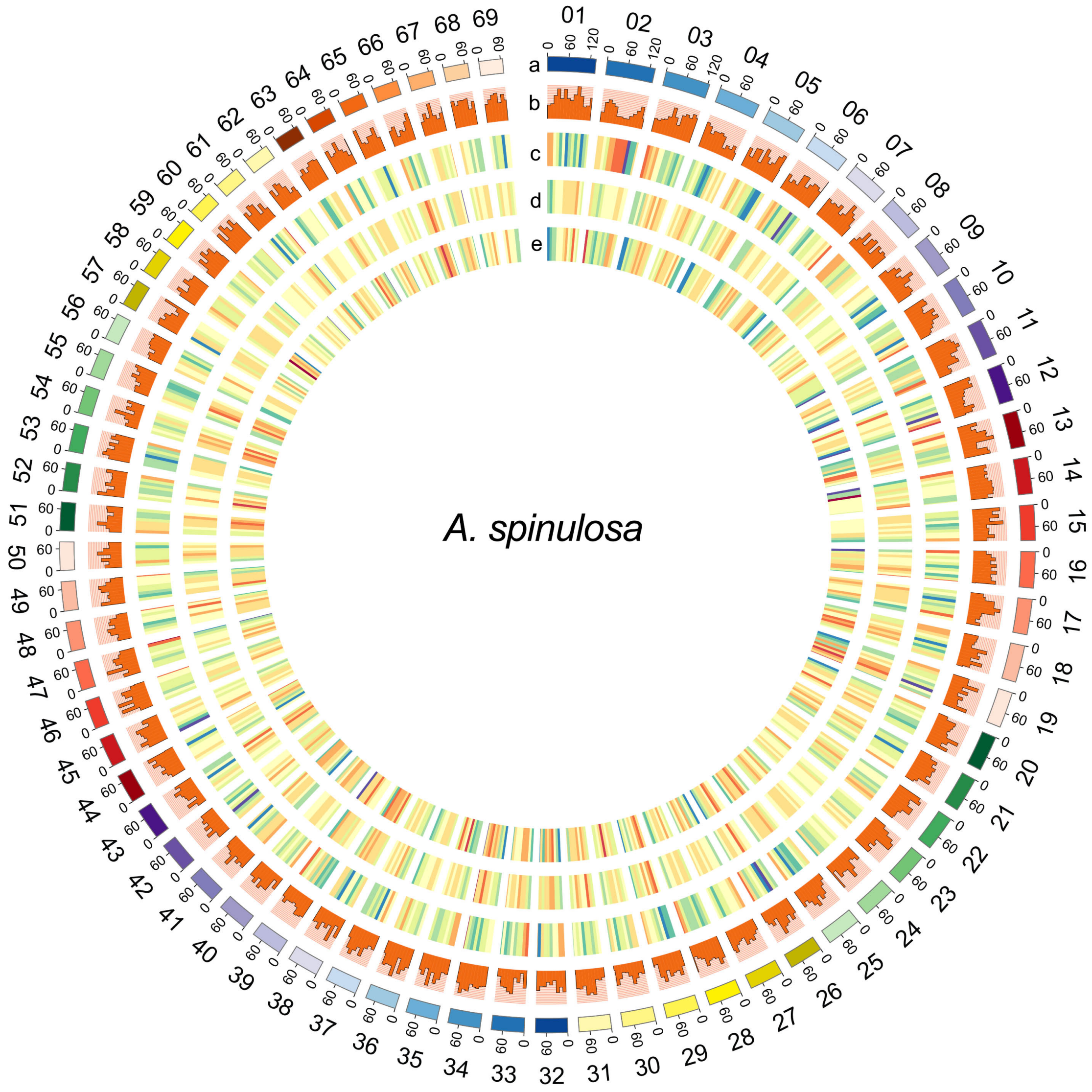

Supplementary Figure 1. Characteristics of *A. spinulosa* genome. a, Chromosome karyotype. **b**, Gene density. **c**, DNA transposon. **d**, Retrotransposon. **e**, GC content.

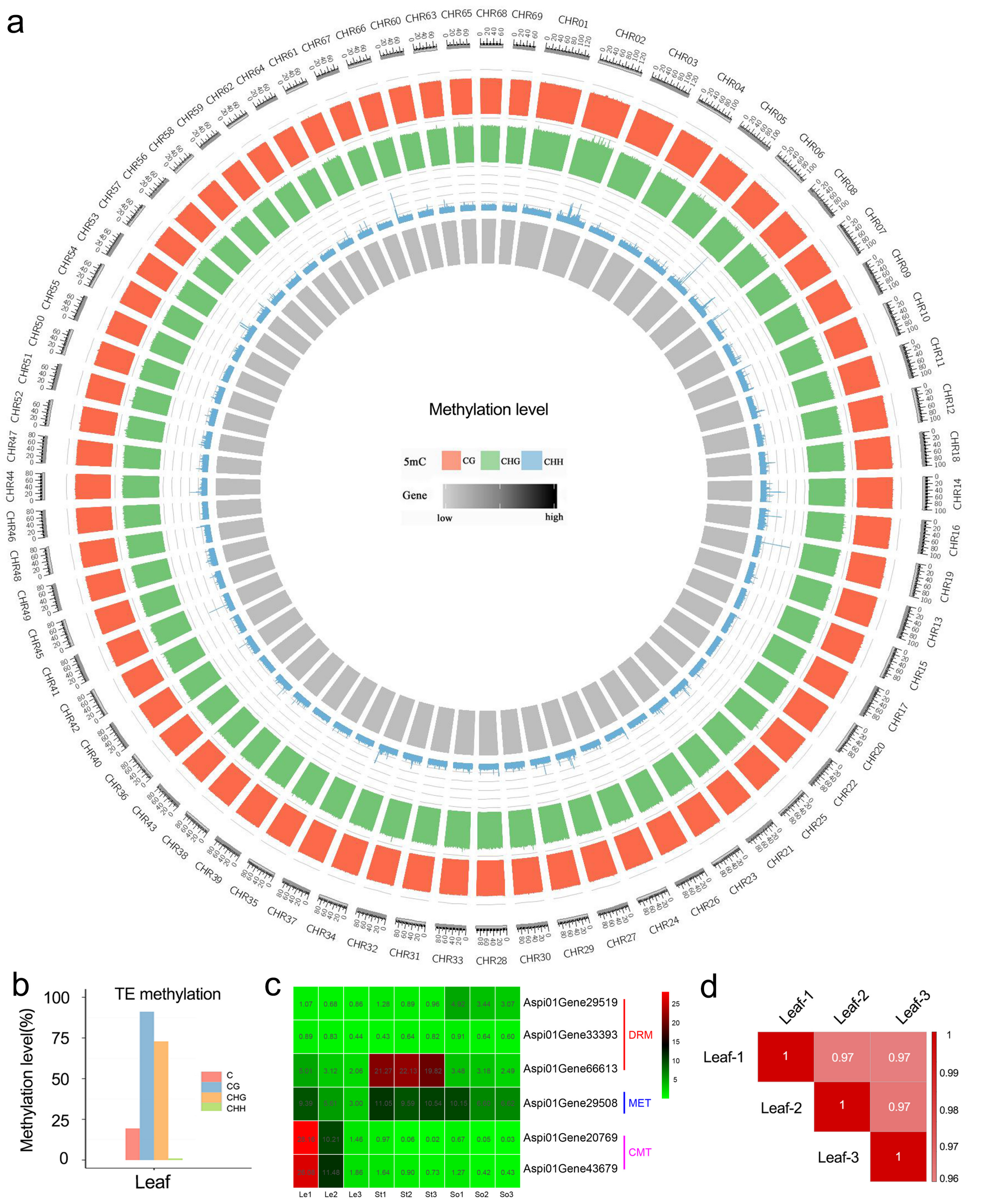

Supplementary Figure 2. Methylation analysis in *A. spinulosa* leaves. a, Circos plot of methylation level in the of context CG, CHG and CHH in 69 chromosomes. The layers from outer circle to inner circle represent chromosomes, mCG methylation level, mCHG level, mCHH level, and gene density, respectively. **b,** Bar plot of TE methylation levels of contexts C, CG, CHG and CHH. **c,** Heat map of FPKM values of 6 DNA methyltransferase genes in stems, leaves, and sori. St1/2/3: stem stage 1/2/3, Le1/2/3: leaf stage 1/2/3, So1/2/3: sorus stage 1/2/3. **d,** Pearson correlation of methylation level in the whole genome among three leaf samples. The high correlation coefficient ($R^2 > 0.97$) shows a good repeatability.

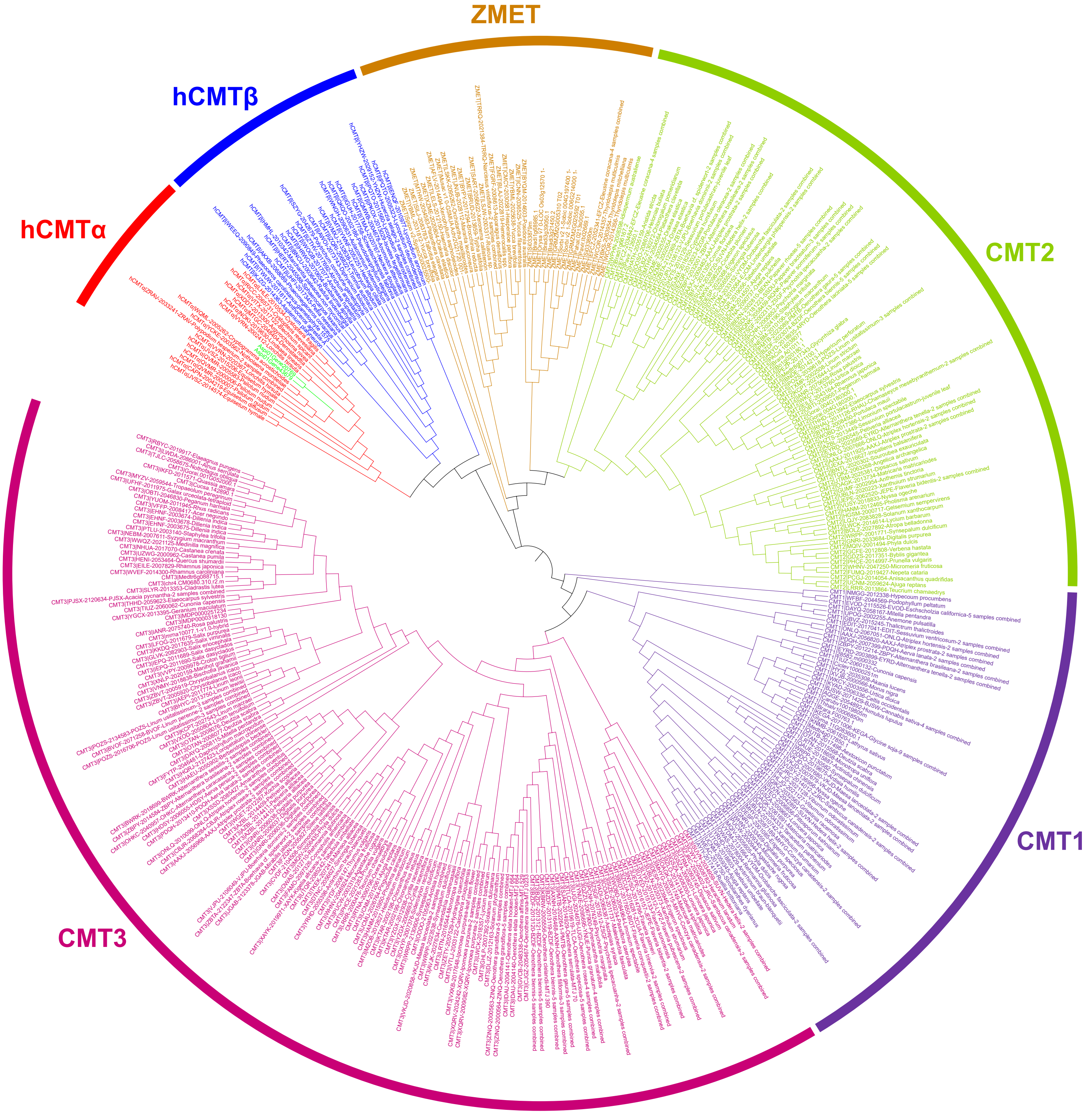

Supplementary Figure 3. Phylogenetic tree of CMT proteins from different species. These proteins are clustered into six groups (hCMTα, hCMTβ, ZMET, CMT1, CMT2, and CMT3). Two CMT proteins in *A. spinulosa*, highlighted in green, are in the hCMTα clade. The protein sequences were aligned by MUSCLE and the phylogenetic tree was constructed by RAxML using a maximum likelihood method.

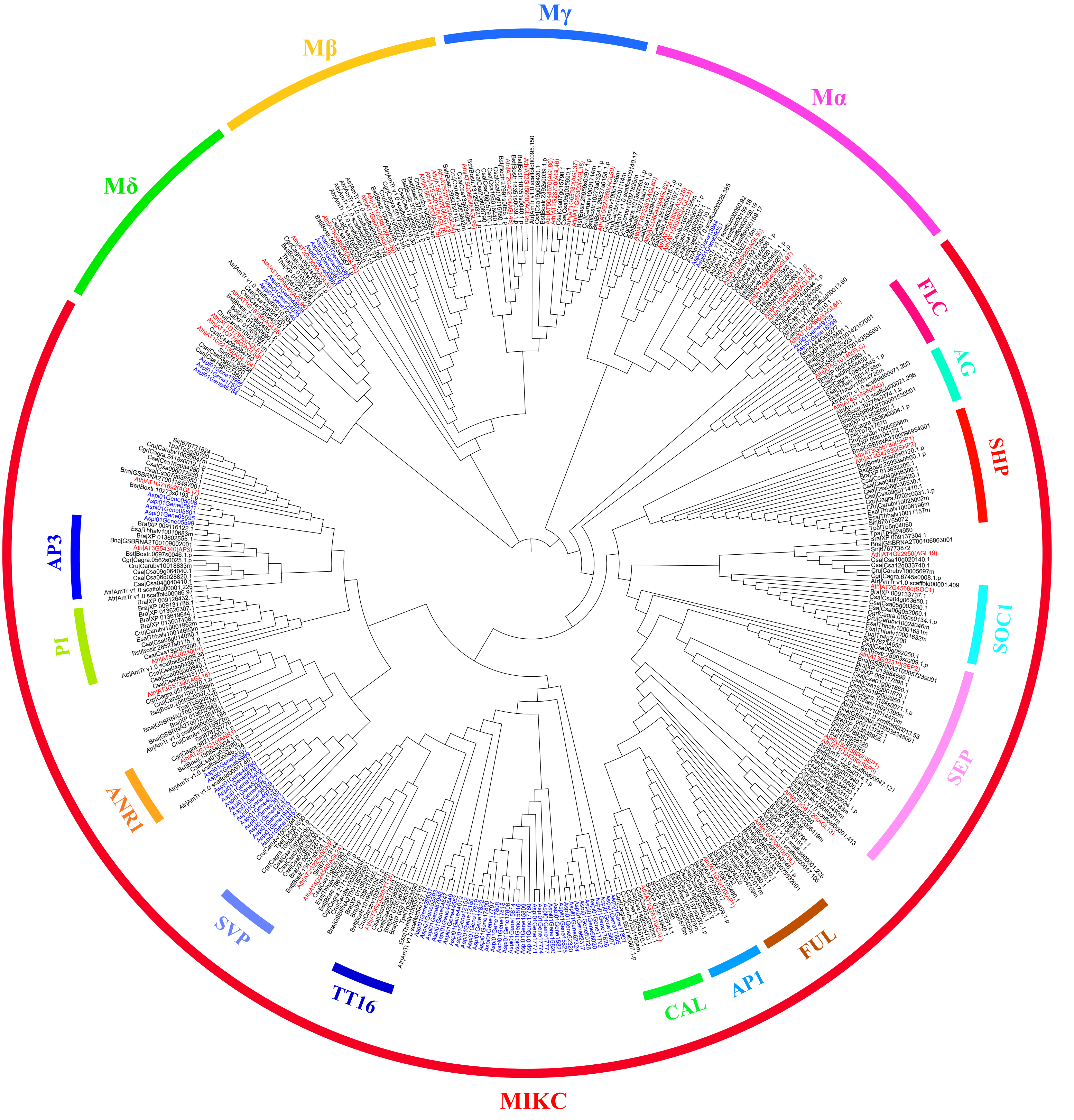

Supplementary Figure 4. Phylogenetic analysis of MADS-box proteins in *A. spinulosa* and other plants. Species name abbreviations are listed before protein names, Ath, *Arabidopsis thaliana*; Atr, *Amborella trichopoda*; Aar, *Aethionema arabicum*; Bst, *Boechera stricta*; Bna, *Brassica napus*; Bra, *Brassica rapa*; Bol, *Brassica oleracea*; Csa, *Camelina sativa*; Cgr, *Capsella grandiflora*; Cru, *Capsella rubella*; Esa, *Eutrema salsugineum*; Sir, *Sisymbrium irio*; Tpa, *Thellungiella parvula*; Tha, *Tarenaya hassleriana*. Proteins are clustered into five categories, including MIKC, Mα, Mβ, Mγ, Mδ. The Arabidopsis and *A. spinulosa* proteins are highlighted in red and blue, respectively. Protein sequences were aligned by MUSCLE and the phylogenetic tree was constructed by RAxML using a maximum likelihood method.

a

```
CreNOP10     MYLMYYDDDAGNRVYTLKKIAPDGTPTKSAHPARFSPDDKFSRERITCKKRFGLLPIQKPPPEL
SmNOP10-1    MYLMCYVNEEGQKVYTIKKEAPDGSPTESSHPARFSPDDKFSKQRVMLKKRFGILPTQKPPHEY
SmNOP10-2    MYLMCYVNEEGQKVYTIKKEAPDGSPTESSHPARFSPDDKFSKQRVMLKKRFGILPTQKPPHEY
StNOP10      MFLMCYINEEGQKVYTLKKELDDGTPTESSHPARFSPDDKCSKHRVILKKRFGLLPTQKPPQEY
MpNOP10      MYLMYYINENGSKVYTMKKEAPDGSPTMSAHPARFSPDDKFSKQRIICKRRFGLLPTQGPAHNY
AtNOP10      MYLQCYINEKGEKVYTTKKESPLGLATESAHPARFSPDDKYSKQRVLLKKRFGLLPTQNAPLQZ
PtNOP10-1    MYLQFYINDNGDKVYTTKKESPLGLPTQSAHPARFSPDDKYSRQRVLLKKRFGLLPTQQSPLKZ
PtNOP10-2    MYLQFYINDNGDKVYTTKKESPLGLPTESAHPARFSPDDKYSRQRFLLKKRFGLLPTQQSPLKY
PpNOP10      MYLMCYTNENGDKVYTLKKETPVGEATHSAHPARFSPDDKFSKHRIICKKRFNLLPTQQPPHNY
ScNOP10-1    MYLMCYTNENGDKVYTLQKESPEGGPTQSAHPARFSPDDKFSKHRVVLKKRFGLLPTQSPPPAY
ScNOP10-2    MYLMCYTNENGDK-----KESPEGGPTQSAHPARFSPDDKFSKHRVVLKKRFGLLPTQSPPPAY
AcNOP10      MYLMFYINENGDKVYTLQKDSPEGTPTQSAHPARFSPDDKFSKHRVVLKKRFGLLPTQSPPPTY
CriNOP10-1   MYLMFYINENGDKVYTLQKDSPEGSPTQSAHPARFSPDDKFSKHRVVLKKRFGLLPTQKPPPSY
CriNOP10-2   MYLMFYINENGDKVYTLQKDSPEGSPTQSAHPARFSPDDKFSKHRVVLKKRFGLLPTQKPPPTY
CriNOP10-3   MYLMFYINENGDKVYTLQKDSPEGSPTQSAHPARFSPDDKFSKHRVVLKKRFGLLPTQKPPPTY
GmNOP10      MYLMFYINDKGEKVYTLKKESPLGTPTQSAHPARFSPDDKFSRHRILLKKRFGLLPTQQPAPRY
AaNOP10      MYLMFYINDNGEKVYTLKKEDPAGTPSQSAHPARFSPDDKFSKQRITLKKRFGLLPTQAPPKEY
```

b

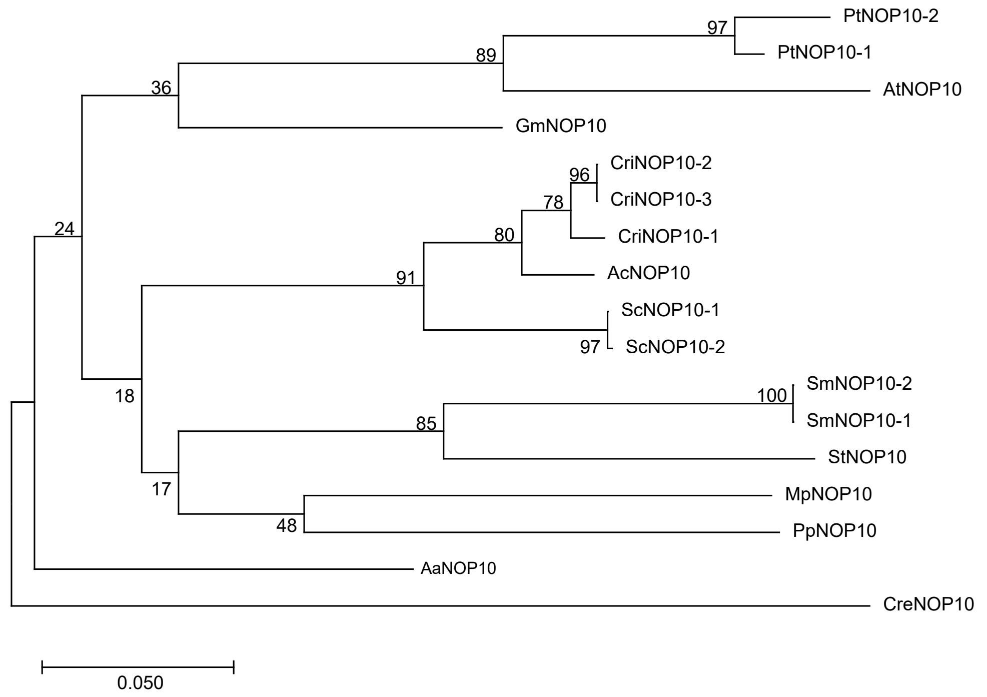

Supplementary Figure 5. Sequence alignment and phylogenetic analysis of NOP10 proteins in 12 plant species. a, protein sequences were aligned by MUSCLE. Cre: *Chlamydomonas reinhardtii*; Sm: *Selaginella moellendorffii*; St: *Selaginella tamariscina*; Pp: *Physcomitrella patens*; Sc: *Salvinia cucullata*; At: *Arabidopsis thaliana*; Pt: *Populus trichocarpa*; Mp: *Marchantia polymorpha*; Ac: *Adiantum capillus-veneris*; Cri: *Ceratopteris richardii*; Gm: *Gnetum montanum*; Aa: *Anthoceros angustus*. **b,** A neighbor joining (NJ) phylogenetic tree was constructed using Mega-X with 1000 bootstrap replicates.

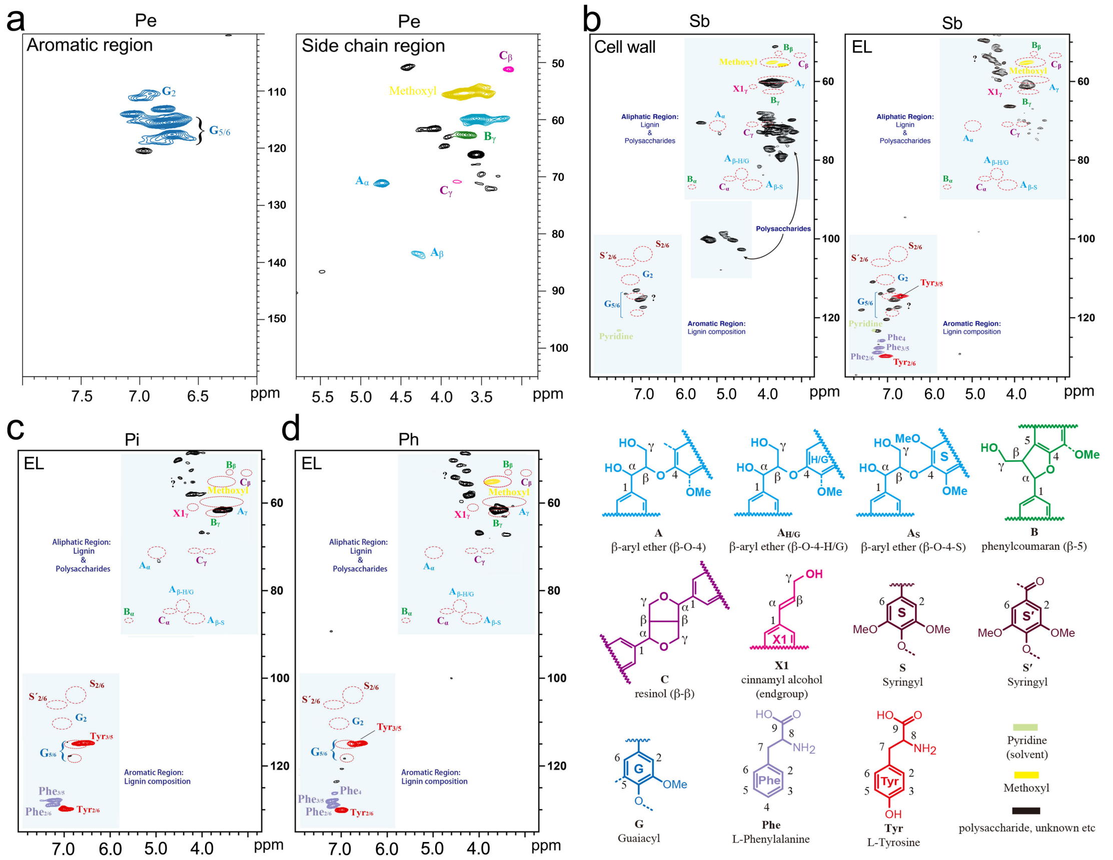

Supplementary Figure 6. Lignin structures analysis in petiole (Pe), sclerenchymatic belt (Sb), pith (Pi) and phloem (Ph) of *A. spinulosa* analyzed by 2D HSQC NMR. a, NMR analysis of petiole. G-type monolignol in aromatic region and some chemical bonds such as β-O-4, β-β in side chain region were detected. **b,** NMR analysis of sclerenchymatic belt. No lignin components were found in the whole cell wall gel-sample. All possible lignin components are marked with red dot lines, and the chemical structures of the lignin subunits are color-coded to show their possible signal assignments in the spectra. None of the correlations from the sample match with the lignin components except methoxyl group. All lignin region of 2D HSQC NMR spectra of enzyme lignin (EL) from sclerenchymatic belt. No lignin components are shown from the enzyme lignin sample. The polysaccharides disappear after the cellulose treatment. **c,** NMR analysis of pith. No lignin components are shown from the EL sample. **d,** NMR analysis of phloem. No lignin components are shown from the EL sample.

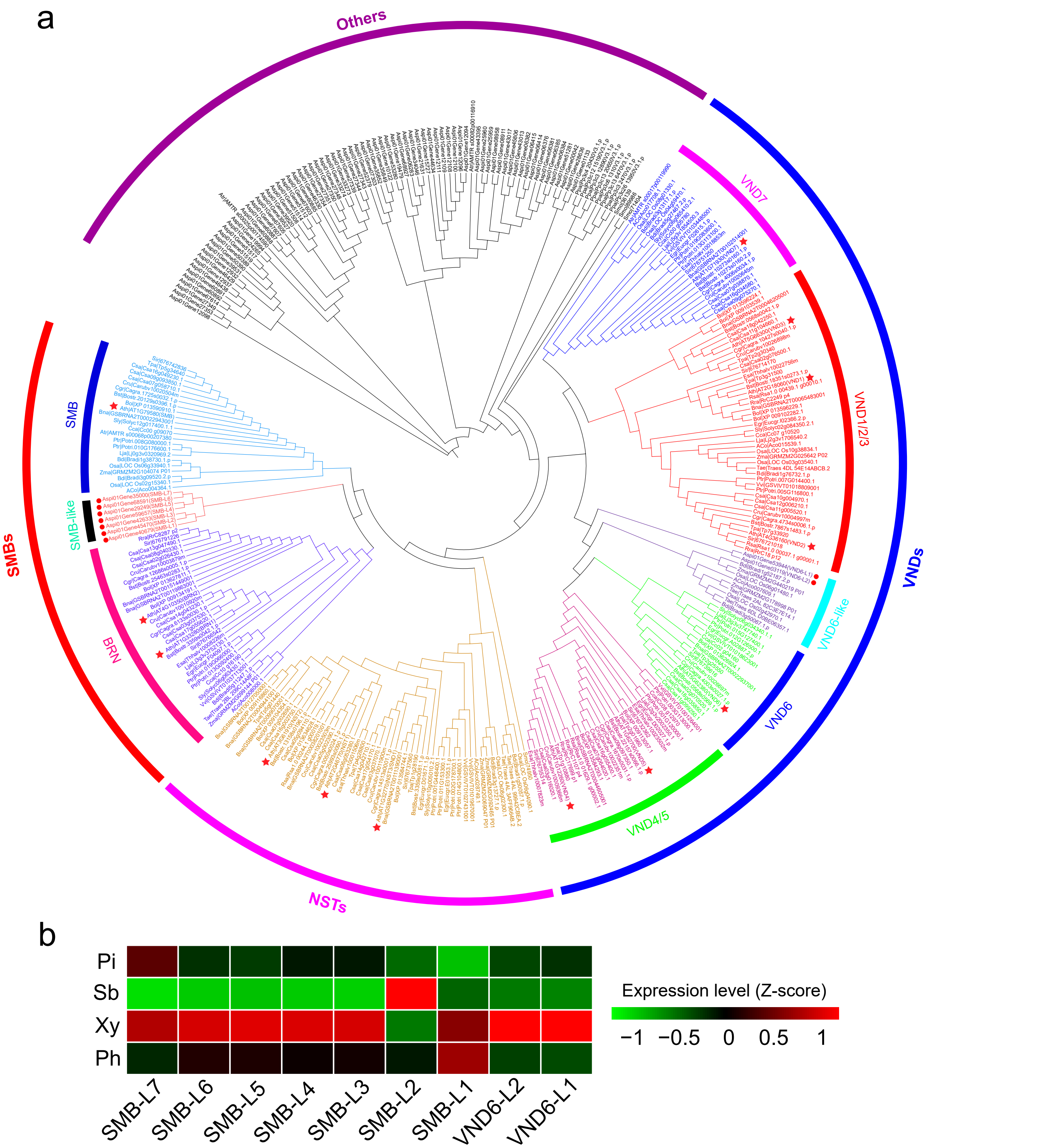

Supplementary Figure 7. Phylogenetic analysis of NAC proteins in *A. spinulosa* and other plants. a, NAC protein sequences are from *Arabidopsis thaliana* (Ath), *Amborella trichopoda* (Atr), *Ananas comosus* (Ao), *Oryza sativa* (Osa), *Populus trichocarpa* (Ptr), *Vitis vinifera* (Vvi), *Zea mays* (Zma), *Triticum aestivum* (Tae), *Eucalyptus grandis* (Egr), *Coffea canephora* (Cca), *Solanum lycopersicum* (Sly), *Lotus japonicas* (Lja), *Brachypodium distachyon* (Bdi), *Physcomitrium patens* (Ppa), *Selaginella moellendorffii* (Smo), *Boechera stricta* (Bst), *Brassica napus* (Bna), *Brassica oleracea* (Bol), *Camelina sativa* (Csa), *Capsella grandiflora* (Cgr), *Capsella rubella* (Cru), *Eutrema salsugineum* (Esa), *Raphanus raphanistrum* (Rra), *Raphanus sativus* (Rsa), *Sisymbrium irio* (Sir), and *Thellungiella parvula* (Tpa). Protein sequences were aligned by MUSCLE and the phylogenetic tree was constructed using RAxML using a maximum likelihood method. According to the phylogenetic relationship, all proteins are clustered into four categories, including VNDs, SMBs, NSTs, and Others. Two *A. spinulosa* genes, marked with red circles, were clustered in the VND clade. Seven *A. spinulosa* genes, marked with red stars, are clustered in the SMB clade. **b,** Heat map shows the relative expression levels of seven SMB-like and two VND6-like genes in pith (Pi), sclerenchymatic belt (Sb), xylem (Xy), and phloem (Ph) of *A. spinulosa*. FPKM values were normalized using the Z-score method.

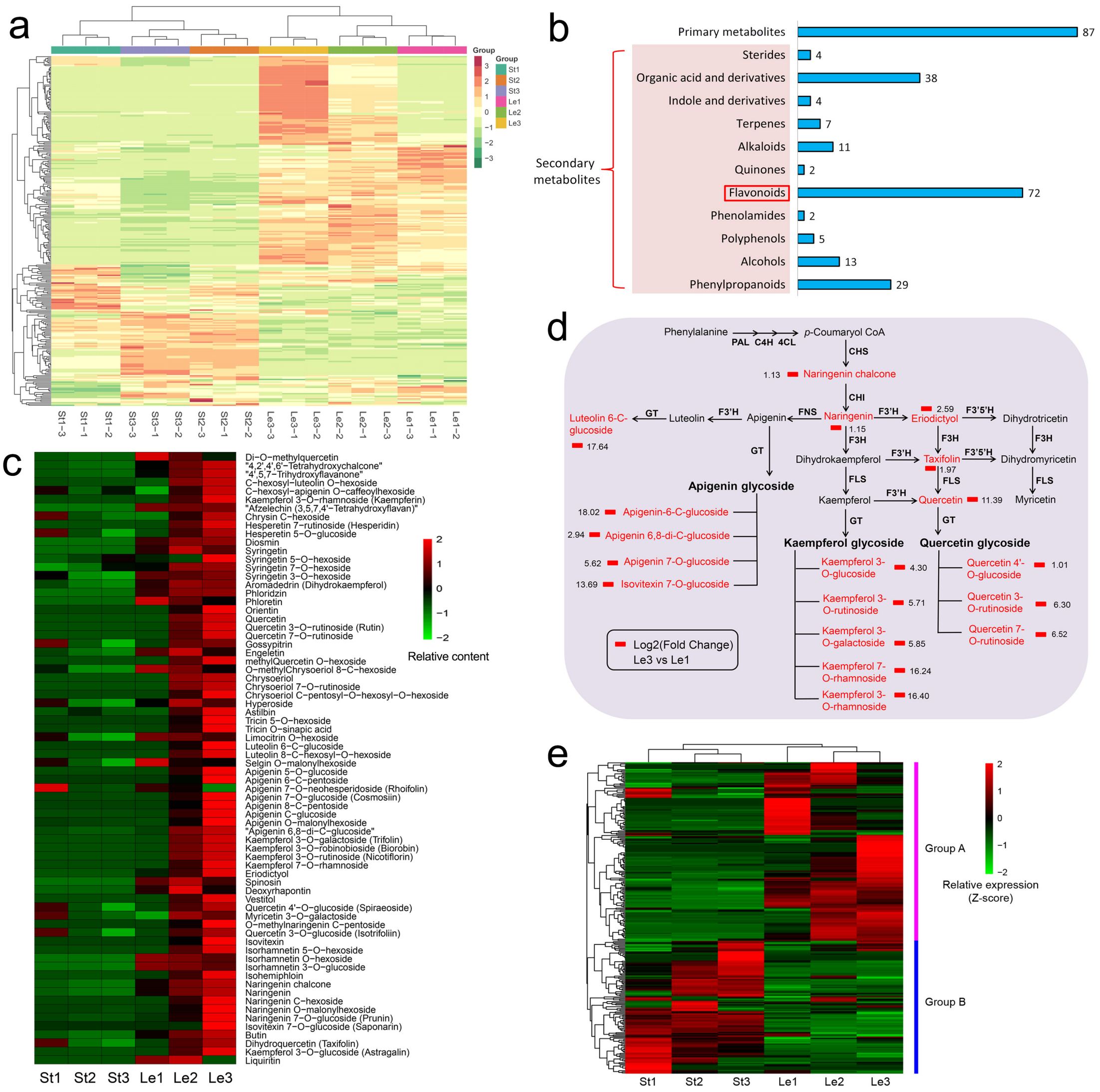

Supplementary Figure 8. Characterizations of secondary metabolites in leaves and stems of *A. spinulosa*. **a,** Abundances of 274 metabolites detected by widely targeted metabolomics. Relative abundances are normalized and used in hierarchical cluster analysis using R. 18 samples are clustered to 6 groups and each group contains three biological replicates. St1/2/3 (stem stage 1/2/3), Le1/2/3 (leaf stage 1/2/3). **b,** Histogram shows the category of 274 metabolites, including 87 primary and 187 secondary metabolites. The flavonoids constitute the majority (38.5% of the secondary metabolites). **c,** Heat map shows the relative contents of 72 flavonoid metabolites detected in stems and leaves of *A. spinulosa* by the technology of widely targeted metabolomics. The counts are normalized using Z-score method. **d,** Flavonoid biosynthesis pathway in leaves. Small red cells represent log2 (fold change) of metabolites between Le3 and Le1. PAL: phenylalanine ammonia-lyase; C4H: cinnamate-4-hydroxylase; 4CL: 4-coumarate CoA ligase; CHS: chalcone synthase; CHI: chalcone isomerase; F3H: flavanone 3-hydroxylase; F3′H: flavonoid 3′-hydroxylase; F3′5′H: flavonoid 3′, 5′-hydroxylase; FLS: flavonol synthase; FNS:

flavone synthase; GT: glycosyltransferase. **e,** Heat map shows relative expression of genes related to flavonoid biosynthetic pathway in stems and leaves of *A. spinulosa*. FPKM values were normalized using Z-score method. 358 pathway enzyme genes identified in the *A. spinulosa* genome, including 8 *CHS* genes, 4 *CHI* genes, 10 *F3H* genes, 10 *FLS* genes, 21 *F3'H* genes, 6 *F3'5'H* genes, 46 *FNS* genes, and 253 *GT* genes, are clustered into two groups (A and B) according to the expression patterns. The genes in group A and B are highly expressed in leaves and stems, respectively.

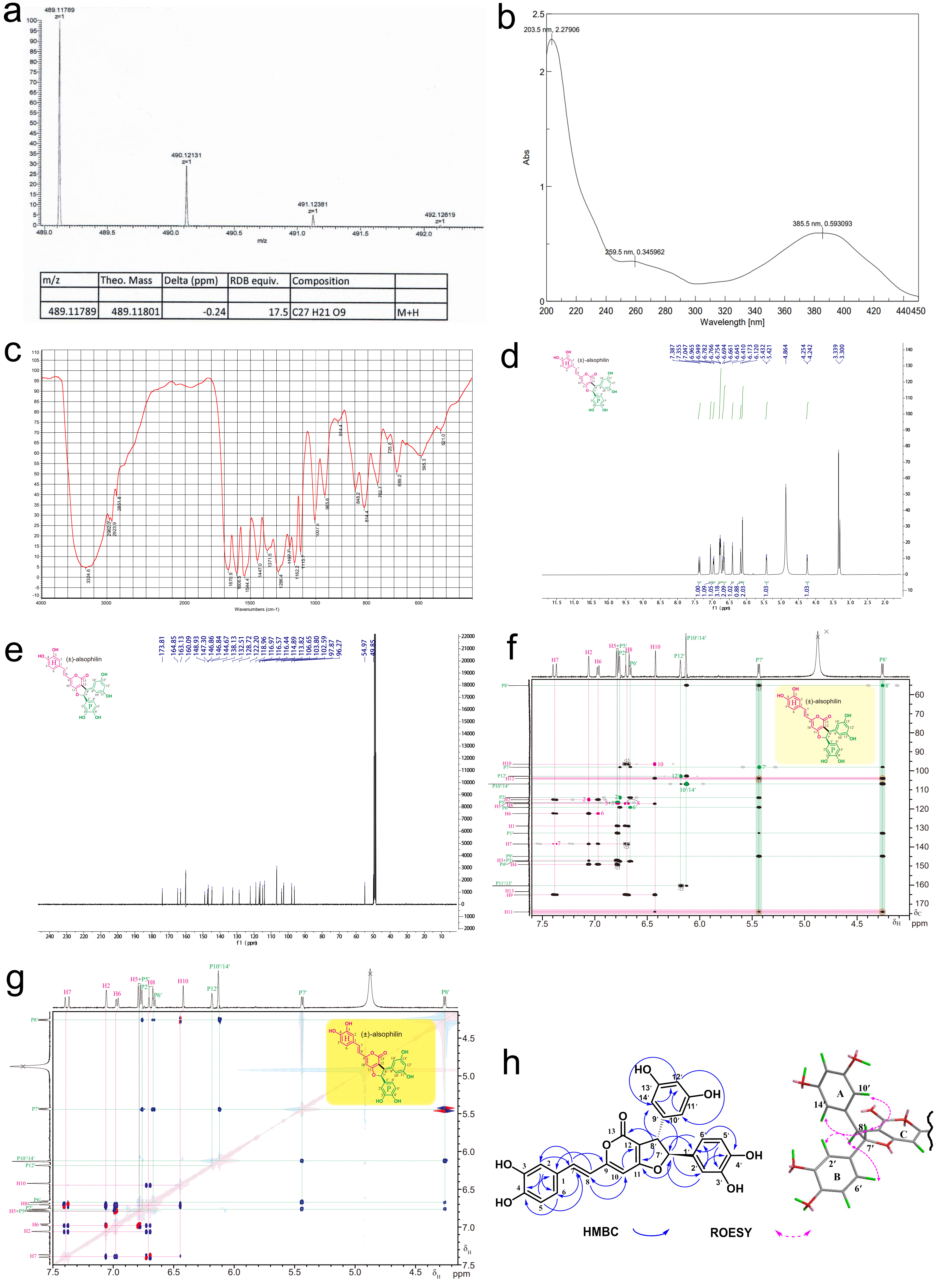

m/z	Theo. Mass	Delta (ppm)	RDB equiv.	Composition	
489.11789	489.11801	-0.24	17.5	C27 H21 O9	M+H

Supplementary Figure 9. The spectra of (±)-alsophilin for its structure determination. **a,** HRESIMS spectrum of (±)-alsophilin, measured on a Thermo Scientific Q Exactive Focus Orbitrap mass spectrometer. **b,** Ultraviolet (UV) spectrum of (±)-alsophilin, measured on a JASCO J-815 CD spectropolarimeters. UV (MeOH) λ_{max} (log ε) 203.5 (2.28), 259.5 (0.35), and 385.5 (0.59) nm. **c,** Infrared (IR) spectrum of (±)-alsophilin, acquired by KBr disk method on a Shimadzu FTIR-8400S spectrometer. IR (KBr) νmax 3324, 1671, 1607, 1544, 1447, 1286, 1162, 1115, 1007, 965, 848, 814, 762, 689 cm^{-1}. **d,** ^{1}H NMR (nuclear magnetic resonance) spectrum (500 MHz) of (±)-alsophilin, recorded on a Bruker AV-500 spectrometer using deuterated solvent CD_3OD with TMS as an internal standard, and ^{1}H NMR spectral data are shown in Supplementary Table 21. **e,** ^{13}C NMR spectrum (125 MHz) of (±)-alsophilin, recorded on a Bruker AV-500 spectrometer using deuterated solvent CD_3OD with TMS as an internal standard, and ^{13}C NMR spectral data were shown in Supplementary Table 21. **f,** ^{1}H–^{13}C correlation spectra of (±)-alsophilin, with 1D ^{1}H and ^{13}C spectra on the horizontal and vertical axes; acquired on a Bruker AV-500, 500 MHz spectrometer, in CD_3OD (central solvent peaks used as references. HSQC correlations are overlaid on the HMBC spectra; magenta contours are for the 1-bond ^{1}H–^{13}C correlations of protonated carbons for the hispidin (H) moiety, green contours for the piceatannol (P) moiety. HMBC spectra (black) showing 2- and 3-bond correlations that aided the assignments. The correlations (highlighted in green) from protons P7′ and P8′ are particularly useful, those correlating with carbons H12 and H11 (magenta highlighting and circled) being especially diagnostic for establishing the phenylcoumaran nature of the product derived from P8′–H12 radical coupling. Boxes labeled ×2.5 are lower-level contours (expanded 2.5×) useful for assignments. Contours colored light gray are from residual 1-bond correlations (split by the 1-bond ^{1}H–^{13}C coupling constant) that are not minimized in the used HMBC experiment. Assignments for the 1D proton and carbon spectral projections are numbered and color-coded to match the (±)-alsophilin. **g,** ROESY spectrum of (±)-alsophilin, recorded on a Bruker AV-500 spectrometer using deuterated solvent CD_3OD with TMS as an internal standard. Important cross-peaks are highlighted by being dark blue (positive) and red (negative); their lighter colors are from the diagonal and less-important attributes of the spectrum. Assignments for the 1D proton and carbon spectral projections are numbered and color-coded to match the (±)-alsophilin. Note that the proton spectrum used for the projections is not run on the same sample nor at the same time; some peaks have therefore been moved. We use the dotted lines to show the offset. **h,** Key HMBC and ROESY correlations of (±)-alsophilin. The relative configuration of (±)-alsophilin is determined to be *trans* orientation between protons P7′ and P8′ from the key ROESY correlations between protons P7′/P10′(P14′) and P8′/P2′.

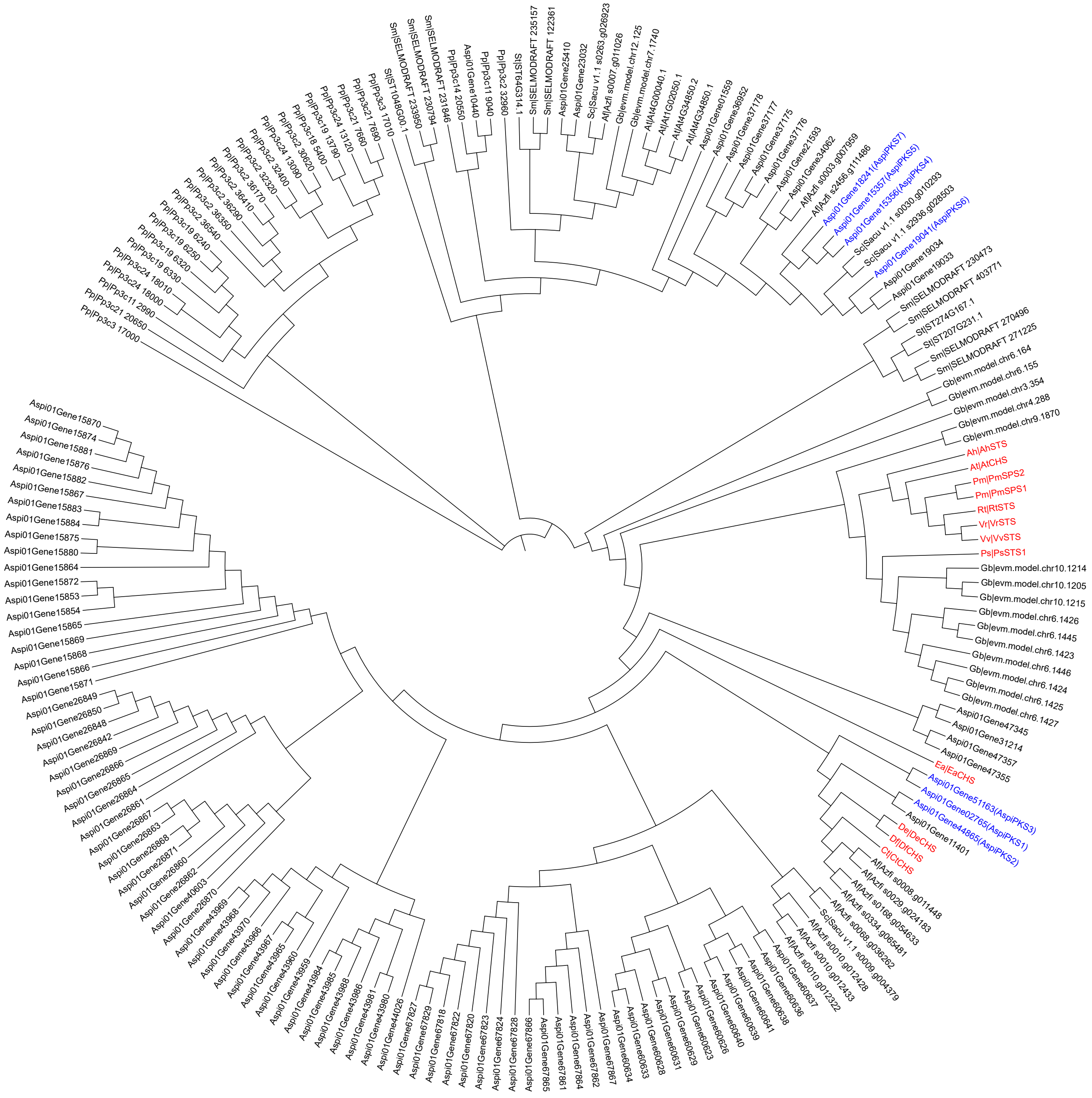

Supplementary Figure 10. Phylogenetic relationships of PKS III proteins in different species. The abbreviations before protein names represent different species, At: *Arabidopsis thaliana*, Gb: *Ginkgo biloba*, Sm: *Selaginella moellendorffii*, St: *Selaginella tamariscina*, Af: *Azolla filiculoides*, Sc: *Salvinia cucullata*, Pp: *Physcomitrella patens*, Pm: *Piper methysticum*, Ct: *Ceratopter-is thalictroides*, De: *Dryopteris erythrosora*, Df: *Dryopteris fragrans*, Ea: *Equisetum arvense*, Ps: *Pinus sylvestris*, Ah: *Arachis hypogaea*, Vr: *Vitis riparia*, Vv: *Vitis vinifera*, Rt: *Rheum tataricum*. The red genes represent reported function-known proteins, with GenBank accession numbers: CtCHS (AFN02448), DeCHS (AHU87077), DfCHS (AHA85054), EaCHS (BAA89501), AtCHS (BAD89858), AhSTS (BAA78617), RtSTS (AAP13782), VrSTS (AAF00586), VvSTS (CAA54221), PmSPS1 (QCX36371), PmSPS2 (QCX36372), and PsSTS1 (P48407). Proteins highlighted in blue have detectable enzyme activities *in vitro*. Protein sequences were aligned by MUSCLE and the phylogenetic tree was constructed by RAxML using a maximum likelihood method.

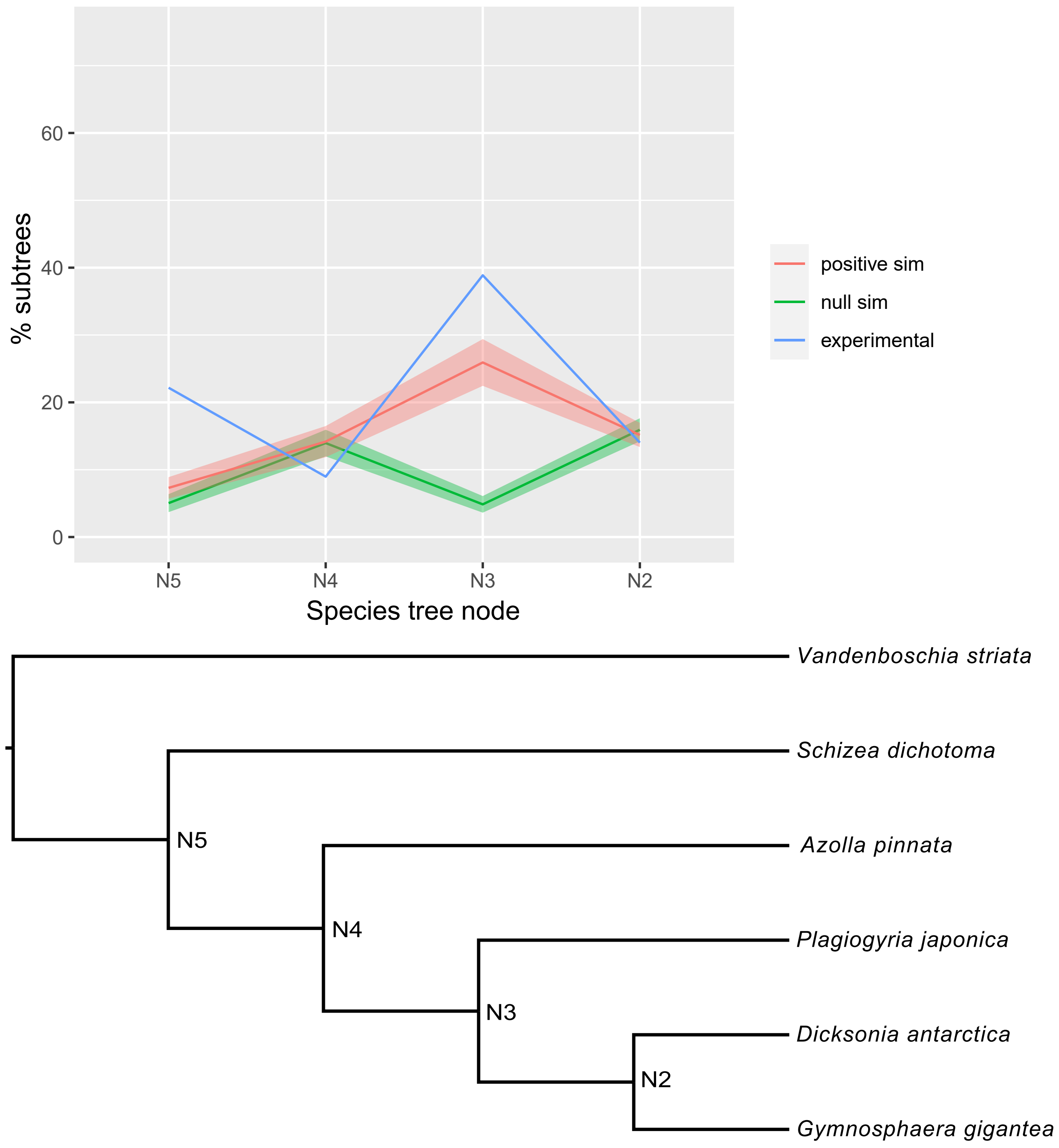

Supplementary Figure 11. MAPS analysis of Cyatheales WGD event excluding *A. spinulosa*. A Cyatheaeles-wide WGD event was still detected when *A. spinulosa* was excluded from the MAPS analysis. The percentage of subtrees supporting a WGD at or before N3 was significantly higher than null and positive simulations according to an one-sided Fischer's Exact Test (P <0.005). The dark lines in the center of the shaded regions represent the average values for null and positive gene tree simulations.

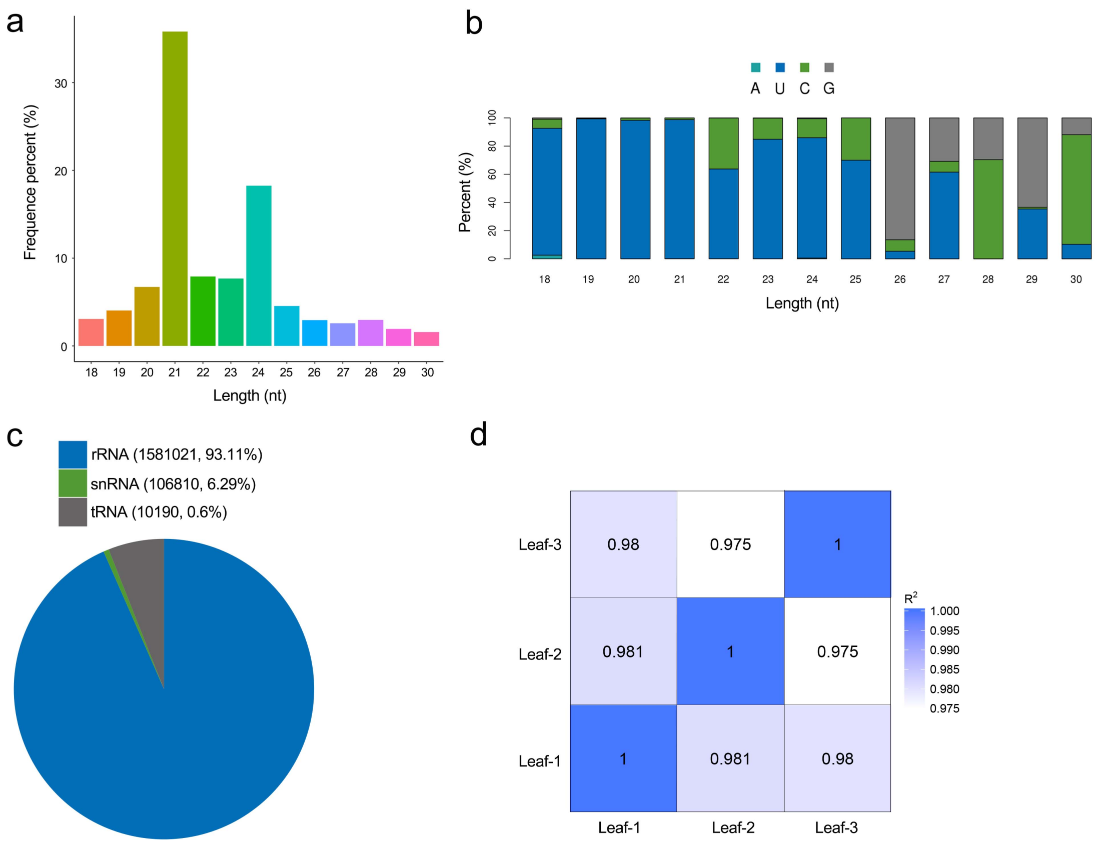

Supplementary Figure 12. Characterization of small RNA in *A. spinulosa* leaves. **a,** Length distribution of small RNAs. 21 nt length of sRNA reads are the most abundant, occupying 34.72%. **b,** First nucleotide bias of miRNAs, mainly trending to be uracil. **c,** Annotation of non-coding RNAs (ncRNAs), including rRNA, tRNA, snRNA and snoRNA. Of which, 93.11% are rRNAs and there is no snoRNA in *A. spinulosa*. **d,** Pearson correlation analysis among three leaf samples shows a high correlation coefficient of miRNA TPM values ($R^2 > 0.97$), indicating a quite good repeatability.

Supplementary Tables

Supplementary Table 1. Statistics of clean sequencing data generated from the PacBio Sequel platforms, Illumina and Hi-C sequencing in *A. spinulosa*.

**PacBio data**					
**Platform**	**Insert size (kb)**	**SMRT cell number**	**Clean data (Gb)**	**N50 length (bp)**	**Depth[1] (X)**
Sequel I	20	73	442.72	15,543	71.06
Sequel II	20	3	460.15	23,021	73.86
Total	-	76	902.87	-	144.92

**Illumina data**					
**Insert size**	**Type**	**Reads length (bp)**	**No. of clean reads**	**Total clean bases (bp)**	**Depth[1] (X)**
270 bp	Paired-end	150	2,577,465,130	386,619,769,500	62.06
500 bp	Paired-end	150	2,644,833,362	396,725,004,300	63.68
Total	-	-	5,222,298,492	783,344,773,800	125.74

**Hi-C data**				
**Type**	**Read number**	**Base number (bp)**	**Ratio (%)**	**Depth[1] (X)**
Total clean data	1,331,223,757	399,367,127,100	-	64.10
Mapped paired-end reads	1,047,707,917	314,312,375,100	78.70	-
Unmapped paired-end reads	10,364,629	3,109,388,700	0.78	-
Valid data	301,657,590	120,497,277,000	30.17	-

[1]Depth was calculated under the assumption of a genome size of 6.23 Gb.

Supplementary Table 2. Statistics for the chromosome-level assembly of the *A. spinulosa* genome.

	Scaffold length (bp)	Number	Contig* length (bp)	Number
N10[1]	112,454,994	5	4,372,444	112
N20	104,380,601	11	3,288,578	277
N30	100,365,623	17	2,700,726	488
N40	95,373,544	24	2,220,203	741
N50	92,484,196	30	1,795,171	1,056
N60	88,178,156	37	1,417,007	1,445
N70	81,981,237	45	1,073,001	1,956
N80	79,385,870	52	749,984	2,651
N90	71,293,965	61	419,601	3,737
Maximum length	135,221,733	-	9,774,507	-
Total length	6,234,337,617	-	6,230,791,617	-
Total number	-	69	-	7161
GC content	42.1%	-	42.1%	-

[1]N10 refers to the size above which 10% of the total length of the sequence assembly can be found. From N20 to N90 are similarly defined. * indicates contig after scaffolding.

Supplementary Table 3. BUSCO assessments of *A. spinulosa* genome assembly and annotation using Eukaryota_odb10 dataset (10 September, 2020).

**Assembly assessment**		
	**Number**	**Percentage (%)**
Complete BUSCOs	249	97.6
Complete and single-copy BUSCOs	203	79.6
Complete and duplicated BUSCOs	46	18.0
Fragmented BUSCOs	3	1.2
Missing BUSCOs	3	1.2
Total BUSCOs groups searched	255	-
**Annotation assessment**		
	**Number**	**Percentage (%)**
Complete BUSCOs	184	72.1
Complete and single-copy BUSCOs	151	59.2
Complete and duplicated BUSCOs	33	12.9
Fragmented BUSCOs	55	21.6
Missing BUSCOs	16	6.3
Total BUSCOs groups searched	255	-

Supplementary Table 4. Statistics of repeat sequences in *A. spinulosa* genome.

	Length (bp)	% of genome
Class I: Retrotransposon	2,520,569,120	40.43
LTR-Retrotransposon	2,353,496,477	37.75
LTR/Copia	777,705,955	12.47
LTR/Gypsy	1,553,536,402	24.91
LTR-other	22,254,120	0.37
Non-LTR Retrotransposon	167,072,643	2.70
SINE	2,103,182	0.03
LINE	166,664,081	2.67
Class II:DNA Transposon	157,368,721	2.52
CMC-EnSpm	37,224,336	0.59
MULE-MuDR	16,810,091	0.26
hAT-Tag1	12,699,458	0.20
TcMar-Tc2	12,573,034	0.20
hAT	12,247,640	0.19
TcMar-Tc1	11,945,278	0.19
Sola-1	11,337,487	0.18
PIF-Harbinger	7,273,667	0.11
Sola-2	4,807,706	0.07
Low Complexity	28,370,389	0.45
Tandem repeat	663,557,047	10.64
Unknown	1,286,756,815	20.63
Total content	4,685,835,730	75.15

Supplementary Table 5. Statistics of gene numbers and features in *A. spinulosa.*

**Gene numbers and features**	
Gene_count	67,831
Gene_max	207,196 bp
Gene_min	102 bp
Gene_Median / Average	2,382 / 9,542.41 bp
CDS_count	67,831
CDS_max	47,169 bp
CDS_min	102 bp
CDS_Median / Average	879 / 1,135.58 bp
cDNA_count	67,831
cDNA _max	48,726 bp
cDNA _min	102 bp
cDNA _Median / Average	1,095 / 1,396.61 bp
mRNA_count	71,488
mRNA _max	207,196 bp
mRNA _min	102 bp
mRNA _Median / Average	2,472 / 9,762.09 bp
mRNA_count_Median / Average	1 / 1.03
UTR3_ Median / Average	296 / 392.35 bp
UTR5_ Median / Average	137 / 284.47 bp
Exon_Median / Average	553 / 711.40 bp
Exon_count_Median / Average	2 / 3.76
Intron_Median / Average	566 / 8,130.30 bp
Intron_count_Median / Average	1 / 2.61

Supplementary Table 6. Gene functional annotation in the *A. spinulosa* genome.

Dataset	Number	Percent (%)
Nr	63,551	93.69
Swiss-Prot	35,772	52.73
KOG	14,040	20.69
eggNOG	58,208	85.81
InterPro	52,284	77.07
Pfam	44,813	66.06
GO	35,549	52.4
KEGG	9,665	14.24
All functional genes	64,688	95.36

Supplementary Table 7. Genome structure comparisons between *Alsophila spinulosa* and *Arabidopsis thaliana* TAIR10.

	A. spinulosa	***A. thaliana***
Total gene number	67,831	27,655
Average gene length (bp)	9,542.41	2,373.54
Average CDS length (bp)	1,135.58	1,201.52
Average exon number per gene	3.76	5.28
Average exon length (bp)	711.40	1620.03
Average intron length (bp)	8,130.30	709.71

Supplementary Table 8. Statistics of gene family numbers related to lignin biosynthesis among *Alsophila spinulosa*, *Azolla filiculoides*, and *Salvinia cucullata*.

Gene family	*A. spinulosa*	*A. filiculoides*	*S. cucullata*
PAL	22	9	6
4CL	50	26	26
CCR	42	25	20
CAD	51	20	21
CCoAOMT	9	3	2
COMT	33	7	5
CSE	37	26	16
HCT	130	15	18
P450	331	100	77

Supplementary Table 9. 17 pairs of WGD genes in 8 gene families related to lignin biosynthetic pathway.

Gene_1	Gene_2	Gene family	Median block Ks	Collinear (+/−)
Aspi01Gene07514 (*AspiPAL5a*)	Aspi01Gene05547 (*AspiPAL5b*)	*PAL*	0.4526	+
Aspi01Gene12760 (*Aspi4CL1a*)	Aspi01Gene46333 (*Aspi4CL1b*)	*4CL*	0.3885	+
Aspi01Gene22852 (*Aspi4CL2a*)	Aspi01Gene25649 (*Aspi4CL2b*)	*4CL*	0.4316	+
Aspi01Gene09501 (*AspiCAD2a*)	Aspi01Gene43243 (*AspiCAD2b*)	*CAD*	0.3598	+
Aspi01Gene29611 (*AspiCAD3a*)	Aspi01Gene66730 (*AspiCAD3b*)	*CAD*	0.2681	+
Aspi01Gene01252 (*AspiCCR1a*)	Aspi01Gene28475 (*AspiCCR1b*)	*CCR*	0.3924	+
Aspi01Gene54723 (*AspiCCR2a*)	Aspi01Gene71938 (*AspiCCR2b*)	*CCR*	0.3289	+
Aspi01Gene01343 (*AspiCCR3a*)	Aspi01Gene28411 (*AspiCCR3b*)	*CCR*	0.3997	+
Aspi01Gene01596 (*AspiCCR4a*)	Aspi01Gene28121 (*AspiCCR4b*)	*CCR*	0.3457	+
Aspi01Gene24006 (*AspiCCR5a*)	Aspi01Gene20254 (*AspiCCR5b*)	*CCR*	0.4534	+
Aspi01Gene29511 (*AspiCCR6a*)	Aspi01Gene66601 (*AspiCCR6b*)	*CCR*	0.3079	+
Aspi01Gene43044 (*AspiCCoAOMT3a*)	Aspi01Gene45864 (*AspiCCoAOMT3b*)	*CCoAOMT*	0.4328	+
Aspi01Gene53484 (*AspiCSE2a*)	Aspi01Gene19235 (*AspiCSE2b*)	*CSE*	0.4093	+
Aspi01Gene31960 (*AspiCSE3a*)	Aspi01Gene30587 (*AspiCSE3b*)	*CSE*	0.4054	+
Aspi01Gene50432 (*AspiHCT1a*)	Aspi01Gene62633 (*AspiHCT1b*)	*HCT*	0.3389	+
Aspi01Gene09518 (*AspiHCT2a*)	Aspi01Gene43257 (*AspiHCT2b*)	*HCT*	0.3598	+
Aspi01Gene72914 (*AspiC3H1a*)	Aspi01Gene37809 (*AspiC3H1b*)	*C3H*	0.4842	+

Supplementary Table 10. Numbers of 15 gene families expanded in *A. spinulosa* compared to *A. filiculoides* and *S. cucullata* by OrthoFinder analysis. Peroxidase (Hmm: peroxidase, PF00141); MYB (Hmm: MYB_DNA-binding, PF00249); NAC (Hmm: NAM, PF02365); MADS-box (Hmm: SRF-TF, PF00319); bHLH (Basic helix-loop-helix, Hmm: HLH, PF00010); Cupin_1 (Hmm: Cupin_1, PF00190); POT (Proton-dependent oligopeptide transporter, Hmm: PTR2, PF00854); SAUR (Small auxin-up RNA, Hmm: Auxin inducible, PF02519); HMA (Heavy metal associated, Hmm: HMA, PF00403); NPH3 (Non-phototropic hypocotyl 3, Hmm: NPH3, PF03000); PE (Pectinesterase, Hmm: Pectinesterase, PF01095); HSP20 (Heat shock protein, Hmm: HSP20, PF00011); LOB (Lateral organ boundaries, Hmm: LOB, PF03195); GATA (GATA zinc finger, Hmm: GATA, PF00320); GH3 (GH3 auxin-responsive promoter, Hmm: GH3, PF03321).

**Gene family**	***C. braunii***	***A. angustus***	***M. polymorpha***	***P patens***	***S. cucullata***	***A. filiculoide***	***A. spinulosa***	***G. montanum***	***G. biloba***	***A. trichopoda***	***A. thaliana***	***S. tamariscina***
Peroxidase	12	32	115	42	21	24	82	53	85	48	66	53
MYB	6	12	30	50	43	48	115	48	95	67	115	46
NAC	1	5	8	26	26	29	65	35	36	36	58	27
MADS-box	4	12	2	8	19	22	51	29	33	26	44	15
bHLH	4	7	11	29	23	23	65	18	21	20	30	10
Cupin_1	0	12	23	23	7	10	78	8	35	25	29	8
POT	0	23	24	20	9	13	26	26	53	33	27	29
SAUR	1	7	6	19	14	12	27	31	47	27	37	45
HMA	1	9	25	17	9	8	35	25	32	14	31	14
NPH3	6	5	6	31	27	20	31	20	22	22	30	24
PE	9	12	7	18	17	23	51	10	15	11	8	14
HSP20	4	13	13	15	8	8	23	21	26	12	7	1
LOB	1	4	8	15	8	5	24	15	34	8	13	7
GATA	2	2	2	9	14	12	21	15	15	14	26	4
GH3	0	11	2	2	5	10	40	16	16	7	19	7

Supplementary Table 11. The content detection of lignin and compositions in four tissues (xylem, phloem, sclerenchymatic belt, and spore).

**Tissue**	**Acid-insoluble lignin (% CWR)**	**Acid soluble lignin (% CWR)**	**H (μmol/g CWR)**	**G (μmol/g CWR)**	**S (μmol/g CWR)**
Xylem	36.30±0.02	3.62±0.01	7.10±0.05	305.94±0.21	0.12±0.001
Phloem	10.22±1.17	4.50±0.02	N.D.	1.10±0.02	0.06±0.01
Sclerenchymatic belt	14.55±1.03	2.24±0.04	N.D.	0.32±0.03	0.35±0.02
Spore	54.38±0.35	2.97±0.01	9.69±0.24	104.05±1.56	0.39±0.16

Supplementary Table 12. Summary of genetic variation in 6 groups.

Category	Nepal	SC/GX/GZ	FJ/TW	YN	HN	XZ
**Sequence variants**						
SNPs	5,822,085	17,554,121	16,725,788	38,967,231	11,294,576	10,584,708
Indels (<10 bp)	821,522	1,280,228	1,195,712	4,346,215	911,058	910,829
**Variants with effects on genes**						
SNPs that introduce stop codons	546	1,388	1,433	5,657	919	903
SNPs that disrupt stop codons	63	211	101	481	111	115
SNPs that induce alternative splicing	1,158	4,279	3,470	17,089	2,361	2,269
Indels located in genic regions	721,495	1,325,424	1,239,420	4,503,329	942,022	942,948
Frameshift indels	1,252	2,335	2,143	9,694	1,638	1,655
Genes affected by large-effect variants	2,149	4,733	4,520	18,521	3,166	3,143
Nonsynonymous variants	16,186	46,594	46,842	153,526	26,709	28,189
Synonymous variants	9178	27,618	27,046	108,037	15,364	15,982

Supplementary Table 13. Summary of the variants in 6 groups.

Type	Subgroups	Number of re-sequenced accessions	Ratio of non-synonymous to synonymous SNPs
Nepal	Nepal	12	1.7019
SC/GZ/GX	SiChuan	12	1.6759
	GuiZhou	12	1.6995
	GuangXi	12	1.692
FJ/TW	FuJian	12	1.7078
	TaiWan	12	1.711
YN	YunNan	12	1.4259
HN	HaiNan	12	1.7467
XZ	XiZang	11	1.7175

Supplementary Table 14. Fixation index (*Fst*) among 6 groups.

**Population**	**Nepal**	**SC/GZ/GX**	**FJ/TW**	**YN**	**HN**	**XZ**
Nepal						
SC/GZ/GX	0.221					
FJ/TW	0.26888	0.18975				
YN	0.69679	0.73698	0.71212			
HN	0.33749	0.15285	0.19037	0.68319		
XZ	0.30071	0.17034	0.22975	0.67747	0.24102	

Supplementary Table 15. GO and KEGG enrichment analysis of the protein-coding genes that are under selection. BP: biological process; CC: cellular component; MF: molecular function. Q-value less than 0.05 represented significant enrichment. P values were calculated using two-sided Fisher's exact test and further corrected based on Benjamini-Hochberg false discovery rate correction method.

GO enrichment					
**Category**	**GO ID**	**GO term**	**P-value**	**Q-value**	**Gene ratio (%)**
MF	GO:0008195	phosphatidate phosphatase activity	0.000	0.000	0.012
MF	GO:0004345	glucose-6-phosphate dehydrogenase activity	0.000	0.001	0.012
BP	GO:0009051	pentose-phosphate shunt, oxidative branch	0.000	0.001	0.012
BP	GO:0034517	ribophagy	0.000	0.001	0.012
BP	GO:0035017	cuticle pattern formation	0.000	0.001	0.012
MF	GO:1905538	polysome binding	0.000	0.001	0.012
CC	GO:1990861	Ubp3-Bre5 deubiquitination complex	0.000	0.001	0.012
BP	GO:0006098	pentose-phosphate shunt	0.000	0.002	0.012
BP	GO:0060628	regulation of ER to Golgi vesicle-mediated transport	0.000	0.002	0.012
BP	GO:0006414	translational elongation	0.000	0.003	0.010
BP	GO:0006739	NADP metabolic process	0.000	0.004	0.014
MF	GO:0000981	DNA-binding transcription factor activity, RNA polymerase II-specific	0.000	0.004	0.036
BP	GO:1901957	regulation of cutin biosynthetic process	0.000	0.004	0.012
BP	GO:0071497	cellular response to freezing	0.000	0.006	0.014
BP	GO:0061912	selective autophagy	0.000	0.008	0.012
BP	GO:1990019	protein storage vacuole organization	0.000	0.009	0.008
BP	GO:0032212	positive regulation of telomere maintenance via telomerase	0.000	0.012	0.008
BP	GO:0070125	mitochondrial translational elongation	0.000	0.012	0.008
BP	GO:1904358	positive regulation of telomere maintenance via telomere lengthening	0.000	0.012	0.008
MF	GO:0047714	galactolipase activity	0.000	0.013	0.014
BP	GO:0032543	mitochondrial translation	0.000	0.013	0.010
MF	GO:0008135	translation factor activity, RNA binding	0.000	0.014	0.016
BP	GO:0009751	response to salicylic acid	0.000	0.014	0.036
CC	GO:0071020	post-spliceosomal complex	0.000	0.017	0.012
CC	GO:0071021	U2-type post-spliceosomal complex	0.000	0.017	0.012
BP	GO:0140053	mitochondrial gene expression	0.000	0.017	0.012
BP	GO:0005996	monosaccharide metabolic process	0.000	0.017	0.024

BP	GO:0019318	hexose metabolic process	0.000	0.017	0.020
BP	GO:0000350	generation of catalytic spliceosome for second transesterification step	0.000	0.017	0.012
MF	GO:0003746	translation elongation factor activity	0.000	0.017	0.008
BP	GO:0034605	cellular response to heat	0.000	0.017	0.020
BP	GO:1901371	regulation of leaf morphogenesis	0.000	0.017	0.008
BP	GO:0019682	glyceraldehyde-3-phosphate metabolic process	0.000	0.020	0.012
MF	GO:0008970	phospholipase A1 activity	0.001	0.021	0.014
BP	GO:0032210	regulation of telomere maintenance via telomerase	0.001	0.022	0.010
BP	GO:0048736	appendage development	0.001	0.022	0.010
BP	GO:0090596	sensory organ morphogenesis	0.001	0.022	0.010
MF	GO:0004806	triglyceride lipase activity	0.001	0.022	0.014
BP	GO:0045292	mRNA cis splicing, via spliceosome	0.001	0.024	0.012
BP	GO:1904356	regulation of telomere maintenance via telomere lengthening	0.001	0.025	0.010
BP	GO:0048235	pollen sperm cell differentiation	0.001	0.026	0.012
BP	GO:0000018	regulation of DNA recombination	0.001	0.027	0.020
BP	GO:0000002	mitochondrial genome maintenance	0.001	0.027	0.022
BP	GO:0050826	response to freezing	0.001	0.027	0.014
BP	GO:0045910	negative regulation of DNA recombination	0.001	0.030	0.018
BP	GO:0000393	spliceosomal conformational changes to generate catalytic conformation	0.001	0.039	0.012
BP	GO:0006006	glucose metabolic process	0.001	0.039	0.014
BP	GO:0006081	cellular aldehyde metabolic process	0.001	0.039	0.018
BP	GO:0046677	response to antibiotic	0.001	0.041	0.048
BP	GO:0007423	sensory organ development	0.002	0.047	0.010
BP	GO:0043171	peptide catabolic process	0.002	0.047	0.010

**KEGG enrichment**				
**Pathway**	**Ko ID**	**P-value**	**Q-value**	**Gene ratio (%)**
Regulation of lipolysis in adipocytes	ko04923	0.000	0.033	0.014
Glutathione metabolism	ko00480	0.000	0.033	0.031
Glycerolipid metabolism	ko00561	0.000	0.042	0.035

Supplementary Table 16. Primers used in this study for qRT-PCR and gene cloning.

**Gene name**	**Forward Sequence (5'→3')**	**Reverse Sequence (5'→3')**
qRT-PCR		
AspiPAL3	ATGGTTCAGACCTACTTTGAGTCAACAG	CGAGGGTGACGATAGCTGCTTCTCC
AspiCCR2b	TGCCATTAATCATCCTCTCTTGCCA	TGATTAGAAAGCAAGGATGGAGGGAAA
AspiHCT40	CTGGTTCCCTGGCGTTACCA	AGGGCGTGCACAAGTAGGAG
AspiHCT101	TGCTAAGCCTACCAGGAGACAC	CCTCTGCAGGATCTTTCTCCGT
AspiC4H4	GCCCGGTCTTAATGTGTTCGC	TCCGCCGACTCTGTCAAACA
AspiCSE6	GCCTGCGAGGAGCAAGAGTA	GCAGATGCAAGCGGATGGTG
AspiCAD19	GTTCGTGTAAAGCACACTGCCA	AGGCCCAACTGCAGTAACCA
Aspi4CL28	CATCCTTGACAGAGGTCATGTGC	TGGAGACATTGGGAAATCGTGCT
Aspi4CL43	GGCATCCTTCTCCCTCTGGTC	CCCTCCCACATCCACTGTCA
AspiCCR10	GGGAACCTAGCACTTCCTTG	CCACAGATTTGGACGTCGCTT
AspiCCoAOMT2	ACCATGGCACCAACTCACCGCTAACC	GGAAGACCGCAACAACAACAGG
AspiCOMT2	CTCTCCACAGCTGCCGGTTT	GCAACACCTAGGCCTTATAAAGTTCC
AspiC3H3	GAATCATCGTCGGCAGCAGA	CTGTGGAACCGCATGTCACG
AspiCCoAOMT3a	GGTTCCCATTGGAGACGGGAT	TGTGTGCCATAAATAGCCCTTGC
AspiCOMT16	GTGTTGGAAGGGCGTGTTCG	TGCATTCCGGCAAGCGTAAC
AspiCAD32	TGCCATTCGGACCTTCACCA	AGCCCTTCACCTTGGAACCC
AspiHCT85	GTGCAAGGGCTGCCAATACC	TCAGGCTTTGGAGGTGGCTT
AspiHCT64	GCTAGCTTCTGCAGCTTGGTATG	ATGGCGGCTTGGAACAATGG
AspiCSE18	CCAAGGGTGTGGCCTCTCAG	AGACTCTCCAAACAGAAAGCAAGGA
Aspi4CL5	AGAGAGCAAGCCTAATCGGAACT	AGAGCATCACGAATGTTAGCACAC
AspiHCT1a	ACAGATCCAACCTCCTGCACTC	CCTCACAGACTTTAGGTTATGAGG
AspiHCT1b	CACCAGCTGAGGAGACACCA	GAAGCCTCCCAGCCATTGGA
AspiPAL4	CACTGGCTGAGAATTCCTGCAC	GGACGAGATAAGGGAGCTGCTATC
Aspi4CL47	GCTGTAAGGAGGGATAGTTGTAAG	GTGGTAACTTCCCAACTCAAGC
AspiCCR5a	CTTCGTCTCTTGCTGCACGC	GAGAAGGTGTAGTCGCCATAGC
AspiCSE3a	GGTATTCCAAGACATCATTGCG	CACATGCTGCATGGATGGAG
AspiCCR5b	GGTGTTGCACTCAACGGCATCTATG	AGATGGAGATGCGGAGCTTG
Aspi4CL1b	GTTCCCACGGGGCCAATTCAA	CAGCTTGGTCAAGCTACTCCTAAC
AspiC4H1	CAAACCCCTCTAACCTGCCTCCTT	CAAGAGCACTTCAAGACCTACAAG
Aspi01Gene06595 (*AspiActin*)	CTTTTCAATCCCACACTTCCACAG	GCAACATTGCCAGGCAAATGCTC
Gene Cloning		
Aspi01Gene02765	TGGTGGTGGTGGAATTCTAGAC**ATG**ACT	CAGTCACGATGAATTAAGCTT**TTA**ACTG

(*AspiPKS1*)	GTCTCTGAGAGTGCACG	AGAGGCACACTCCTGA
Aspi01Gene44865 (*AspiPKS2*)	TGGTGGTGGTGGAATTCTAGAC**ATG**CCG ATTCCTTCCGGCTCCT	CAGTCACGATGAATTAAGCTT**TCA**ATTA GTGAGAGGCACGCT
Aspi01Gene51163 (*AspiPKS3*)	TGGTGGTGGTGGAATTCTAGAC**ATG**ACC ATCGCTGAGTCTGGC	CAGTCACGATGAATTAAGCTT**TTA**ACTG GCGAGCGGGACACTGCG
Aspi01Gene15356 (*AspiPKS4*)	TGGTGGTGGTGGAATTCTAGAC**ATG**TTT CTGGAGGTGGAAGTTG	CAGTCACGATGAATTAAGCTT**TTA**CTTG CACCGCCTGAGGAGG
Aspi01Gene15357 (*AspiPKS5*)	TGGTGGTGGTGGAATTCTAGAC**ATG**TTT CTGGAGGTGGAAGTTG	CAGTCACGATGAATTAAGCTT**TCA**TTTG CACCGCTTGAGAAG
Aspi01Gene19041 (*AspiPKS6*)	TGGTGGTGGTGGAATTCTAGAC**ATG**TTT CTGGAGGTCGAGGTAG	CAGTCACGATGAATTAAGCTT**TTA**ATCT GATGCAGGCTTGC
Aspi01Gene18241 (*AspiPKS7*)	TGGTGGTGGTGGAATTCTAGAC**ATG**TTT CTGGAGATCGAAGTGG	CAGTCACGATGAATTAAGCTT**TCA**TTTG CACCGCTTGAGGAG
Aspi01Gene01559 (*AspiPKS8*)	TGGTGGTGGTGGAATTCTAGAC**ATG**GA GTGCAATAGTGTGCAC	CAGTCACGATGAATTAAGCTT**TCA**TTGA TGTGTTACCTTTCGA

Supplementary Table 17. The pfam numbers and blastp queries for gene family identification in this study.

Category	Gene family	Pfam number	Blastp query	Query species
TF	MADS-box	PF00319	-	-
	NAC	PF02365	-	-
Lignin	PAL	PF00221	-	-
	4CL	PF00501	-	-
	C4H	PF00067	Potri.019G130700.1 (*PtrC4H2*)	*Populus trichocarpa*
	C3H		Potri.006G033300.1 (*PtrC3H3*)	*Populus trichocarpa*
	CAld5H		Potri.007G016400.1 (*PtrCAld5H2*)	*Populus trichocarpa*
	CCoAOMT	PF01596	-	-
	CAD	PF08240<br>PF00107	-	-
	CCR	PF01370	-	-
	COMT	PF00891	-	-
	CSE	PF12146	-	-
	HCT	PF02458	-	-
Flavonoid	CHS	PF00195<br>PF02797	*AtCHS* (AT5G13930)	*Arabidopsis thaliana*
	CHI	-	*AtCHI* (AT3G55120)	*Arabidopsis thaliana*
	FLS	-	*AtFLS1* (AT5G08640)	*Arabidopsis thaliana*
	F3H	-	*AtF3H* (AT3G51240)	*Arabidopsis thaliana*
	F3′H	PF00067	Rhsim07G0001300.1	*Rhododendron simsii*
	F3′5′H		A6XHG1 (UniprotKB)、E9P1T5 (UniprotKB)	*Vitis vinifera*、*Solanum tuberosum*
	FNS		LjFNSII-1.1、LjFNSII-2.1	*Lonicera japonica*
	GT	PF00201	-	-
PKS	PKSIII	-	*VvSTS* (GenBank: CAA54221)	*Vitis vinifera*
			PmSPS1 (GenBank: QCX36371)	*Piper methysticum*
DNA methylation	DRM	-	*DRM2* (AT5G14620)	*Arabidopsis thaliana*
	MET	-	*MET1* (AT5G49160)	*Arabidopsis thaliana*
	CMT	-	CMT2 (AT4G19020)	*Arabidopsis thaliana*

Supplementary Table 18. Statistics of known and novel miRNAs in *A. spinulosa.*

miRNA	Type	Leaf-1	Leaf-2	Leaf-3	Total
Known miRNAs	Mapped mature	142	163	145	182
	Mapped hairpin	326	399	386	453
	Mapped uniq sRNA*	479	565	465	1509
	Mapped total sRNA	1,063,675	996,680	801,399	2,861,754
Novel miRNAs	Mapped mature	181	181	181	181
	Mapped hairpin	181	181	181	181
	Mapped uniq sRNA*	1028	1054	1023	3105
	Mapped total sRNA	489,139	535,545	430,116	1,454,800

*indicated categories of mapped sRNA.

Supplementary Table 19. Read counts of four kinds of ncRNAs in *A. spinulosa*.

Type	Leaf-1	Leaf-2	Leaf-3	Average	Percent (%)
rRNA	1,446,727	1,689,607	1,606,728	1,581,021	93.11
tRNA	93,019	124,467	102,944	106,810	6.29
snRNA	9,152	10,727	10,690	10,190	0.60
snoRNA	0	0	0	0	0

Supplementary Table 20. Energies of the conformers of compound (+)-alsophilin at B3LYP/6-311g (d,p) in MeOH.

Label	Conformer	Boltzmann weighting factor
1	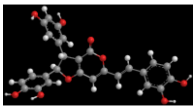	69.46
2	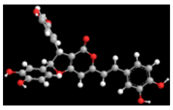	28.87
3	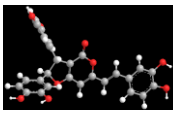	16.71
4	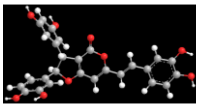	14.75
5	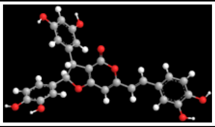	9.79
6	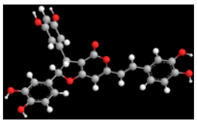	7.63
7	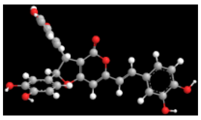	5.96
8	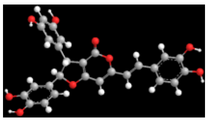	5.68
9	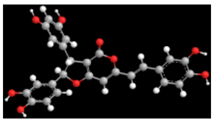	4.96
10	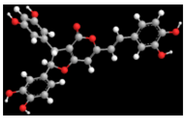	4.46
11	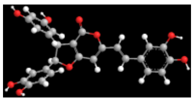	2.42
12	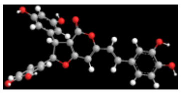	1.61
13	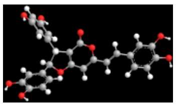	1.27

Supplementary Table 21. ^{1}H (500 MHz) and ^{13}C NMR (125 MHz) spectral data of (±)-alsophilin in CD_3OD.

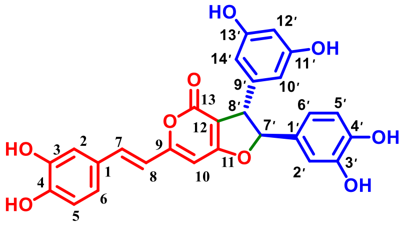

No.	δ_H (mult, J in Hz)	δ_C	No.	δ_H (mult, J in Hz)	δ_C
H1		128.7	P1′		132.5
H2	7.06 (d, 2.0)	114.9	P2′	6.76 (d, 2.0)	113.8
H3		146.8 [a]	P3′		146.9 [a]
H4		148.9	P4′		147.3
H5	6.78 (d, 8.5)	116.6	P5′	6.79 (d, 8.5)	116.4
H6	6.97 (dd, 8.5, 2.0)	122.2	P6′	6.66 (d, 8.5, 2.0)	119.0
H7	7.38 (d, 16.0)	138.1	P7′	5.43 (d, 6.5)	97.8
H8	6.70 (d, 16.0)	117.0	P8′	4.26 (d, 6.5)	55.0
H9		164.9	P9′		144.7
H10	6.43 (s)	96.3	P10′/14′	6.12 (d, 2.5)	106.7
H11		173.8	P11′/13′		160.1
H12		103.8	P12′	6.18 (t, 2.5)	102.6
H13		163.1			

[a] Overlapping signals.

Supplementary Table 22. Antioxidant activities on MDA production of the bioactive compounds.

Compound	Inhibition rate (%) [a]	IC_{50} values (μM)[b]
(±)-alsophilin	92.87±0.74	3.42±1.22
(−)-alsophilin	94.56±2.25	2.21±0.14
(+)-alsophilin	92.02±1.98	3.53±0.30
Piceatannol	97.74±1.00	0.61±0.18
Cyperusphenol B	96.81±2.49	1.52±0.41
Curcumin [c]	98.58±0.61	1.92±0.57

[a] Inhibition rate at 10^{-5} M. [b] IC_{50} values represent: mean ± SD (*n*=3 biologically independent experiments). [c] Positive control.
