# Supplementary material for: The flying spider-monkey tree fern genome provides insights into fern evolution and arborescence
Source: Nat Plants. 2022 May 9;8(5):500–12. doi: 10.1038/s41477-022-01146-6 (PMC9122828; doi:10.1038/s41477-022-01146-6)
Supplement: Supplementary file 2 — Reporting Summary [file 41477_2022_1146_MOESM2_ESM.pdf]

nature portfolio

Corresponding author(s): Quanzi Li

Last updated by author(s): Mar 26, 2022

# Reporting Summary

Nature Portfolio wishes to improve the reproducibility of the work that we publish. This form provides structure for consistency and transparency in reporting. For further information on Nature Portfolio policies, see our Editorial Policies and the Editorial Policy Checklist.

## Statistics

For all statistical analyses, confirm that the following items are present in the figure legend, table legend, main text, or Methods section.

| n/a | Confirmed | |
|---|---|---|
| ☐ | ☒ | The exact sample size (*n*) for each experimental group/condition, given as a discrete number and unit of measurement |
| ☐ | ☒ | A statement on whether measurements were taken from distinct samples or whether the same sample was measured repeatedly |
| ☐ | ☒ | The statistical test(s) used AND whether they are one- or two-sided<br>*Only common tests should be described solely by name; describe more complex techniques in the Methods section.* |
| ☒ | ☐ | A description of all covariates tested |
| ☒ | ☐ | A description of any assumptions or corrections, such as tests of normality and adjustment for multiple comparisons |
| ☐ | ☒ | A full description of the statistical parameters including central tendency (e.g. means) or other basic estimates (e.g. regression coefficient) AND variation (e.g. standard deviation) or associated estimates of uncertainty (e.g. confidence intervals) |
| ☐ | ☒ | For null hypothesis testing, the test statistic (e.g. *F*, *t*, *r*) with confidence intervals, effect sizes, degrees of freedom and *P* value noted<br>*Give P values as exact values whenever suitable.* |
| ☒ | ☐ | For Bayesian analysis, information on the choice of priors and Markov chain Monte Carlo settings |
| ☒ | ☐ | For hierarchical and complex designs, identification of the appropriate level for tests and full reporting of outcomes |
| ☐ | ☒ | Estimates of effect sizes (e.g. Cohen's *d*, Pearson's *r*), indicating how they were calculated |

*Our web collection on statistics for biologists contains articles on many of the points above.*

## Software and code

Policy information about availability of computer code

| | |
|---|---|
| Data collection | DNA-seq raw NGS data were generated by sequencing using Illumina HiSeq X-10 platform. DNA-seq raw PacBio data were generated by sequencing using PacBio sequel I/II platforms. Hi-C data were obtained by sequencing on Illumina Novaseq 6000 platform. RNA-seq raw data were generated by sequencing using Illumina HiSeq 4000 platform, and ISO-seq data were obtained by sequencing on PacBio Sequel platform. |
| Data analysis | All software employed in this study are publicly available from the internet and they are described in detail on the section of method, including the versions, parameters, and citations.<br>1. Canu v1.9 https://github.com/marbl/canu<br>2. Juicer v1.6 https://github.com/aidenlab/juicer<br>3. 3D-DNA v180922 https://github.com/aidenlab/3d-dna<br>4. pilon v1.23 https://github.com/broadinstitute/pilon<br>5. BWA-MEM v2.2.1 https://github.com/bwa-mem2/bwa-mem2<br>6. Jellyfish v2.1.3 https://github.com/gmarcais/Jellyfish<br>7. Samtools v1.13 https://github.com/samtools/samtools<br>8. HISAT2 v2.1.0 http://daehwankimlab.github.io/hisat2/download/<br>9. LAI v2.9.0 https://github.com/oushujun/LTR_retriever<br>10. BUSCO v5.2.2 https://busco.ezlab.org/<br>11. DESeq2 v1.34.0 https://github.com/mikelove/DESeq2<br>12. Tandem Repeats Finder v4.09 https://github.com/Benson-Genomics-Lab/TRF<br>13. RepeatModeler v2.0.1 http://www.repeatmasker.org/RepeatModeler/<br>14. RepeatMasker v4.1.0 http://repeatmasker.org/<br>15. minimap2 v2.17(r941) https://github.com/lh3/minimap2<br>16. Genewise v2.4.1 https://www.ebi.ac.uk/seqdb/confluence/display/THD/GeneWise<br>17. AUGUSTUS v3.2.2 https://github.com/Gaius-Augustus/Augustus<br>18. Geta v2.4.13 https://github.com/chenlianfu/geta |

19. Eggnog v2.1.5 https://github.com/eggnogdb/eggnog-mapper
20. Bismark v16.3 https://github.com/FelixKrueger/Bismark
21. OrthoFinder v2.5.4 https://github.com/davidemms/OrthoFinder
22. MAFFT v7.471 https://github.com/GSLBiotech/mafft
23. trimAl v1.4.1 https://github.com/inab/trimal
24. modeltest-ng v0.1.7 https://github.com/ddarriba/modeltest
25. RAxML-ng v1.1.0 https://github.com/amkozlov/raxml-ng
26. CAFE v4.2.1 https://github.com/hahnlab/CAFE
27. r8s v1.71 https://sourceforge.net/projects/r8s/
28. HMMER v3 https://github.com/kblin/bioperl-hmmer3
29. MUSCLE v3.8.1551 https://github.com/rcedgar/muscle
30. MCSCANX v0.8.46 https://github.com/wyp1125/MCScanX
31. wgd v1.1.1 https://github.com/arzwa/wgd
32. SOAPdenovo-Trans v1.0.4 https://github.com/aquaskyline/SOAPdenovo-Trans
33. CD-HIT v4.8.1 https://github.com/weizhongli/cdhit
34. R v4.0.5 https://www.r-project.org
35. Trinity v2.9.1 https://github.com/wlanjie/trinity
36. PAML v4.10.3 https://github.com/abacus-gene/paml
37. Fastqc v0.11.9 https://github.com/s-andrews/FastQC
38. Bowtie2 v2.4.1 https://github.com/BenLangmead/bowtie2
39. GATK v4.1.9 https://gatk.broadinstitute.org/hc/en-us
40. SnpEff v3.6c http://pcingola.github.io/SnpEff
41. VCFtools v0.1.16 https://github.com/vcftools/vcftools
42. SweeD v3.2.1 https://cme.h-its.org/exelixis//web/software/sweed/index.html
43. iTOL v6 https://itol.embl.de
44. GCTA v1.93.2 https://yanglab.westlake.edu.cn/software/gcta
45. PLINK v1.90 http://www.cog-genomics.org/plink2
46. Admixture v1.3.0 https://github.com/jacahill/Admixture
47. STRUCTURE v2.3.4 https://web.stanford.edu/group/pritchardlab/structure.html
48. stairway-plot-2 v2 https://github.com/xiaoming-liu/stairway-plot-v2
49. circos v0.69-8 http://circos.ca

For manuscripts utilizing custom algorithms or software that are central to the research but not yet described in published literature, software must be made available to editors and reviewers. We strongly encourage code deposition in a community repository (e.g. GitHub). See the Nature Portfolio guidelines for submitting code & software for further information.

## Data

Policy information about availability of data

All manuscripts must include a data availability statement. This statement should provide the following information, where applicable:

- Accession codes, unique identifiers, or web links for publicly available datasets
- A description of any restrictions on data availability
- For clinical datasets or third party data, please ensure that the statement adheres to our policy

The A. spinulosa genome project has been deposited at the National Genomics Data Center (https://ngdc.cncb.ac.cn/) under the BioProject number PRJCA006485, including genomic and transcriptomic data, HiC data, methylated data, small RNA data, and re-sequencing data under the GSA database ((http://gsa.big.ac.cn/)) with accessions of CRA005445, CRA005406, CRA005447, CRA005463, CRA005407, and CRA005430.

# Field-specific reporting

Please select the one below that is the best fit for your research. If you are not sure, read the appropriate sections before making your selection.

☒ Life sciences ☐ Behavioural & social sciences ☐ Ecological, evolutionary & environmental sciences

For a reference copy of the document with all sections, see nature.com/documents/nr-reporting-summary-flat.pdf

# Life sciences study design

All studies must disclose on these points even when the disclosure is negative.

| | |
|---|---|
| Sample size | For re-sequencing, we sampled 107 A. spinulosa individuals from 9 locations, having a good representativeness for each population (about 12 individuals). For phylogenetic analysis, bootstrapping values were set between 100-1000 times, which is the filed standard. The wall thickness of cells in sclerenchymatic belt and pith parenchyma were measured based on 86 cells. The length of the tracheids was measured by microscopy based on 45 tracheids. |
| Data exclusions | For phylogenetic analysis, protein sequences less than 50 amino acids that have an impact on phylogenetic tree construction were removed. |
| Replication | Three biological replicates were used in RNA-seq analysis, qRT-PCR, metabolite content determination, and antioxidation assays and all succeeded. |

| | |
|---|---|
| Randomization | A. spinulosa individuals were collected in the field, and the gametophytes were cultivated in growth chambers. All materials were selected randomly for experiments. |
| Blinding | Experiments were blinded and carried out by different coauthors or other researchers. |

# Reporting for specific materials, systems and methods

We require information from authors about some types of materials, experimental systems and methods used in many studies. Here, indicate whether each material, system or method listed is relevant to your study. If you are not sure if a list item applies to your research, read the appropriate section before selecting a response.

## Materials & experimental systems

| n/a | Involved in the study |
|---|---|
| ☒ | ☐ Antibodies |
| ☐ | ☒ Eukaryotic cell lines |
| ☒ | ☐ Palaeontology and archaeology |
| ☒ | ☐ Animals and other organisms |
| ☒ | ☐ Human research participants |
| ☒ | ☐ Clinical data |
| ☒ | ☐ Dual use research of concern |

## Methods

| n/a | Involved in the study |
|---|---|
| ☒ | ☐ ChIP-seq |
| ☒ | ☐ Flow cytometry |
| ☒ | ☐ MRI-based neuroimaging |

# Eukaryotic cell lines

Policy information about cell lines

| | |
|---|---|
| Cell line source(s) | U251, HepG2, MCF7, HCT116, RAW264.7 cell lines were purchased from the cell center of the Chinese Academy of Medical Sciences and Peking Union Medical College (Beijing, China). HGC27 cell line is a gift from Professor Shao Li (Tsinghua University,China). |
| Authentication | None of the cell lines used were authenticated. |
| Mycoplasma contamination | All cell lines tested negative for mycoplasma contamination. |
| Commonly misidentified lines (See ICLAC register) | No misidentified line was used. |
